# Ecological Stability Emerges at the Level of Strains in the Human Gut Microbiome

**DOI:** 10.1128/mbio.02502-22

**Published:** 2023-02-21

**Authors:** Richard Wolff, William Shoemaker, Nandita Garud

**Affiliations:** a Department of Ecology and Evolutionary Biology, UCLA, Los Angeles, California, USA; b Department of Human Genetics, UCLA, Los Angeles, California, USA; University of Illinois at Urbana-Champaign; University of California, Berkeley

**Keywords:** human gut microbiome, macroecology, ecology, metagenomics, strains

## Abstract

The human gut microbiome harbors substantial ecological diversity at the species level as well as at the strain level within species. In healthy hosts, species abundance fluctuations in the microbiome are thought to be stable, and these fluctuations can be described by macroecological laws. However, it is less clear how strain abundances change over time. An open question is whether individual strains behave like species themselves, exhibiting stability and following the macroecological relationships known to hold at the species level, or whether strains have different dynamics, perhaps due to the relatively close phylogenetic relatedness of cocolonizing lineages. Here, we analyze the daily dynamics of intraspecific genetic variation in the gut microbiomes of four healthy, densely longitudinally sampled hosts. First, we find that the overall genetic diversity of a large majority of species is stationary over time despite short-term fluctuations. Next, we show that fluctuations in abundances in approximately 80% of strains analyzed can be predicted with a stochastic logistic model (SLM), an ecological model of a population experiencing environmental fluctuations around a fixed carrying capacity, which has previously been shown to capture statistical properties of species abundance fluctuations. The success of this model indicates that strain abundances typically fluctuate around a fixed carrying capacity, suggesting that most strains are dynamically stable. Finally, we find that the strain abundances follow several empirical macroecological laws known to hold at the species level. Together, our results suggest that macroecological properties of the human gut microbiome, including its stability, emerge at the level of strains.

## INTRODUCTION

The human gut microbiome is a complex ecological community composed of tens of trillions of cells that interact directly and indirectly with one another and the host (1–3). Although the precise species compositions of the gut microbiome differ among hosts, healthy adult guts tend to be both ecologically diverse and temporally stable at the species level under normal circumstances (4–7). The ecological stability of the gut community is critical for the preservation of its functional capacity over time, and periods of instability and heightened variability are often associated with environmental perturbations or disease states (8–10).

Much as the gut community as a whole is made up of a diverse array of species, within species, populations of gut microbes harbor many genetic variants (11). A growing body of literature highlights the importance of interhost differences in microbiome genetic composition for various aspects of human health, with specific microbial genotypes and strains being associated with the digestion of certain foods (12), a range of host disease risk factors (13), bile and lipid composition (14), and antibiotic resistance (15). Recent studies have begun to characterize how this genotypic diversity changes over time within hosts (7, 11, 16, 17) and have linked longitudinal changes in genetic composition to specific host phenotypes and metabolite levels (18).

Broadly, dynamic changes in genetic variation in the gut microbiome occur at two distinct levels. First, there are changes in the frequencies of lineages that have clonally diverged since their common ancestor colonized the host due to the evolutionary forces of mutation, drift, selection, and recombination. Typically, such lineages differ from one another at a small number [O(1) to $O(10^{2})$] of single nucleotide variants (SNVs) (16, 17). Second, there are fluctuations in the relative abundances of conspecific strains that do not share an ancestor within the host. When levels of recombination between such strains are sufficiently low, clonal descendants of the initial colonizers may persist within the host as genetically distinguishable populations differing from one another at a number of sites in their shared, core genome [$O(10^{3})$ to $O(10^{4})$] similar to those of strains drawn from unrelated hosts (11, 16, 19–21). Multiple colonization by conspecific strains is evidently under some degree of ecological constraint as only a few strains (typically between one and four) are ever observed within a host at any one time, a phenomenon dubbed “oligocolonization” (16, 22–24). The mechanisms enabling a small number of strains to colonize a host and increase to a high frequency, but preventing a large number of exogenous strains from doing the same, are not yet known. Interestingly, similar colonization patterns have been observed for a number of other host-associated members of the microbiota, both at different human body sites (25) and in other organisms (26, 27).

In healthy, adult hosts, a large majority of strains persist over periods of months to years (7, 11, 16, 18, 28, 29). Moreover, strains within the gut can remain resilient in the face of large perturbations such as antibiotics (23) and fecal microbiome transplants (30). However, little is known about the magnitude of daily fluctuations in genetic composition at either the strain or the lineage level under ordinary conditions, or how such fluctuations ultimately affect the stability properties of the gut community, as longitudinal studies of genetic diversity in the gut have tended to focus on samples collected at multimonth intervals.

In this work, we seek to understand how the genetic composition of the gut changes over time, from daily to multiyear timescales, in four healthy, adult hosts sampled over the course of 6 to 18 months (31). To do so, we leverage concepts from macroecology to examine the dynamics of strains in these four hosts. Macroecology focuses on characterizing statistical regularities in patterns of abundance and diversity within and between ecological communities. A growing body of work has demonstrated that species-level patterns of diversity in a variety of natural microbial communities are well described by macroecological laws (32–35). Many of these macroecological laws can be recapitulated through intuitive ecological models containing few, if any, free parameters (32, 33, 36). Among these successful models is the stochastic logistic model (SLM), which describes the dynamics of a population experiencing rapid stochastic fluctuations induced by environmental noise around a fixed carrying capacity (37). Whether the populations making up a community exhibit regular, statistically quantifiable dynamics and, if so, whether these dynamics can be explained using simple models are fundamentally macroecological questions. In this work, we find not only that the large majority of strains in these healthy hosts exhibit abundance dynamics consistent with an SLM but also that strain abundance fluctuations follow several macroecological laws known to hold among species (32, 33, 35, 36).

Together, our results indicate that daily fluctuations in overall genetic composition within the gut microbiome are largely stationary and that these fluctuations follow broad macroecological patterns. Thus, several macroecological properties of the human gut known to hold at higher levels of taxonomic organization, including its stability, appear to emerge at the level of strains.

## RESULTS

To explore how daily fluctuations in nucleotide diversity and strain abundances translate into stability over periods of months to years, we used high-resolution temporal data from four hosts sampled in the BIO-ML project (31) (see Materials and Methods for further details on sampling).

### Temporal stability of intraspecific genetic variation.

Within hosts, allele frequencies change over time in gut microbial populations due to mutation, drift, selection, and fluctuations in the relative abundances of strains. While studies examining broad cohorts of sparsely longitudinally sampled individuals indicate that the magnitude of intrahost fluctuations only infrequently approaches that of interhost differences over timescales of months to years (7, 11, 16), more finely resolved temporal trends are less well characterized. To evaluate the stability of the gut community, it is crucial to determine whether temporal fluctuations are stationary or directional.

To assess the stability of intraspecific genetic variation over time, we examined temporal trends in the patterns of nucleotide diversity within our four hosts using *F_ST_*, a standard measure of genetic differentiation between populations (see Materials and Methods for further details on *F_ST_* calculations). If the genetic composition of a species changes directionally, we expect that samples drawn later in the time course will have a higher *F_ST_* value relative to the initial time point than earlier samples. If, in contrast, fluctuations are stationary, then later time points should on average be no more diverged from the initial sample than earlier time points.

To contextualize the magnitude of the variation in the genetic composition over time, we normalized our longitudinal measurements of *F_ST_* within hosts by the mean *F_ST_* value of the species across hosts. We calculated this species-wide mean *F_ST_* using shotgun metagenomic data from 250 North American hosts sampled in the Human Microbiome Project (6, 38), allowing us to better capture the extent of interhost diversity. We refer to the resulting normalized *F_ST_* statistic, obtained by dividing each intrahost *F_ST_* measurement by the mean *F_ST_* across hosts, as *F*_*ST*_^′^. *F*_*ST*_^′^ will approach or exceed a value of 1 when intrahost fluctuations are of the same magnitude as those of interhost differences and will remain close to a value of 0 when the genetic composition of the population is constant over time.

**FIG 1:**
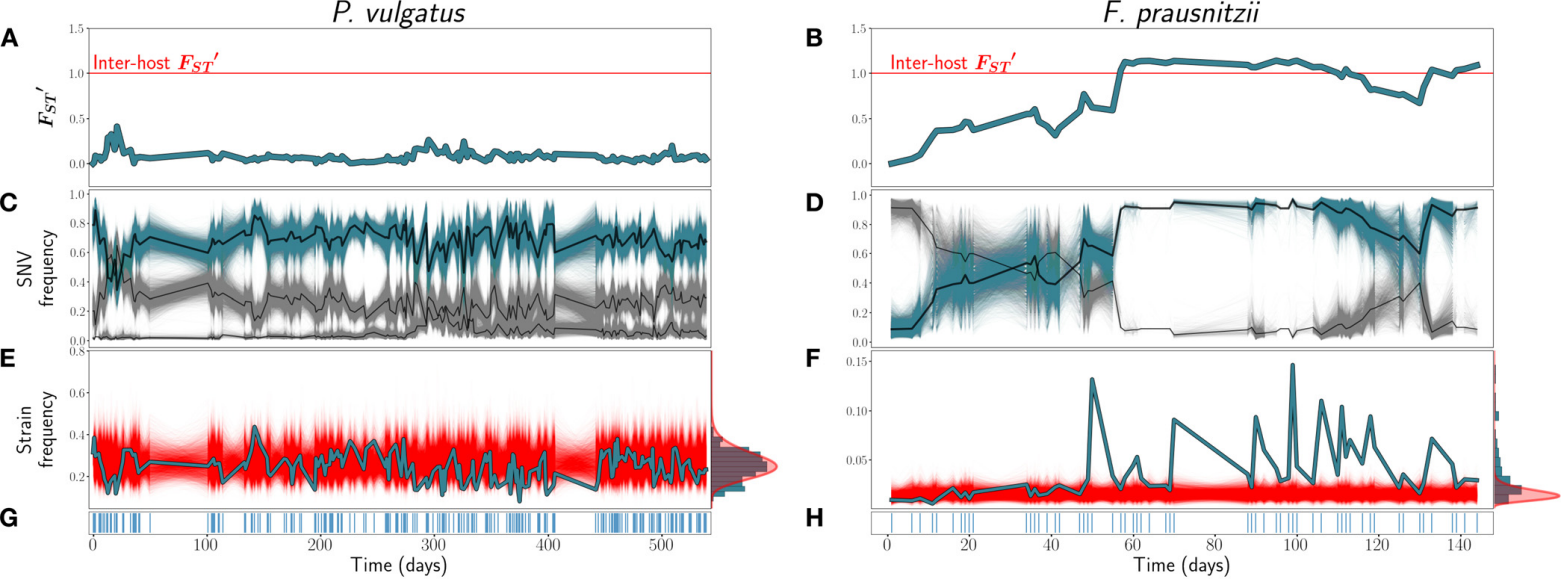
(A and B) $F_{\mathrm{ST}}{}^{\prime }$ trajectories for *P. vulgatus* (host *am*) (A) and *F. prausnitzii* (host *ao*) (B). (C and D) SNV frequencies of three inferred strains for *P. vulgatus* (C) and two inferred strains for *F. prausnitzii* (D). In black are the inferred strain trajectories. Highlighted in blue are example strains featured further in panels E and F. (E and F) Frequencies of the example strains in blue, with simulations of the corresponding SLM overlaid in red. At the right, the empirical distribution of strain abundances is plotted in blue, and the stationary gamma distribution of abundances (see equation 2) predicted by the SLM is in red. (G and H) Sampling time points. Blue lines indicate that a sample was taken on that day.

In line with previous work (7, 11), we observe that changes in the genetic composition within the hosts that we examine (*am*, *ao*, *an*, and *ae*) rarely approach the magnitude of interhost differences, as *F*_*ST*_^′^ remained well below 1 at all time points for all but one species examined (see Fig. S5 in Text S1 in the supplemental material). For the one aberrant species, Faecalibacterium prausnitzii in host *ao*, $F_{\mathrm{ST}}^{\prime }$ increases steadily before a rapid increase to above 1 at around the time point of 60 days (Fig. 1B). More typical, however, is the example of Phocaeicola vulgatus in host *am*, for which $F_{\mathrm{ST}}^{\prime }$ fluctuates but appears to remain near a long-term steady state (Fig. 1A).

10.1128/mbio.02502-22.1TEXT S1Further methodological and contextual details. (Table S1) Host sampling metadata. (Table S2) Reference genome length by species. (Table S3) Number of strains inferred for each species analyzed in host *am*. (Table S4) Number of strains inferred for each species analyzed in host *ao*. (Table S5) Number of strains inferred for each species analyzed in host *an*. (Table S6) Number of strains inferred for each species analyzed in host *ae*. (Fig. S1) Illustration of oligocolonization. (Fig. S2) Read and sampling metadata. (Fig. S3) Nucleotide diversity, π, for each species-host combination. (Fig. S4) $F_{\mathrm{ST}}{}^{\prime }$ by species and host, colored by stationarity. (Fig. S5) $F_{\mathrm{ST}}{}^{\prime }$ by species and host. (Fig. S6) Polarization when three strains are present. (Fig. S7) Taylor’s law: multinomial compositional sampling. (Fig. S8) Taylor’s law: variable-intensity multinomial compositional sampling. Download Text S1, PDF file, 2.8 MB.Copyright © 2023 Wolff et al.2023Wolff et al.https://creativecommons.org/licenses/by/4.0/This content is distributed under the terms of the Creative Commons Attribution 4.0 International license.

To determine quantitatively whether the fluctuations in genetic composition were stationary or directional, we implemented an augmented Dickey-Fuller (ADF) test for each $F_{\mathrm{ST}}^{\prime }$ time series (39). The ADF test tests the null hypothesis that a time series is nonstationary against the alternate hypothesis that the time series is stationary. Rejecting the null hypothesis with the ADF test is thus evidence that the mean and variance in a temporally varying quantity are time invariant. For 34 of the 45 species (76%) considered, we rejected the null hypothesis at a significance level of a *P* value of 0.05, indicating that the majority of species exhibit stationary $F_{\mathrm{ST}}^{\prime }$ trends.

Our results suggest that fluctuations in allele frequencies are typically stable, at least when coarse-grained across the whole genome, in these hosts. However, large-scale, rapid changes in allele frequency, as observed for F. prausnitzii, also occur.

### Strain frequencies.

Recent empirical studies using isolate, single-cell, and shotgun sequencing data have demonstrated that at any one time, the human gut microbiome is colonized by at most a few distinct conspecific strains (between 1 and 4), which do not share a clonal ancestor within the host (16, 20, 24, 29, 31). Much less is known, however, about the dynamics of strains once they have colonized the host.

To investigate these dynamics, we infer strain genotypes and frequencies using an algorithm adapted from the one described previously by Roodgar et al. (23). This algorithm identifies large clusters of SNVs with tightly correlated frequency trajectories (23), indicative of linkage on a common genomic background. By identifying large clusters, we expect to distinguish the trajectories of deeply diverged strains (for further information on our strain phasing, see Text S1, section 3.2). When no large cluster of tightly linked SNVs was detected within a species, we inferred that only a single strain was present.

Of the 45 species-host pairs examined, 15 (33.3%) harbored multiple strains. Of these 15 pairs, 13 were colonized by two strains, while in 2 separate hosts (*am* and *an*), the species P. vulgatus was composed of three strains. The number of fixed differences between strains varied between $O(10^{3})$ and $O(10^{4})$, although due to our conservative filters for both calling SNVs from reads and assigning SNVs to strains, these likely represent underestimates of the true divergence between these strains. For further discussion of interstrain genetic divergence, see Text S1, section 3.1.

As may be expected given our $F_{\mathrm{ST}}^{\prime }$ results, most strains, by visual inspection, exhibit frequency dynamics that are heuristically consistent with stationarity (see Texts S2 to S5 for the strain trajectories of all species). The relative frequencies of most strains appear to fluctuate around a constant value throughout the sampling period. In a typical example of this kind of behavior, the dominant strain of *P. vulgatus* in host *am* fluctuates at around a 60% frequency for more than 500 days (Fig. 1C). However, in a minority of cases, the strain frequencies shifted dramatically throughout the sampling period. The most striking example of this is *F. prausnitzii* in host *ao*, which we have already seen underwent fluctuations in $F_{\mathrm{ST}}^{\prime }$ (Fig. 1B) of the magnitude of interhost differences in genetic composition. In this species, an initially rare strain almost fully supplants the initially dominant strain within the span of 60 days before a partial reversion later in the time course (Fig. 1D). In another case, Parabacteroides distasonis in host *ao*, a single strain that initially falls below the detection threshold increases to a detectable abundance midway through the sampling time course (Text S3). Similarly, the minor strain of *P. vulgatus* in host *am* is initially very rare before rapidly increasing in frequency at around day 300 and subsequently reverting to an intermediate steady state below its maximum frequency. This subtle shift in strain frequencies is not, interestingly, detected by our ADF test of $F_{\mathrm{ST}}^{\prime }$ as a departure from stationarity, likely due to the fact that the absolute magnitude of the shift in allele frequencies between the beginning and the end of the time course is small (less than 5%).

10.1128/mbio.02502-22.2TEXT S2$F_{\mathrm{ST}}^{\prime }$, strain frequency, and strain abundance dynamics plots for all species analyzed in host *am*. These plots are analogous to the results in Fig. 1. When only a single strain was detected, only $F_{\mathrm{ST}}^{\prime }$ and strain abundance dynamics plots, but no strain frequency plot, are included. Download Text S2, PDF file, 2.7 MB.Copyright © 2023 Wolff et al.2023Wolff et al.https://creativecommons.org/licenses/by/4.0/This content is distributed under the terms of the Creative Commons Attribution 4.0 International license.

10.1128/mbio.02502-22.3TEXT S3$F_{\mathrm{ST}}^{\prime }$, strain frequency, and strain abundance dynamics plots for all species analyzed in host *ao*. These plots are analogous to the results in Fig. 1. When only a single strain was detected, only $F_{\mathrm{ST}}^{\prime }$ and strain abundance dynamics plots, but no strain frequency plot, are included. Download Text S3, PDF file, 1.6 MB.Copyright © 2023 Wolff et al.2023Wolff et al.https://creativecommons.org/licenses/by/4.0/This content is distributed under the terms of the Creative Commons Attribution 4.0 International license.

10.1128/mbio.02502-22.4TEXT S4$F_{\mathrm{ST}}^{\prime }$, strain frequency, and strain abundance dynamics plots for all species analyzed in host *an*. These plots are analogous to the results in Fig. 1. When only a single strain was detected, only $F_{\mathrm{ST}}^{\prime }$ and strain abundance dynamics plots, but no strain frequency plot, are included. Download Text S4, PDF file, 1.7 MB.Copyright © 2023 Wolff et al.2023Wolff et al.https://creativecommons.org/licenses/by/4.0/This content is distributed under the terms of the Creative Commons Attribution 4.0 International license.

10.1128/mbio.02502-22.5TEXT S5$F_{\mathrm{ST}}^{\prime }$, strain frequency, and strain abundance dynamics plots for all species analyzed in host *ae*. These plots are analogous to the results in Fig. 1. When only a single strain was detected, only $F_{\mathrm{ST}}^{\prime }$ and strain abundance dynamics plots, but no strain frequency plot, are included. Download Text S5, PDF file, 2.6 MB.Copyright © 2023 Wolff et al.2023Wolff et al.https://creativecommons.org/licenses/by/4.0/This content is distributed under the terms of the Creative Commons Attribution 4.0 International license.

10.1128/mbio.02502-22.6 DATA SET S1 Host and sampling metadata for the four hosts analyzed here. Download Data Set S1, XLSX file, 0.02 MB. Copyright © 2023 Wolff et al. 2023 Wolff et al. https://creativecommons.org/licenses/by/4.0/ This content is distributed under the terms of the Creative Commons Attribution 4.0 International license.

We further note the striking visual correspondence between the $F_{\mathrm{ST}}^{\prime }$ and strain frequency trajectories, as is evident in Fig. 1 for both *P. vulgatus* and *F. prausnitzii*. Because fluctuations in the relative frequencies of cocolonizing strains with respect to one another determine allele frequencies at a very large fraction of all polymorphic sites, genome-wide average diversity statistics like $F_{\mathrm{ST}}^{\prime }$ will reflect strain dynamics. This correspondence is therefore supporting evidence that the overwhelming majority of genetic variation in these species is due to fixed differences between strains rather than among lineages belonging to strains. However, as is evident from the example of the minor strain of *P. vulgatus* in host *am*, subtle but important strain dynamics can also be obscured when considering only genome-wide average patterns of diversity.

### Stochastic logistic model.

In the sections above, we have seen that the genetic compositions of most species examined exhibit stationary dynamics over the timescale of observation. We hypothesized that this behavior might result from the underlying strains fluctuating around fixed absolute carrying capacities.

To test this hypothesis, we assessed the fit and predictive capacity of the stochastic logistic model (SLM), a model of a population experiencing stochastic fluctuations around a fixed abundance. Recent work in microbial ecology has demonstrated the power of minimal models like the SLM, requiring the fit of no free parameters, to reproduce qualitative and quantitative features of natural microbial community dynamics (32, 33, 36, 37, 40). Here, we tested the capacity of the SLM to forecast future strain behavior when trained on an initial subset of time points. By training on only a subset of initial points, we can assess whether strain dynamics are consistent over time.

Under the assumptions of the SLM, each population, *i*, has a long-term carrying capacity, *K_i_*, and temporal fluctuations in abundance around this value are driven by environmental noise with amplitude σ*_i_*. The dynamics of a population governed by an SLM can be expressed with the following stochastic differential equation:

(1) $$\begin{array}{l} \frac{dx_{i}}{\mathrm{dt}}=\frac{x_{i}}{{\text{τ}}_{i}}\left(1-\frac{x_{i}}{K_{i}}\right)+\sqrt{\frac{{\text{σ}}_{i}}{{\text{τ}}_{i}}}x_{i}\text{η}(t) \end{array}$$

where ${\text{τ}}_{i}^{-1}$ is the growth rate and η(*t*) is a Brownian noise term.

Populations following an SLM may experience large fluctuations in abundance over short timescales and may even be temporarily found far from their long-term average value, but these deviations will be transient. Over long timescales, the observed distribution of abundances will converge to a stationary gamma distribution (32):

(2) $$\begin{array}{l} \text{ρ}(x_{i})=\frac{1}{\Gamma (2{\text{σ}}_{i}^{-1}-1)}{\left(\frac{2}{K_{i}{\text{σ}}_{i}}\right)}^{2{\text{σ}}_{i}^{-1}-1}x^{2{\text{σ}}_{i}^{-1}-2}\exp\left(-\frac{2}{K_{i}{\text{σ}}_{i}}x\right) \end{array}$$

To determine whether strain trajectories could be described by an SLM, we first obtained time series of strain abundances by multiplying the relative frequencies of the strains inferred in the previous section by the relative abundance of the species to which they belong.

Next, we estimated *K_i_* and σ*_i_* from the first one-third of time points for each strain. *K_i_* and σ*_i_* are not free parameters but rather are functions of the mean and variance of the observed abundances (for details, see Text S1, section 4). To assess quantitatively whether the time series of strains in our cohort could be adequately described by an SLM, we developed and implemented a goodness-of-fit test. This test determines whether the transitions between subsequent time points are consistent with an SLM (for further details, see Text S1, section 5). Qualitatively, if a strain follows an SLM, its average and variance in abundance in the latter two-thirds of the time series should match those of the former one-third, and the strain should have a tendency to revert to its carrying capacity, *K_i_*.

Returning to our case studies, we see that the dominant strain of *P. vulgatus* is well described by the SLM (Fig. 1E). The true abundance trajectory (in blue) explores largely the same space as that of the SLM simulations (in red). Moreover, the empirical distribution of abundances across the entire time course appears to approach the stationary gamma distribution (equation 2) predicted by the SLM (Fig. 1E, right). In contrast, the dynamics of the invasive strain of *F. prausnitzii* deviate strongly from the SLM (Fig. 1F).

**FIG 2:**
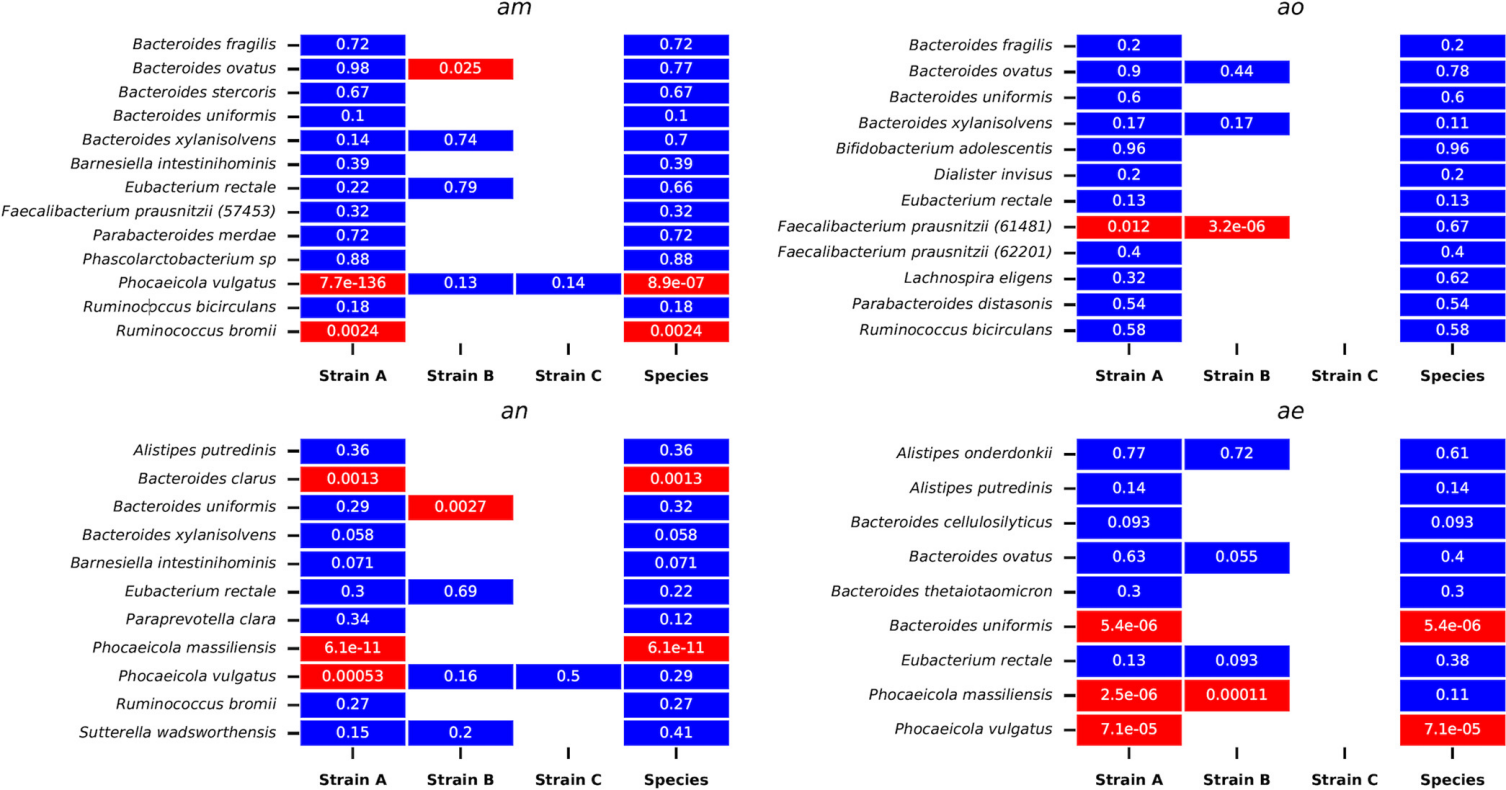
Results of the SLM goodness-of-fit test, by host. Totals of 79% (49/62) of strains and 86.6% (39/45) of species exhibit stochastic logistic dynamics across the sampling interval. The percentage of strains passing the test varied among hosts, with 83% passing in host *am*, 87% passing in *ao*, 75% passing in *an*, and 69% passing in *ae*. The *P* value associated with each strain or species is shown in white within each cell.

Overall, 79% (49/62) of strains passed our goodness-of-fit test, with 83% passing in host *am*, 86% passing in *ao*, 73% passing in *an*, and 69% passing in *ae* (Fig. 2). While the dominant strain of *P. vulgatus* passes, both strains of *F. prausnitzii* in host *ao*, as well as the minor strain in host *am*, fail the SLM. Thus, the SLM recapitulates the qualitative behavior of a large majority of strains even when trained on only a subset of initial points while also having the power to discriminate instances in which the dynamics of strains are evidently quite nonstationary.

Moreover, the likelihood of exhibiting SLM dynamics is independent of the presence of other strains. Of the 32 strains for which another conspecific strain was present, 24 (75%) passed the SLM test, while among the 30 singly colonizing strains, 25 (83%) passed the test. Thus, while singly colonizing strains tend to be moderately more likely to exhibit stable dynamics, the difference in pass rates is not statistically significant (χ^2^ = 0.24; *P* value = 0.62), indicating that the presence of conspecific strains is not *prima facie* destabilizing for a focal strain.

Next, we conducted an identical test of the SLM at the species level. Overall, 86% (39/45) of species exhibited dynamics consistent with an SLM. In the case of *F. prausnitzii*, for instance, the abundance of the species overall fluctuated stably, obeying the SLM. Thus, despite a partial replacement event, the total abundance of the species remained roughly constant. One interpretation of this observation is that these strains strongly compete with one another for the same species’ niche. Interestingly, *F. prausnitzii* is known to experience higher rates of replacement over multiyear timescales than other gut commensals, and these replacements are associated with alterations in the levels of plasma metabolites that affect host immunity (18). However, we emphasize that the replacement was only partial, and the “displaced” strain recovered temporarily to an intermediate abundance. This example highlights the complexity of strain dynamics as well as their potential relevance for host phenotypes.

Interestingly, none of the six species that exhibited non-SLM dynamics also failed our ADF test of intraspecific genetic stationarity. Of these six species, five harbored only a single strain, and in such cases, changes in the abundance of the strain do not imply changes in the levels of intraspecific genetic diversity, as they do in the multistrain case. The only species that exhibited non-SLM dynamics and harbored multiple strains was *P. vulgatus* in host *am*. As noted above, although the minor strain of this species exhibits highly non-SLM dynamics, contributing to the species as a whole failing the SLM test as well, the genome-wide average levels of genetic diversity as measured by $F_{\mathrm{ST}}^{\prime }$ change relatively little. Overall, examining changes in intraspecific genetic diversity and species abundance yields orthogonal information about population dynamics. For instance, the genetic composition of a species may change dramatically while the total abundance of the species fluctuates stably, as with *F. prausnitzii*. Conversely, a species’ abundance may change directionally while the genetic composition of the population remains roughly constant, as might occur during a rapid demographic expansion of a population with low initial diversity.

### Macroecology of strains.

Much as the dynamics of individual strains can largely be recapitulated with a single relatively simple model, we can also attempt to parsimoniously characterize patterns of variability across strains collectively. Such low-dimensional representations of complex community dynamics are the natural purview of macroecology, which attempts to characterize variation within and among communities by observing the statistical patterns of abundance, distribution, and diversity across their constituent members. In many kinds of microbial ecosystems, including the human gut, patterns of species abundance and distribution have been shown to broadly follow a number of macroecological laws, including Taylor’s law and a gamma distribution of abundance fluctuations (32, 33, 35). We show here that these macroecological relationships also characterize patterns of variation in the abundance of strains across our cohort.

The first pattern examined is power-law scaling between the mean and variance in abundance, known in ecology as Taylor’s law, which can be stated as:

(3) $$\begin{array}{l} {\text{σ}}_{x_{i}}^{2}\propto {\langle x_{i}\rangle }^{\text{α}} \end{array}$$

where $\langle x_{i}\rangle$ and ${\text{σ}}_{x_{i}}^{2}$ are the mean and variance of *x_i_*, respectively, and α is the scaling exponent of the power law.

**FIG 3:**
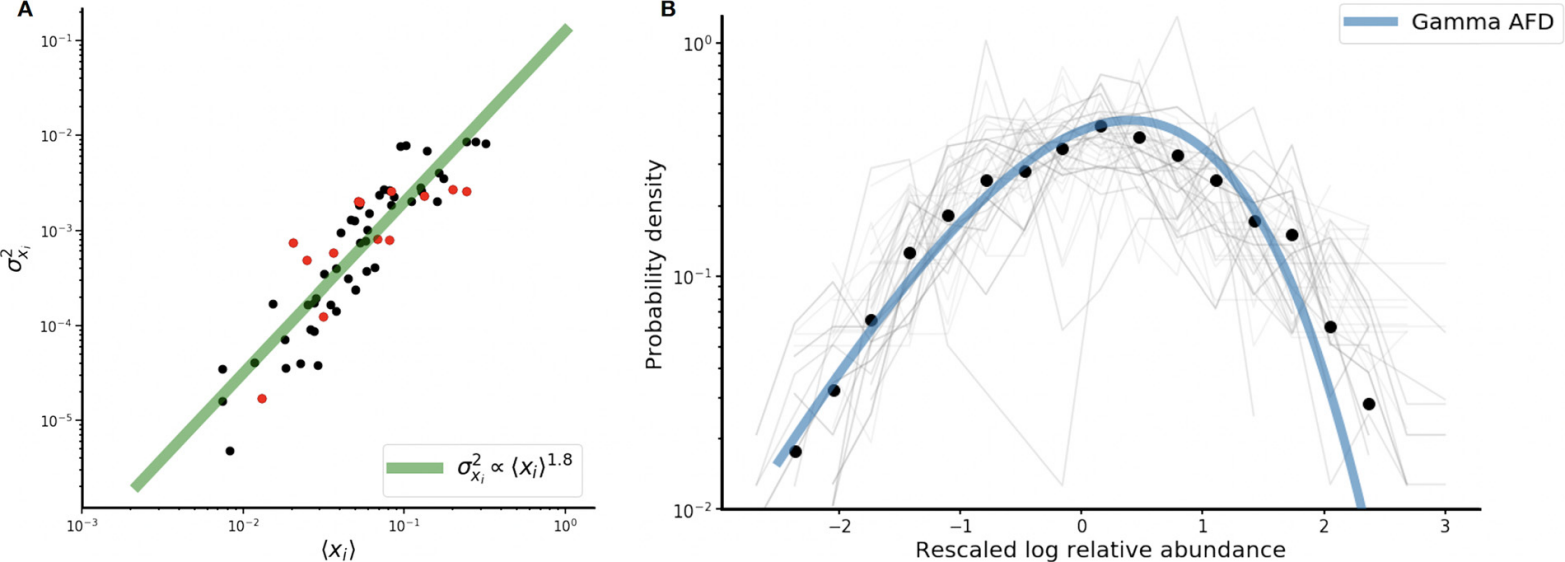
(A) Scaling of the variance in strain abundance with the mean abundance obeys Taylor’s law with an exponent of 1.8. Black dots are strains passing the SLM, while red dots are strains failing the SLM. (B) Strain abundances approximately follow a gamma distribution, which is the stationary distribution of the SLM. Black circles are the average probability densities of the rescaled abundances across all strains, and the blue line is the gamma fit of the bin means of the rescaled distributions. Light-gray lines are the individual rescaled abundance distributions for each strain individually (62 in total).

Many mechanisms can give rise to Taylor’s law. For instance, when the only source of variability between communities (or, in our case, longitudinal samples) is due to sampling noise, Taylor’s law exponent α will equal 1. In contrast, in communities where the scale of fluctuations is independent of abundance, that is, where all populations have identical per-capita fluctuations, α will equal 2 (32, 35) (for further details, see Text S1, section 6). We observed Taylor’s law scaling with an exponent of an α of 1.8 among all strains (Fig. 3A), mirroring previous findings at the species level (35). This Taylor’s law exponent indicates that higher-abundance strains are proportionally less variable than lower-abundance strains (α < 2) and that variation in strain abundance is not driven solely by sampling noise (α > 1) but rather reflects true, underlying biological variability. However, the existence of power-law scaling between mean and variance cannot, by itself, conclusively prove that any specific ecological model governs community dynamics. Indeed, the fit of the SLM does not depend on the existence of Taylor’s law scaling, or vice versa, as the SLM can hold with arbitrary mean and variance values.

The next pattern considered is the abundance fluctuation distribution (AFD), the overall distribution of abundances of a population over time. It is known that in a variety of microbial ecosystems, the AFD of many species tends to approach a gamma distribution (32). As discussed above, a population governed by stochastic logistic dynamics will tend toward a gamma distribution of abundances over long timescales (see, for instance, the histogram of abundances for the dominant strain of *P. vulgatus* in Fig. 1E, right). Given the generally excellent fit of the SLM to the population time series, the abundances of strains might generically be expected to each individually follow a gamma distribution. In Fig. 3B, we see that the distributions of strain abundances are indeed, on average, well described by a gamma distribution (black dots and blue line), although some individual strains (gray lines) deviate somewhat from the gamma stationary distribution. Recalling that the SLM of a given strain is uniquely determined by its mean and variance, it is apparent that the collapse of the AFDs to a single gamma distribution is in fact a consequence of the strong constraint that Taylor’s law places on these quantities across strains.

## DISCUSSION

In this study, we sought to characterize the within-species population dynamics of the human gut microbiomes of four healthy hosts. Previous efforts have shown that within-host changes in the genetic composition of gut microbial populations over time tend to be small compared to interhost differences (7, 11). We build on this result by demonstrating that at a daily temporal resolution, intraspecific diversity tends to fluctuate around a long-term average value within the hosts examined over periods of years. We show, crucially, that the abundance fluctuations of a large majority of strains that we detect can be predicted by the stochastic logistic model (SLM) of growth, a model that also recapitulates fluctuations at the species level (32, 33, 37). Finally, we find that empirical patterns of strain abundance variation in these hosts follow macroecological laws, which have also previously been demonstrated to hold at the species level, including Taylor’s law and a gamma abundance fluctuation distribution (32, 33, 35). Together, our results indicate that many of the broad properties of the gut observed at higher taxonomic levels of organization, such as its ecological and functional stability, may in fact emerge at the level of strains.

While the SLM was able to sufficiently describe strain dynamics for the majority of strains across species, its success was not universal, and deviations from this typical pattern were also informative. In one host, for instance, two strains of *F. prausnitzii* appear to undergo rapid strain replacement and fail the test. Whether this replacement was due to shifting environmental conditions or direct interstrain competition is unclear. Regardless, our work indicates that any successful description of gut microbial dynamics must incorporate the possibilities of both coexistence and rapid replacement. Over very long timescales, in fact, strain replacement may dominate the stable dynamics that we observe here. Previous work (16, 28) suggests that over the course of decades, a large fraction of strains are ultimately replaced. One hypothesis is that this timescale reflects a waiting time for large environmental perturbations such as antibiotics (23, 41) or bowel cleanses (42), but this is just one of many hypotheses. Indeed, this hypothesis is partially challenged by Roodgar et al. (23), where the strain content of an adult gut was perturbed during a course of antibiotics but ultimately largely recovered to its pretreatment state. This antibiotic study is a powerful demonstration of the stability of strains even in the face of large perturbations. Investigating the possible explanations for the discrepancy between years-long and decades-long population dynamics at the strain level is an important problem that can be addressed with more extended timescales of observation.

While, in this work, we characterize the population dynamics of strains as ecological units, strains are by no means internally genetically homogeneous. The deep divergences that we detect between conspecific strains [$O(10^{3})$ to $O(10^{4})$ SNVs] are in fact genetic backgrounds, representing timescales of divergence likely far preceding the colonization of the host. However, individual lineages bearing these backgrounds can differ from one another both at sites in the core genome and in gene content, and the relative frequencies of these different lineages with respect to one another can change due to evolution. Previous studies have shown that at the level of lineages belonging to a strain, populations of gut microbes can experience both rapid selective sweeps (16, 31) and diversification into stably coexisting lineages (17).

How evolution impacts the ecological dynamics of strains and how, in turn, these ecological dynamics constrain and channel evolution are active areas of research (43). In the context of the SLM, these ecoevolutionary feedbacks can be viewed as tuning a strain’s carrying capacity, *K_i_*; growth rate, ${\text{τ}}_{i}^{-1}$; and sensitivity of the growth rate to environmental perturbation, σ*_i_*. Naively, it is expected that evolution would tend to increase the carrying capacity while minimizing the sensitivity of growth to abiotic fluctuations, but evolutionary modifications driving changes in one quantity may affect the other. The observed power-law scaling between the mean and variance in abundance (Taylor’s law) is, in essence, a constraint on *K_i_* given σ*_i_*, and vice versa. The SLM thus not only describes ecological dynamics but also, in conjunction with the empirical observation of macroecological laws, provides a useful framework for investigating the ecological effects of adaptation.

The SLM is ultimately a phenomenological model, not a mechanistic one, and its success at the strain level does not explain why strains coexist. How and why closely related strains coexist in the human gut are two of the central biological questions raised by our results. Spatial segregation between strains, perhaps occupying different colonic crypts, or partitioning luminal and mucosal niches, could contribute to the observed pattern of strain coexistence (44–46), much as it does among Cutibacterium acnes strains inhabiting different pores on the facial microbiome (25). However, the spatial structure is far from the only mechanism that can foster coexistence between strains. For instance, differences in genetic content at polysaccharide utilization loci may contribute to intrahost metabolic niche differentiation, potentially favoring the coexistence of closely related strains (47). Moreover, stably coexisting strains have been reported under laboratory conditions as well (26, 48). In these experiments, strains may coexist by finely partitioning some aspect of the abiotic environment, by engaging in ecological interactions (e.g., cross-feeding), or by some combination of both. Indeed, recent theoretical work suggests that even subtle differences in resource uptake rates under high- and low-nutrient conditions may, in the presence of a temporally variable environment, lead to the coexistence of small numbers of closely related strains (49). Investigating which of these mechanisms promotes strain coexistence in the human gut microbiome and identifying the relevant genomic architectures are important avenues for future research.

We note that the four hosts examined here are not a representative sample of the full diversity of human lifestyles. For instance, all hosts were between the ages of 21 and 37 years and resided in the United States at the time of sampling. The proportion of strains exhibiting stable or unstable dynamics may vary in different cohorts, but the tests that we developed here will nonetheless be useful in identifying such differences. A future avenue of work will be to assess the generality of these findings in different cohorts, using new time series, which are at least as long and densely temporally sampled as the BIO-ML data analyzed here and are of a similar quality, including diseased or perturbed cohorts, which may exhibit quite different dynamics.

Finally, our work highlights the importance of strains in understanding community structure and dynamics in the human gut microbiome (26, 49). The ambiguity surrounding the bacterial species concept is well known (50), and reasonable alternatives have been proposed (51), but operationally, species are nonetheless the predominant focus of attention in gut microbiome ecology. This focus is reasonable, as within-host strain structure is a comparatively recent discovery (16, 24, 29), and 16S rRNA gene sequencing provides an inexpensive, high-throughput means to examine community dynamics. However, it is reasonable to propose that for the human gut, and perhaps other microbial ecosystems, many higher-level macroecological patterns of abundance and diversity may originate at the level of strains.

## MATERIALS AND METHODS

### Longitudinal data.

To investigate the temporal dynamics of the human gut microbiome, we analyzed densely sampled shotgun metagenomic time series data from four hosts from the BIO-ML project (accession number: PRJNA544527) (31). By analyzing shotgun metagenomic sequences, we capture longitudinal patterns of intraspecific genetic variation for many bacterial species in these communities simultaneously. A total of 402 samples were drawn from these four individuals (hosts *am*, *ao*, *an*, and *ae*), with 206 samples coming from host *am*, 74 from *ao*, 63 from *an*, and 59 from *ae* (see Table S1 and Fig. S2 in Text S1 in the supplemental material for further details on sampling). All four hosts were healthy adults between the ages of 21 and 37 years, three of whom were male and one of whom was female, and all were residing in the United States at the time of sampling (see Data Set S1 for further details). Crucially, for our purposes, these hosts were sampled at a very fine temporal resolution, with a median interval between successive samples of either 1 or 2 days in each host, over a period of 5 months (host *ao*) to 18 months (host *am*).

### Aligning reads.

To call single nucleotide variants (SNVs) and gene content, we aligned shotgun metagenomic reads to a panel of species that were prevalent and abundant within each host using MIDAS (52) (see Text S1, section 2, for further details on the bioinformatic pipeline employed). In total, we detected 45 species across the four hosts that met our coverage and prevalence criteria. To reflect a more recently published taxonomy (53), the names of three species (*Phocaeicola vulgatus*, *Phocaeicola massiliensis*, and *Lachnospira eligens*) were amended from the names of these species native to MIDAS (Bacteroides vulgatus, Bacteroides massiliensis, and Eubacterium eligens).

### Stationarity of intraspecific genetic variation.

To calculate *F_ST_*, a measure of subpopulation differentiation, we used the estimator:

(4) $$\begin{array}{l} F_{\mathrm{ST}}=\frac{{\text{π}}_{\mathrm{BT}}-\text{π}}{{\text{π}}_{\mathrm{BT}}} \end{array}$$

where π is the nucleotide diversity within a population and π*_BT_* is the level of diversity between populations.

The nucleotide diversity, π, is a classical population genetic measure of polymorphism, representing the average number of pairwise SNV differences between randomly chosen members of a population. To determine π for a given species within a sample, we used the estimator:

(5) $$\begin{array}{l} \text{π}=\frac{1}{\left|G\right|}\sum_{i=1}^{\left|G\right|}\frac{r_{i}}{d_{i}}\frac{a_{i}}{d_{i}-1}+\frac{a_{i}}{d_{i}}\frac{r_{i}}{d_{i}-1} \end{array}$$

where *r_i_* is the count of the reference allele at site *i*, *a_i_* is the count of the alternate allele, *d_i_* is the depth of coverage, and |*G*| is the total number of sites in the genome. This quantity was calculated after first excluding sites with low read depths (<5×), as reliable estimates of true allele frequency cannot be made for such sites. Our π calculations follow the same methodology as the one described previously by Schloissnig et al. (11) in an early, foundational work characterizing patterns of genetic diversity in gut microbial populations within and across hosts and are meant to be directly comparable.

Similarly, π*_BT_*, the diversity between time points, was calculated as:

(6) $$\begin{array}{l} {\text{π}}_{\mathrm{BT}}=\frac{1}{\left|G\right|}\sum_{i=1}^{\left|G\right|}\left(\frac{r_{t_{1}i}}{d_{t_{1}i}}\frac{a_{t_{2}i}}{d_{t_{2}i}}+\frac{a_{t_{1}i}}{d_{t_{1}i}}\frac{r_{t_{2}i}}{d_{t_{2}i}}\right) \end{array}$$

where *r_ji_*, *a_ji_*, and *d_ji_* are the reference allele count, alternate allele count, and depth of coverage of site *i* in sample *j*, respectively, and |*G*| is the total number of sites in the genome.

We calculated π for each species in each sample in each host and π*_BT_* for each sample relative to the initial time point.

To calculate the *F_ST_* between the initial time point (sample *i*) and sample *j*, we used the formula:

(7) $$\begin{array}{l} F_{\mathrm{ST}}^{\left(\mathrm{ij}\right)}=\frac{{\text{π}}_{\mathrm{BT}}-\frac{\left({\text{π}}_{i}+{\text{π}}_{j}\right)}{2}}{{\text{π}}_{\mathrm{BT}}} \end{array}$$

Finally, we obtained our normalized statistic $F_{\mathrm{ST}}^{\prime }$ by dividing $F_{\mathrm{ST}}^{\left(\mathrm{ij}\right)}$ by the species mean interhost *F_ST_*, estimated from a panel of 250 North American subjects sequenced in the Human Microbiome Project (6, 38). To determine this species mean *F_ST_*, we first calculated the pairwise *F_ST_* for each pair of samples in which a species appeared and then took the mean of these values.

To implement the augmented Dickey-Fuller test on each time series of $F_{\mathrm{ST}}^{\prime }$ values, we used the adfuller function from the python statsmodels library (54).

### Strain inference.

To phase strains, we use a modified version of the allele frequency trajectory clustering algorithm developed by Roodgar et al. (23). While the approach of Roodgar et al. was appropriate for their purposes, namely, detecting selective sweeps of linked variants that deviated substantially from the overall background, our clustering scheme is designed to detect only large clusters of SNVs (minimally >1,000 SNVs within a cluster) whose linkage patterns are consistent with perfect linkage on a single haplotype background. Our choice of 1,000 SNVs as a cutoff was informed by previous work estimating the typical scale of genetic divergence between strains found in different hosts (see Text S1, section 3.1, for further information). While lineages can and do diverge as a result of diversifying evolution within hosts (17, 31), by imposing a minimum cluster size of 1,000 core-genome SNVs, we expect largely to exclude cases of within-host diversification. When no cluster of >1,000 SNVs was detected, only a single strain was inferred to be present.

### Macroecology.

To fit Taylor’s law (Fig. 3A), we used the polyfit function from the python numpy library to fit a power-law regression between the mean and the variance of each strain’s abundance distribution. To obtain the log-rescaled gamma AFD (Fig. 3B), each strain’s abundance distribution was first log rescaled and then normalized to have zero mean and unit variance. We then binned each strain’s rescaled abundance distribution into 20 evenly spaced bins and fit the gamma AFD to the bin-wise mean across strains. To perform the gamma AFD fit, we adapted code from J. Grilli (32).

### Data availability.

All necessary metadata, as well as the source code for the MIDAS metagenomic pipeline, downstream analyses, and figures, are available on GitHub at https://github.com/garudlab/StrainStability.
