# Supplementary material for: Ecological Stability Emerges at the Level of Strains in the Human Gut Microbiome
Source: mBio. 2023 Feb 21;14(2):e02502-22. doi: 10.1128/mbio.02502-22 (PMC10127601; doi:10.1128/mbio.02502-22)
Supplement: TEXT S2 [file mbio.02502-22-s0002.pdf]

S2 Text

F_{ST}', strain frequency, and strain abundance dynamics plots for all species analyzed, for host *am.* These plots are analogous to Main Text Figure 1. When only a single strain was detected, only F_{ST}' and strain abundance dynamics plots, but no strain frequency plot, is included.

Table of Contents

Bacteroides fragilis A	*1*
Bacteroides ovatus A	*2*
Bacteroides ovatus B	*3*
Bacteroides stercoris A	*4*
Bacteroides uniformis A	*5*
Phocaeicola vulgatus A	*6*
Phocaeicola vulgatus B	*7*
Phocaeicola vulgatus C	*8*
Bacteroides xylanisolvens A	*9*
Bacteroides xylanisolvens B	*10*
Barnesiella intenstinihominis A	*11*
Eubacterium rectale A	*12*
Eubacterium rectale B	*13*
Faecalibacterium prausnitzii (57453) A	*14*
Phascolarctobacterium sp. A	*15*
Parabacteroides merdae A	*16*
Ruminoccocus bicirculans A	*17*
Ruminoccocus bromii A	*18*

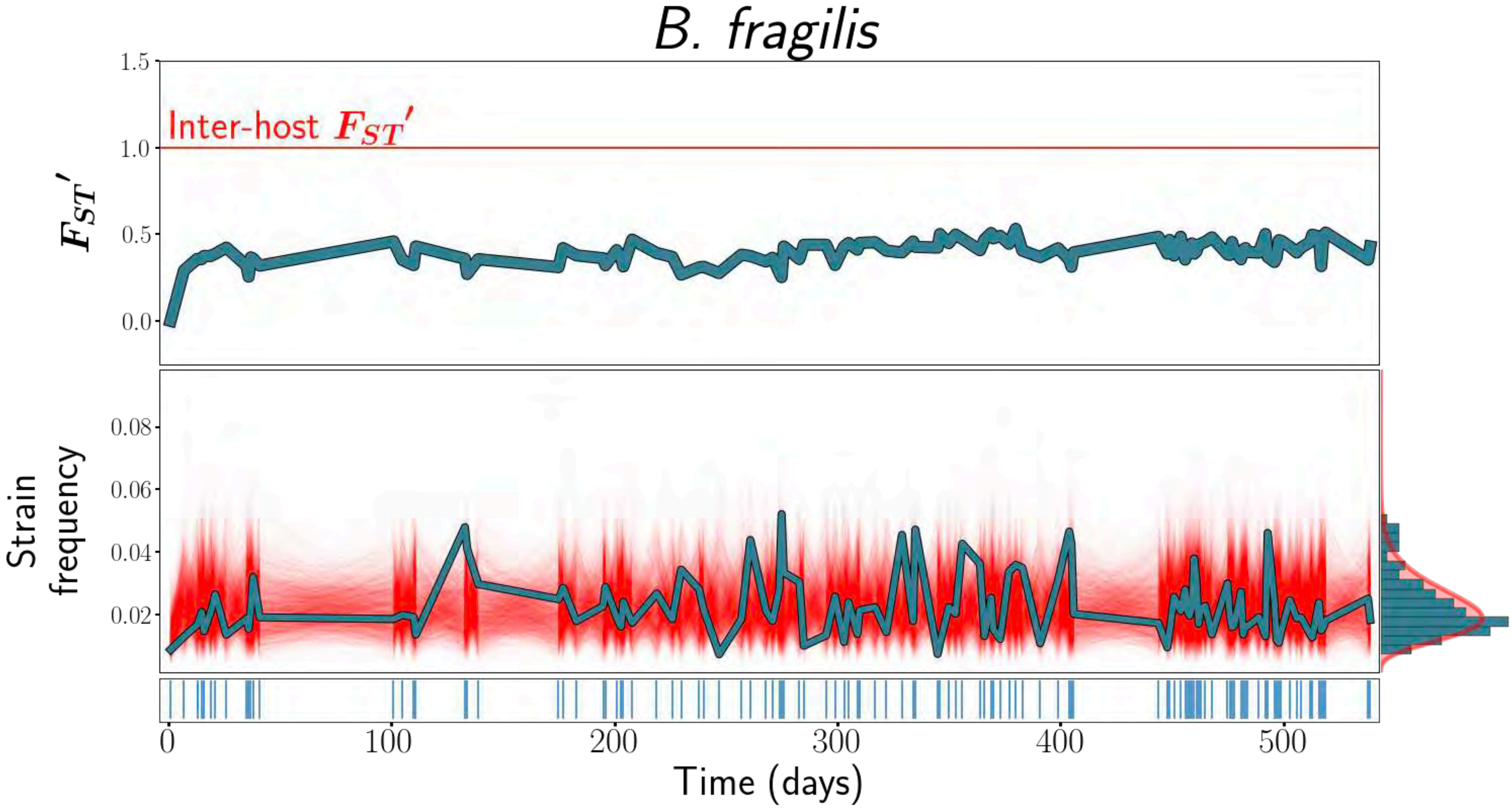

B. fragilis
Inter-host F_{ST}'
F_{ST}'
1.5
1.0
0.5
0.0
Strain frequency
0.08
0.06
0.04
0.02
0
100
200
300
400
500
Time (days)

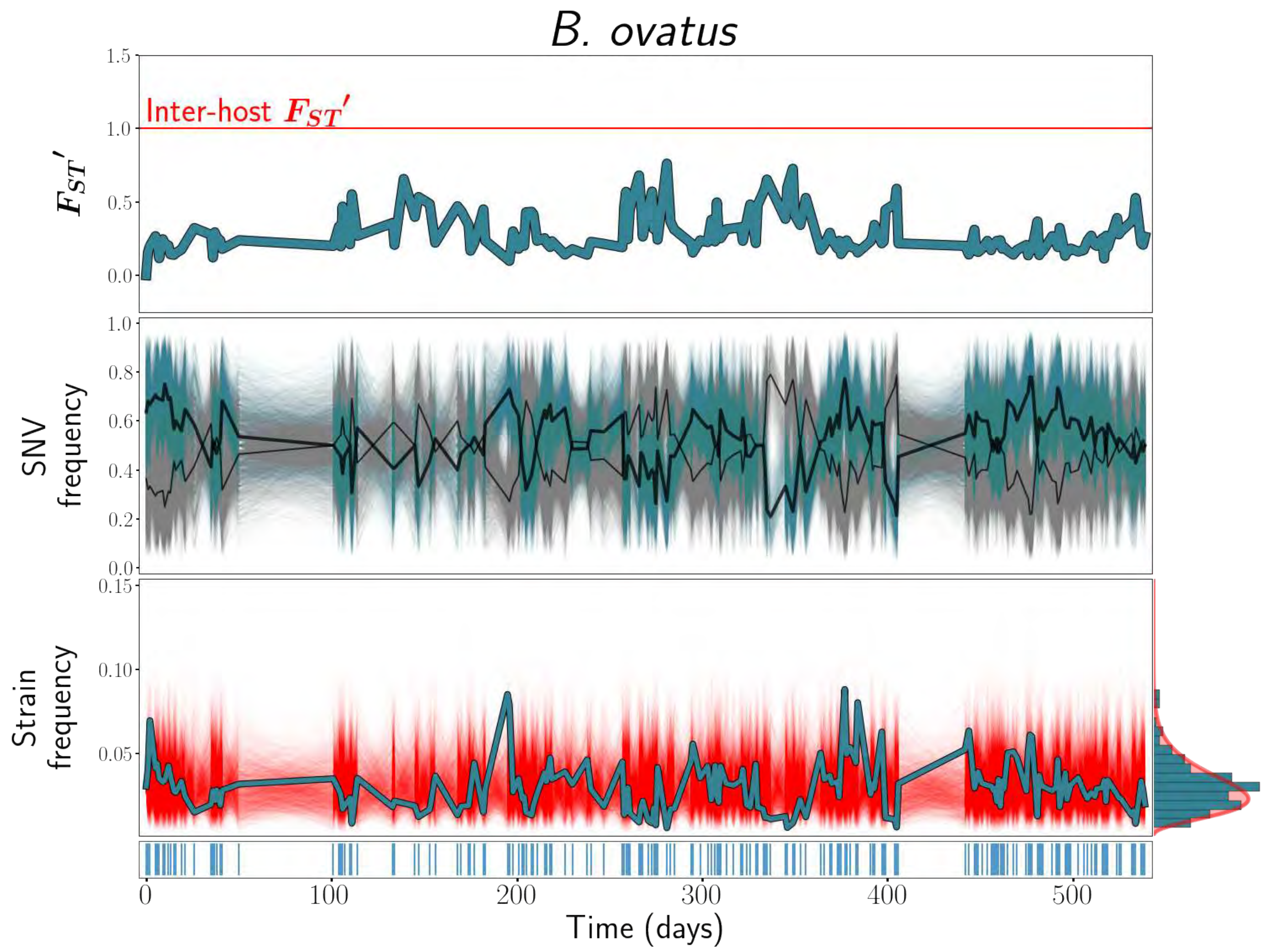

B. ovatus
Inter-host F_{ST}'
F_{ST}'
1.5
1.0
0.5
0.0
SNV frequency
1.0
0.8
0.6
0.4
0.2
0.0
Strain frequency
0.15
0.10
0.05
0
100
200
300
400
500
Time (days)

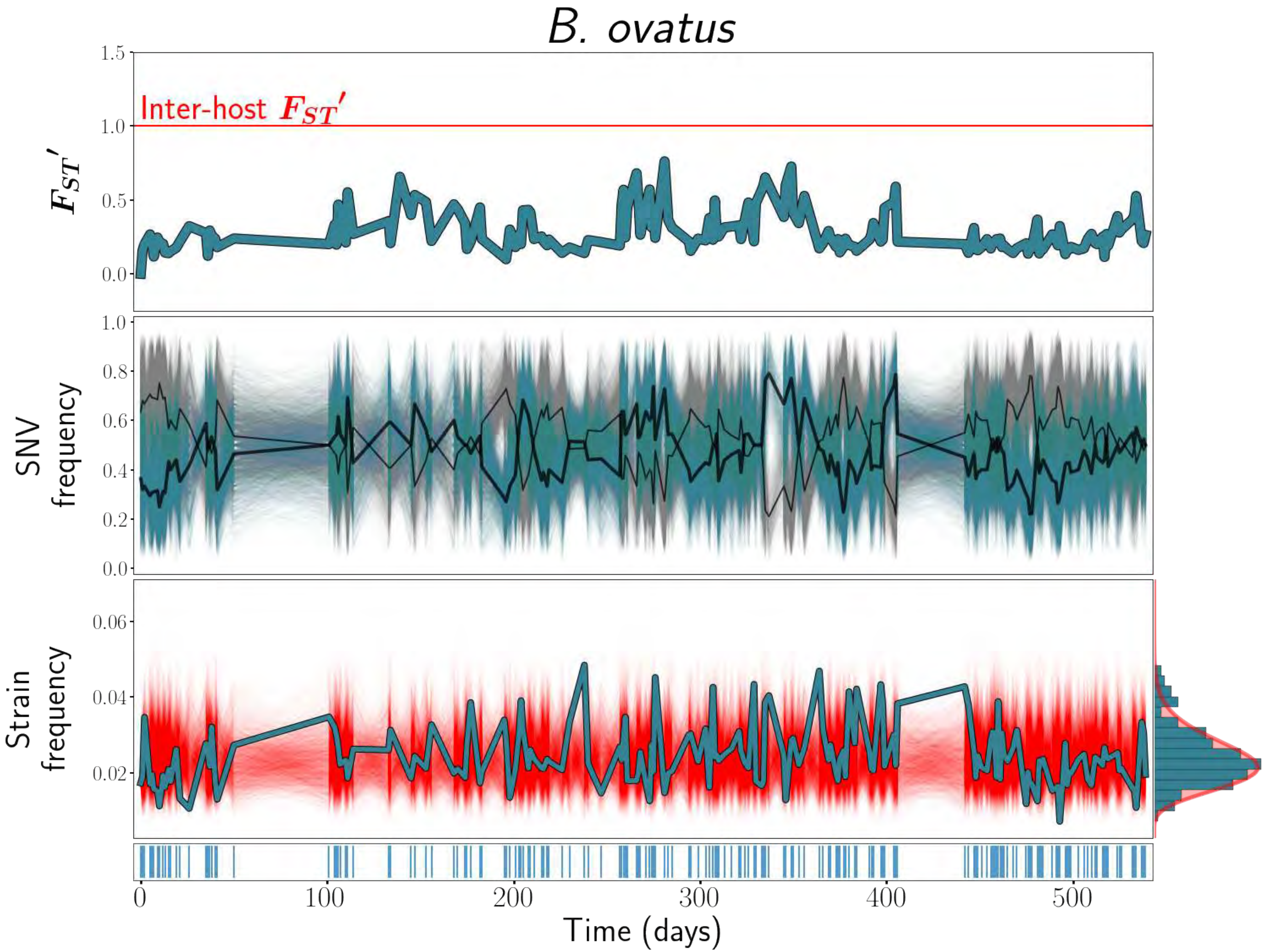

B. ovatus
Inter-host F_{ST}'
F_{ST}'
1.5
1.0
0.5
0.0
SNV frequency
1.0
0.8
0.6
0.4
0.2
0.0
Strain frequency
0.06
0.04
0.02
0
100
200
300
400
500
Time (days)

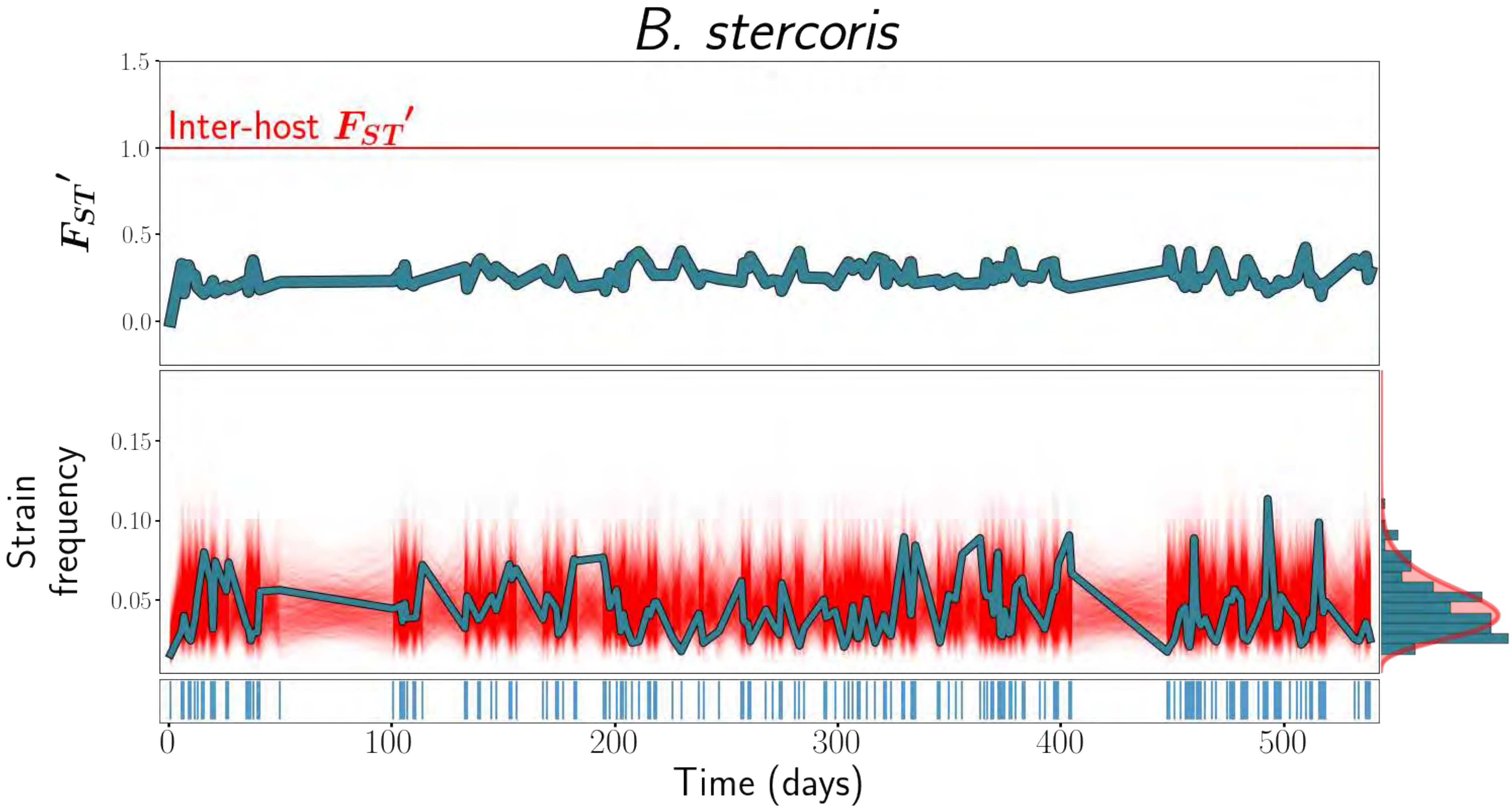

B. stercoris
Inter-host F_{ST}'
F_{ST}'
1.5
1.0
0.5
0.0
Strain frequency
0.15
0.10
0.05
0
100
200
300
400
500
Time (days)

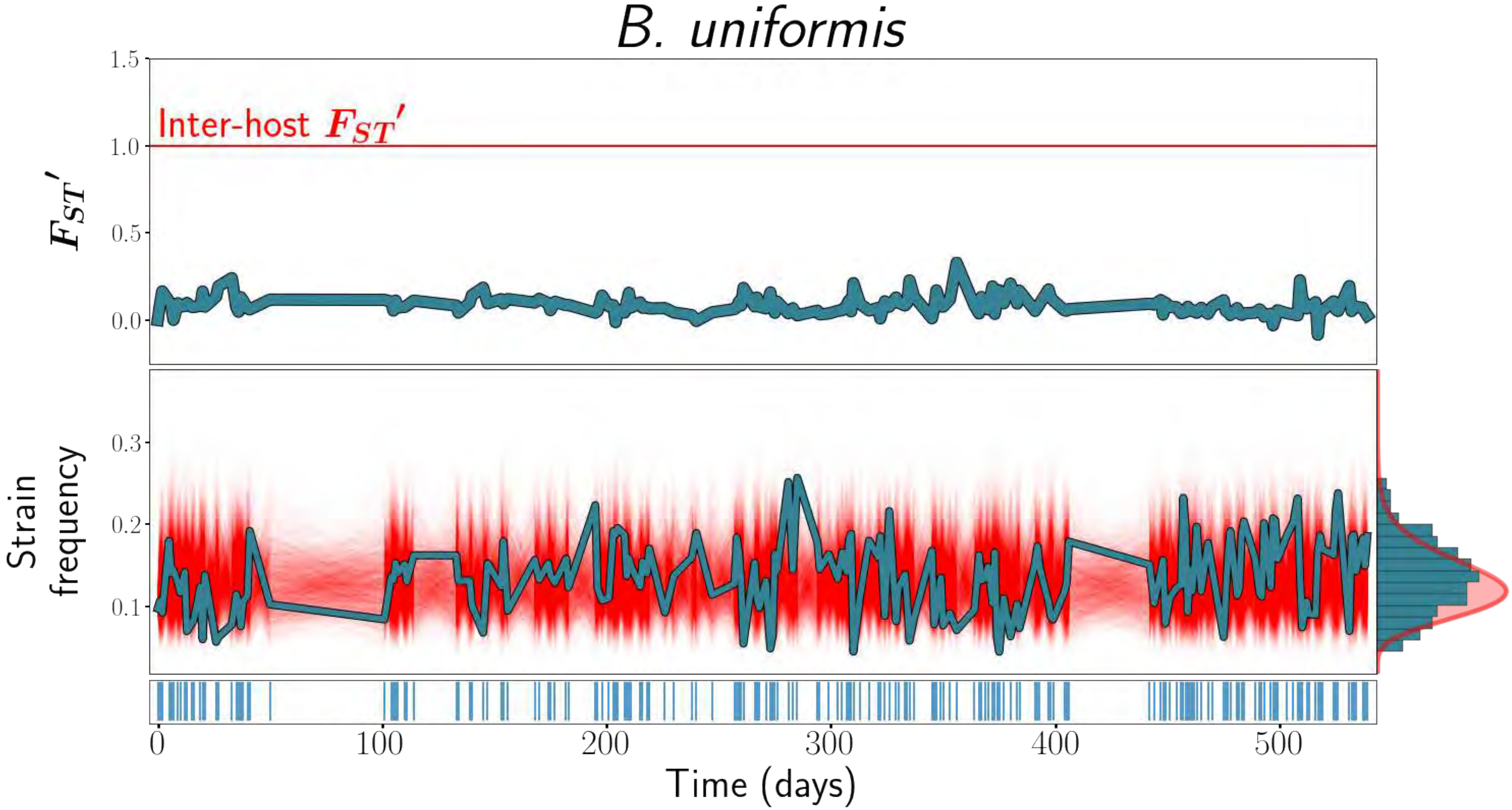

B. uniformis
Inter-host F_{ST}'
F_{ST}'
1.5
1.0
0.5
0.0
Strain frequency
0.3
0.2
0.1
0
100
200
300
400
500
Time (days)

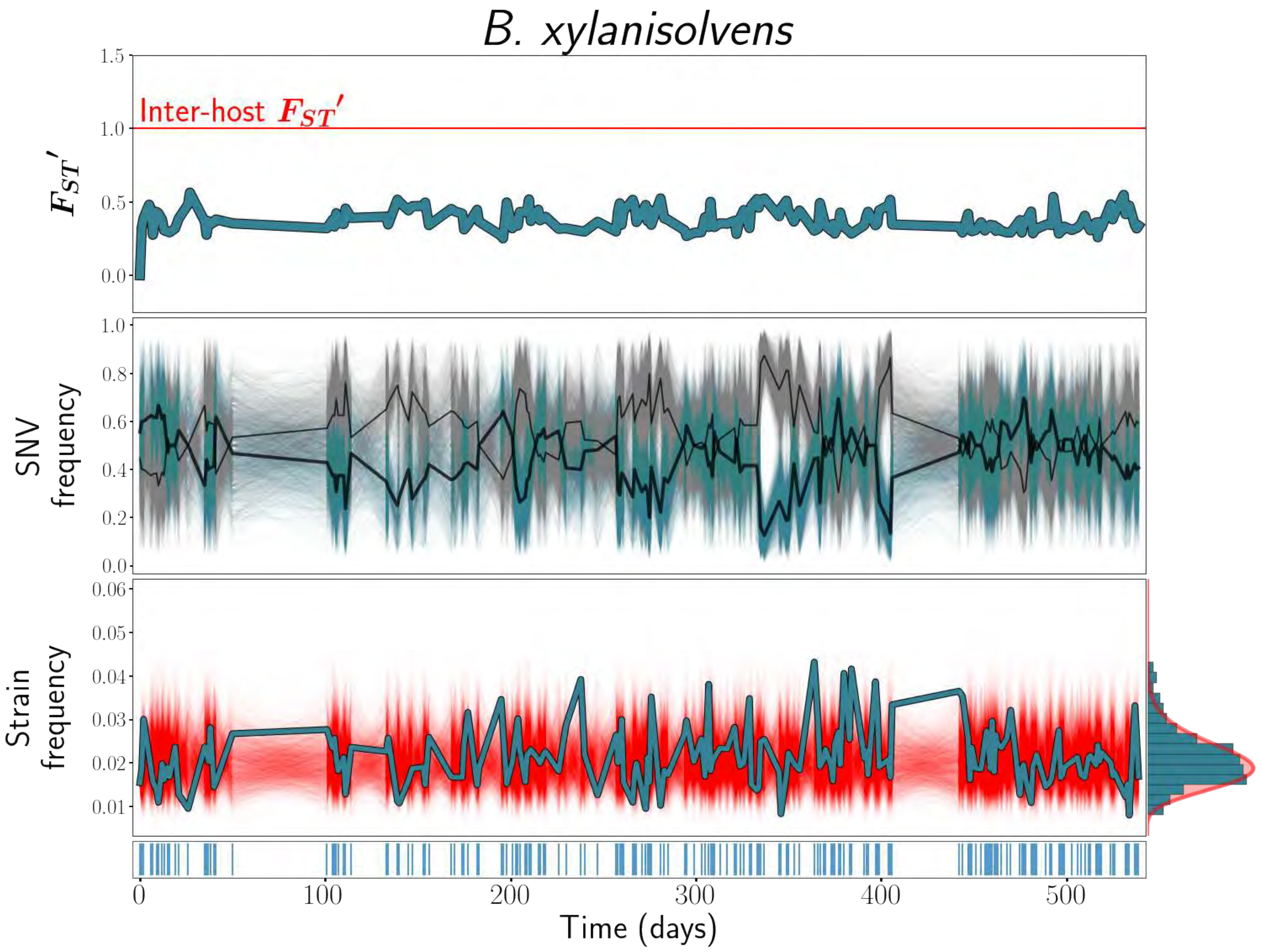
B. xylanisolvens
Inter-host F_{ST}'
F_{ST}'
SNV frequency
Strain frequency
Time (days)
1.5
1.0
0.5
0.0
0.8
0.6
0.4
0.2
0.06
0.05
0.04
0.03
0.02
0.01
0
100
200
300
400
500

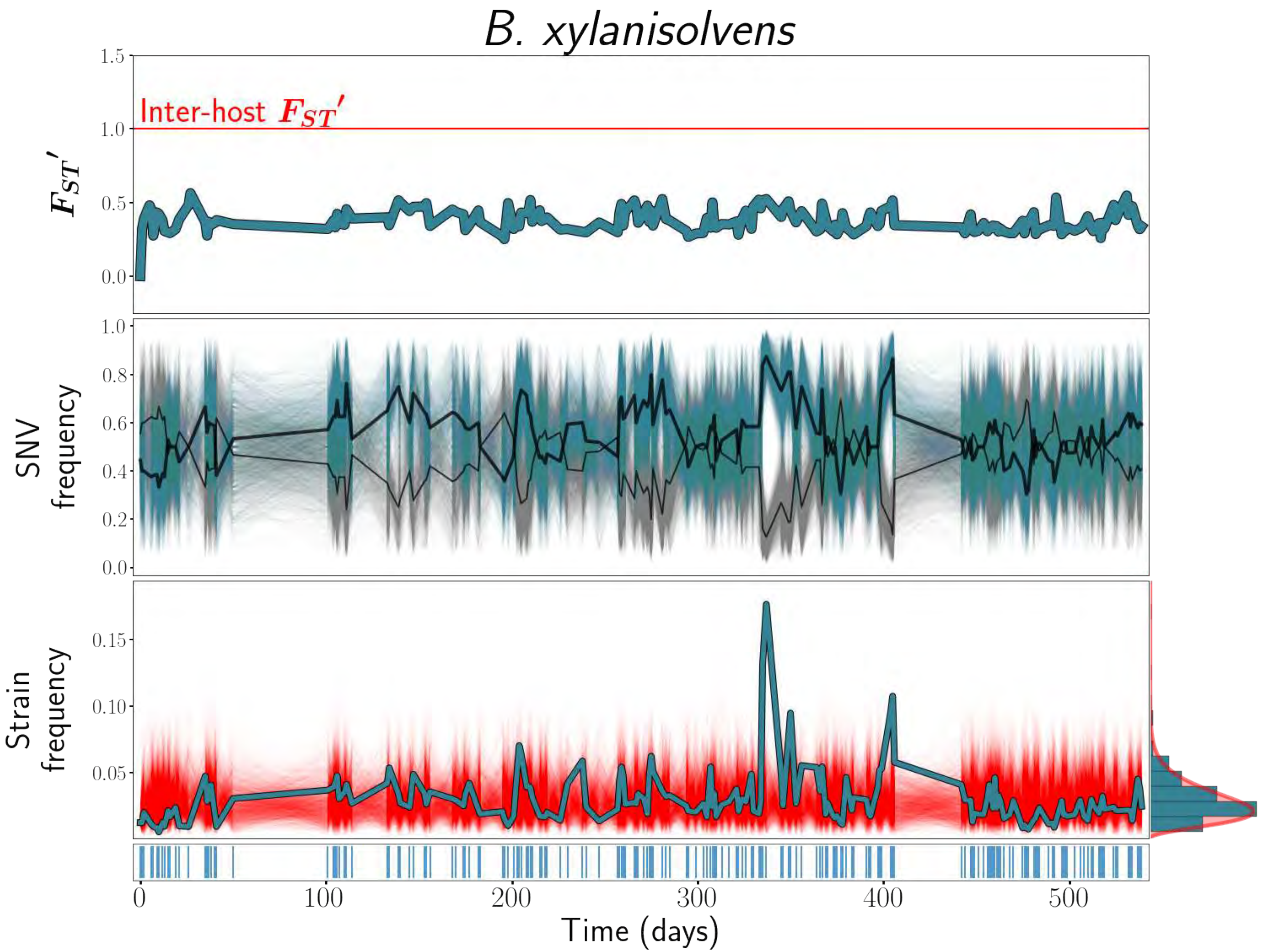

B. xylanisolvens
Inter-host F_{ST}'
F_{ST}'
SNV frequency
Strain frequency
Time (days)

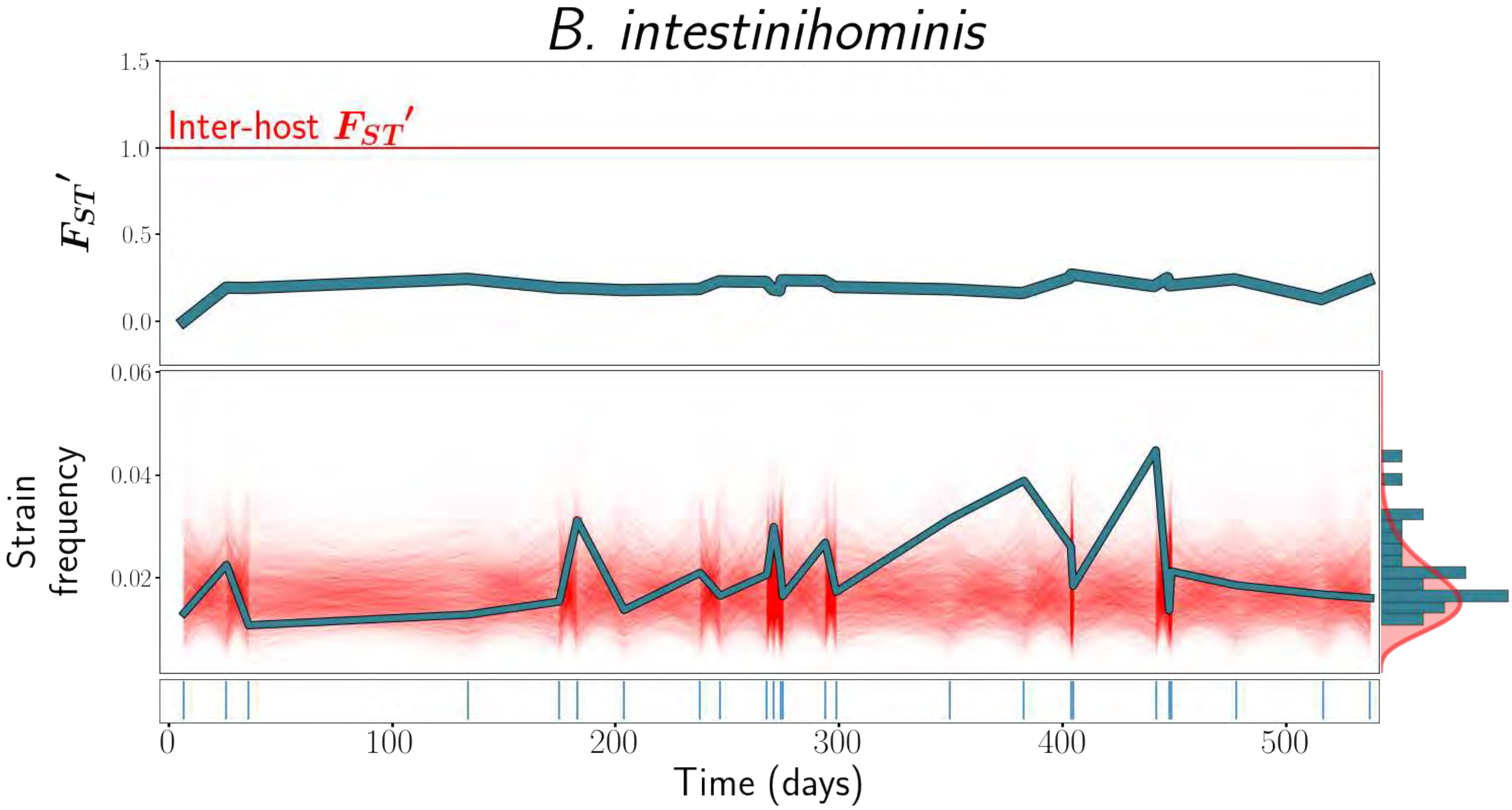

B. intestinihominis
Inter-host F_{ST}'
F_{ST}'
1.5
1.0
0.5
0.0
Strain frequency
0.06
0.04
0.02
0
100
200
300
400
500
Time (days)

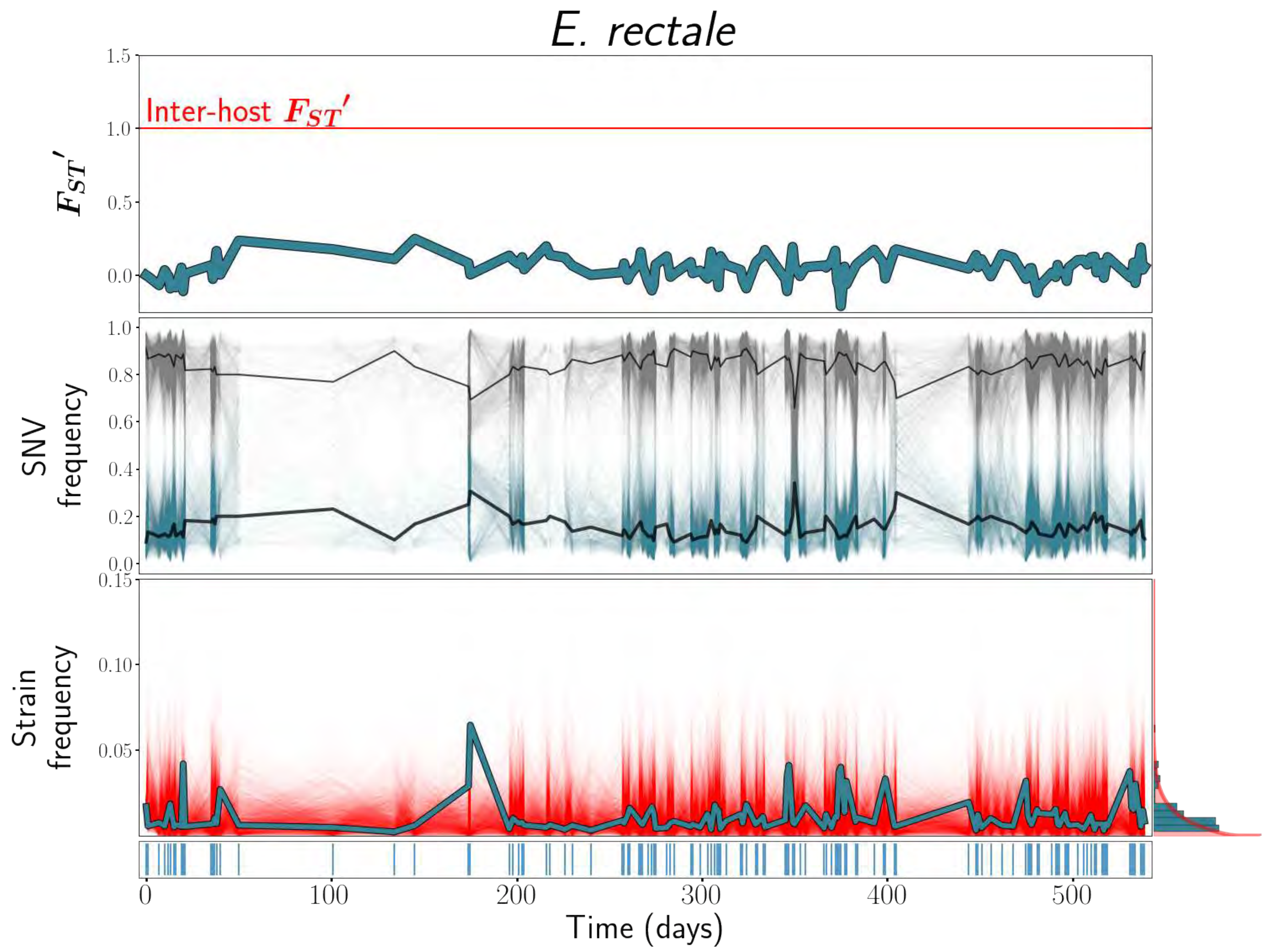

E. rectale
Inter-host F_{ST}'
F_{ST}'
SNV frequency
Strain frequency
Time (days)
1.5
1.0
0.5
0.0
0.8
0.6
0.4
0.2
0.15
0.10
0.05
0
100
200
300
400
500

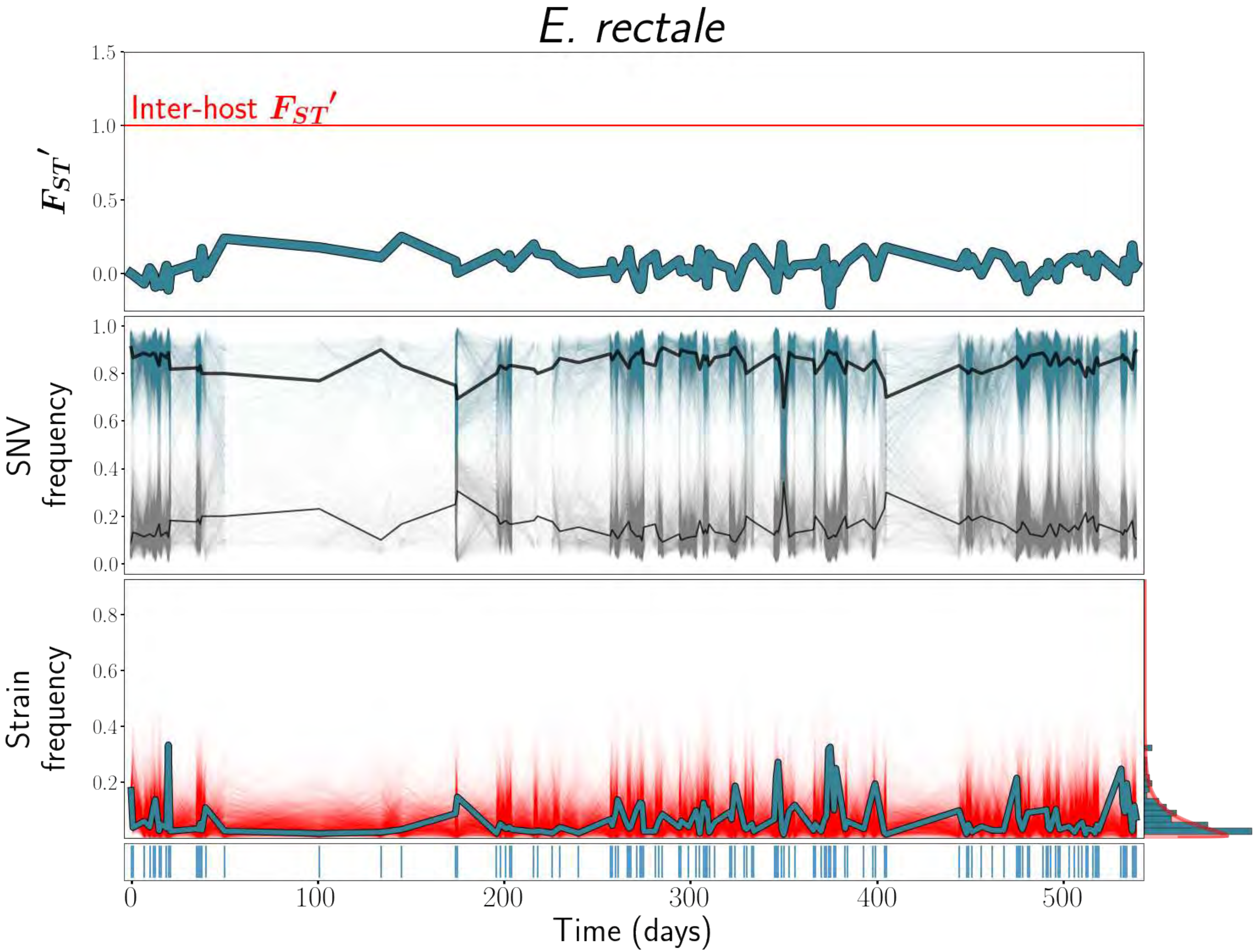

E. rectale
Inter-host F_{ST}'
F_{ST}'
SNV frequency
Strain frequency
Time (days)

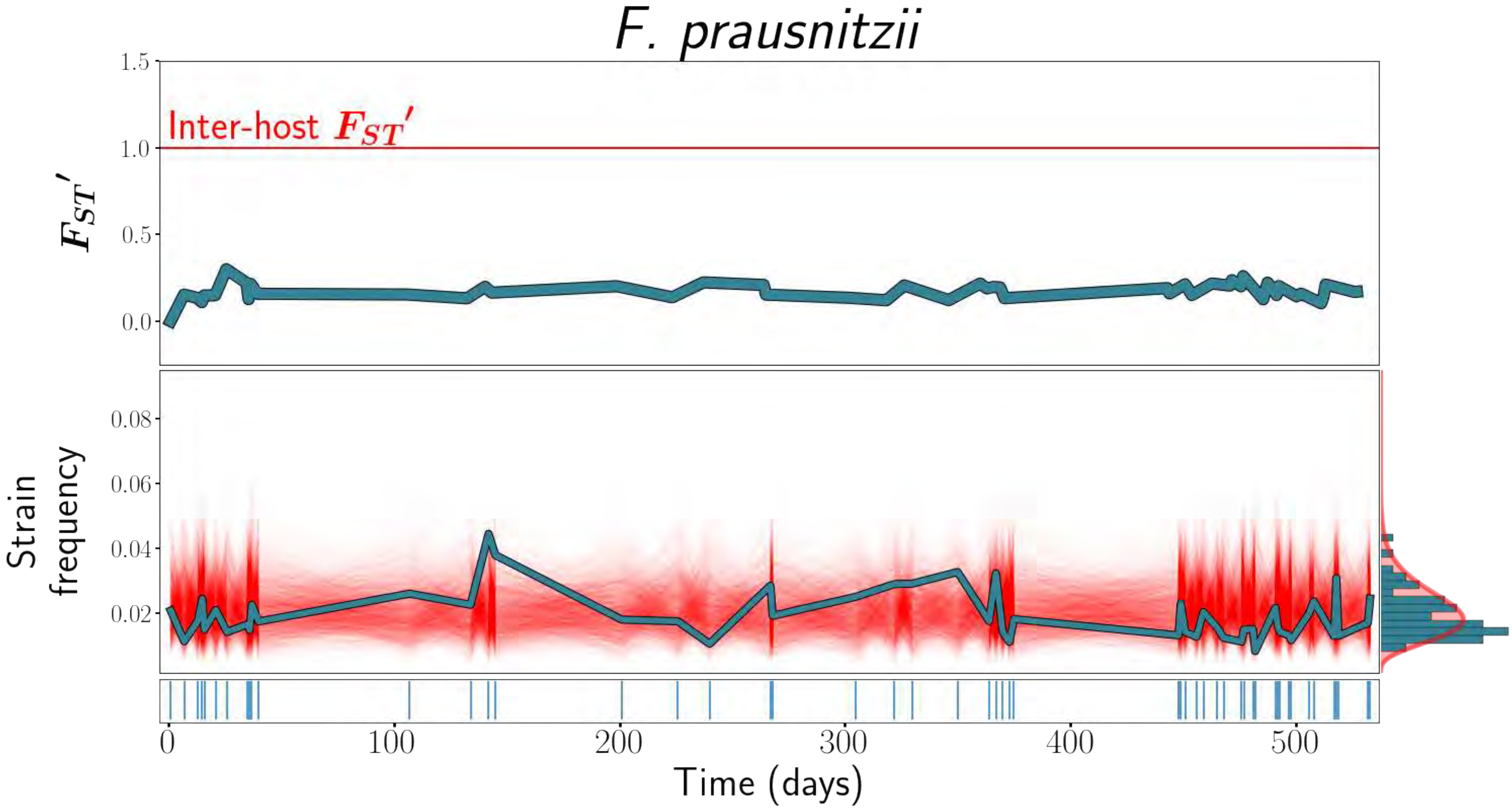
F. prausnitzii
Inter-host F_{ST}'
F_{ST}'
1.5
1.0
0.5
0.0
Strain frequency
0.08
0.06
0.04
0.02
0
100
200
300
400
500
Time (days)

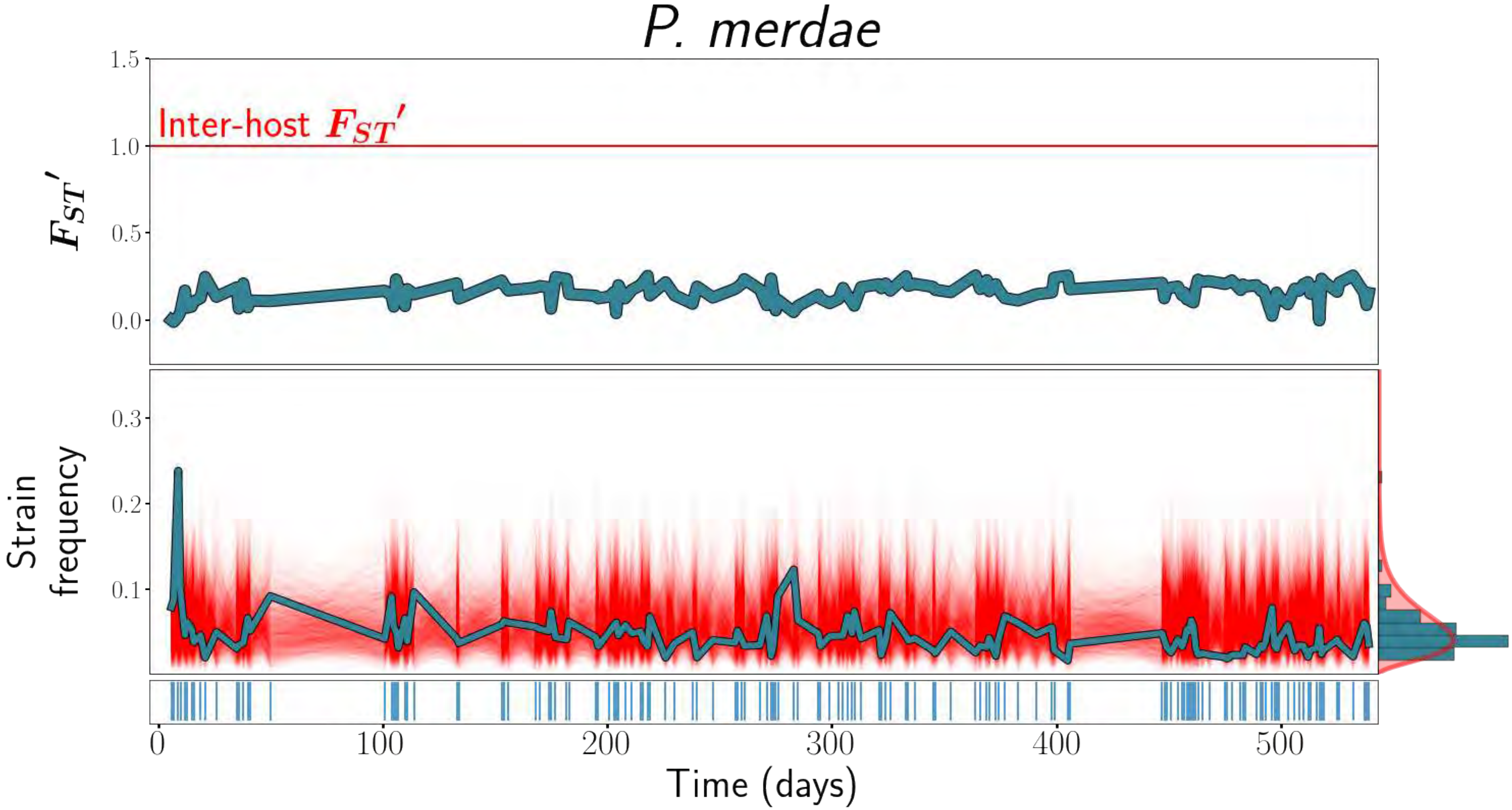

P. merdae
Inter-host F_{ST}'
F_{ST}'
1.5
1.0
0.5
0.0
Strain frequency
0.3
0.2
0.1
0
100
200
300
400
500
Time (days)

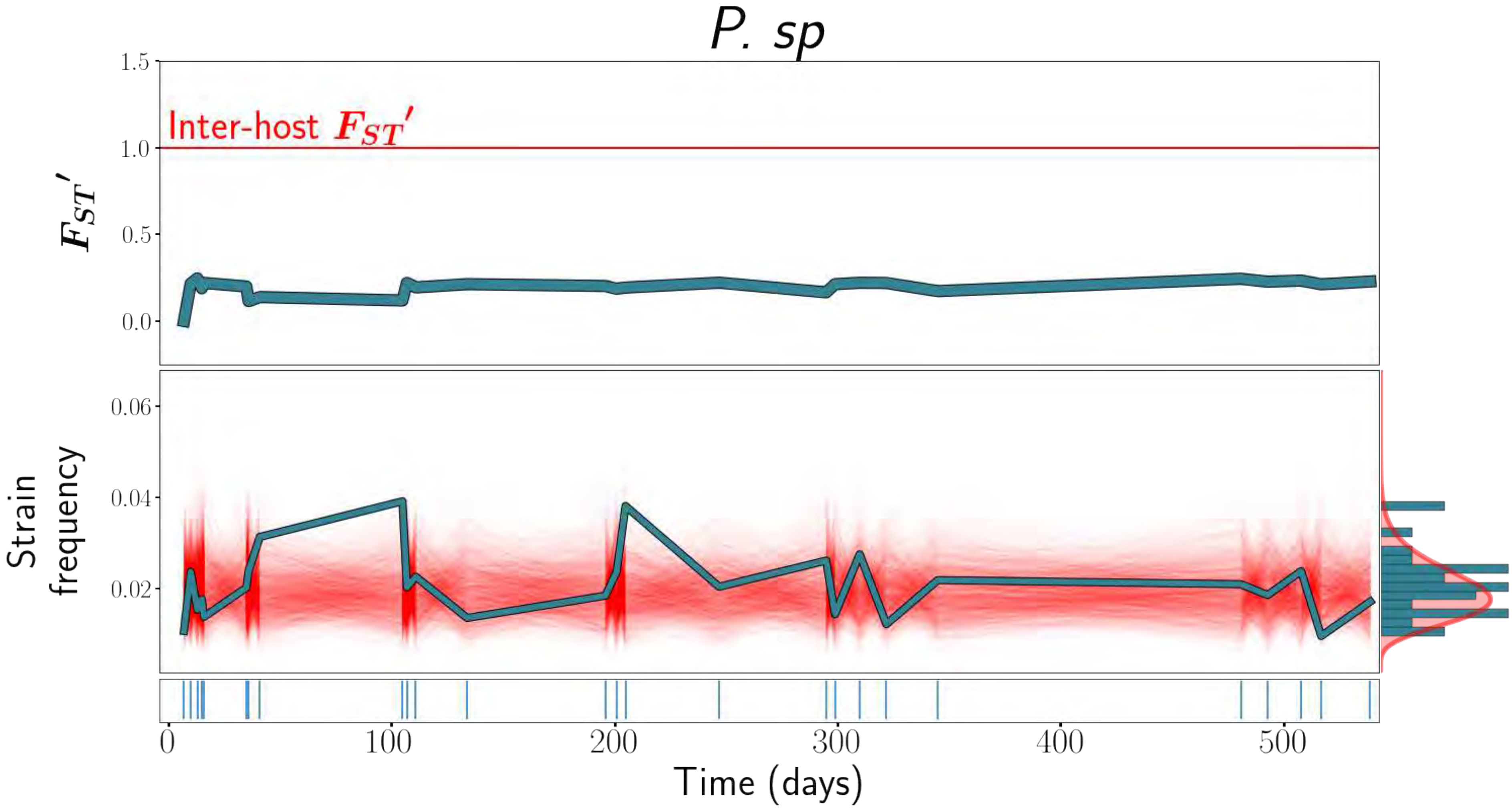

P. sp
Inter-host F_{ST}'
F_{ST}'
1.5
1.0
0.5
0.0
Strain frequency
0.06
0.04
0.02
0
100
200
300
400
500
Time (days)

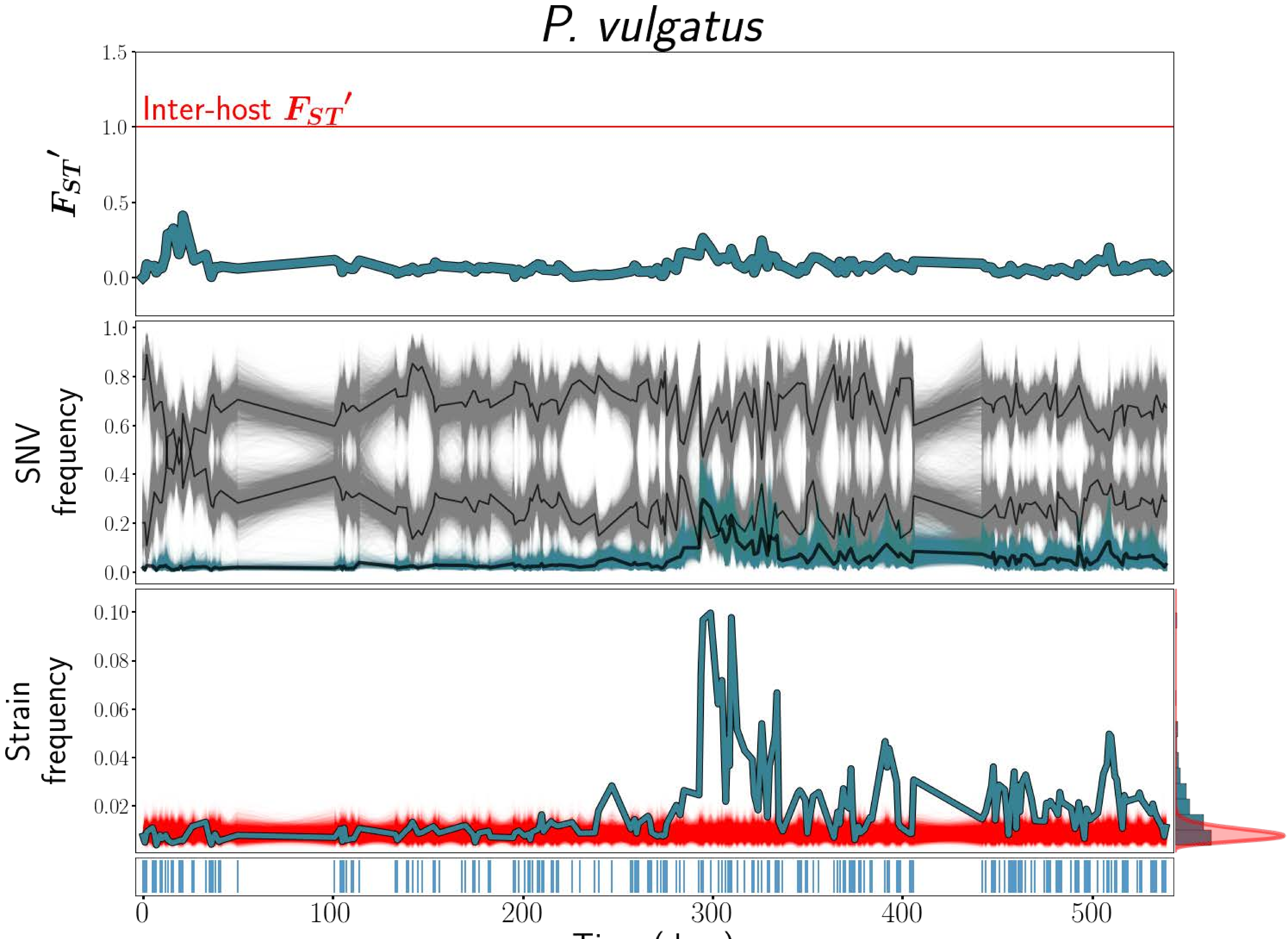
P. vulgatus
Inter-host F_{ST}'
F_{ST}'
SNV frequency
Strain frequency
Time (days)
0
100
200
300
400
500
0.0
0.5
1.0
1.5
0.2
0.4
0.6
0.8
0.02
0.04
0.06
0.08
0.10

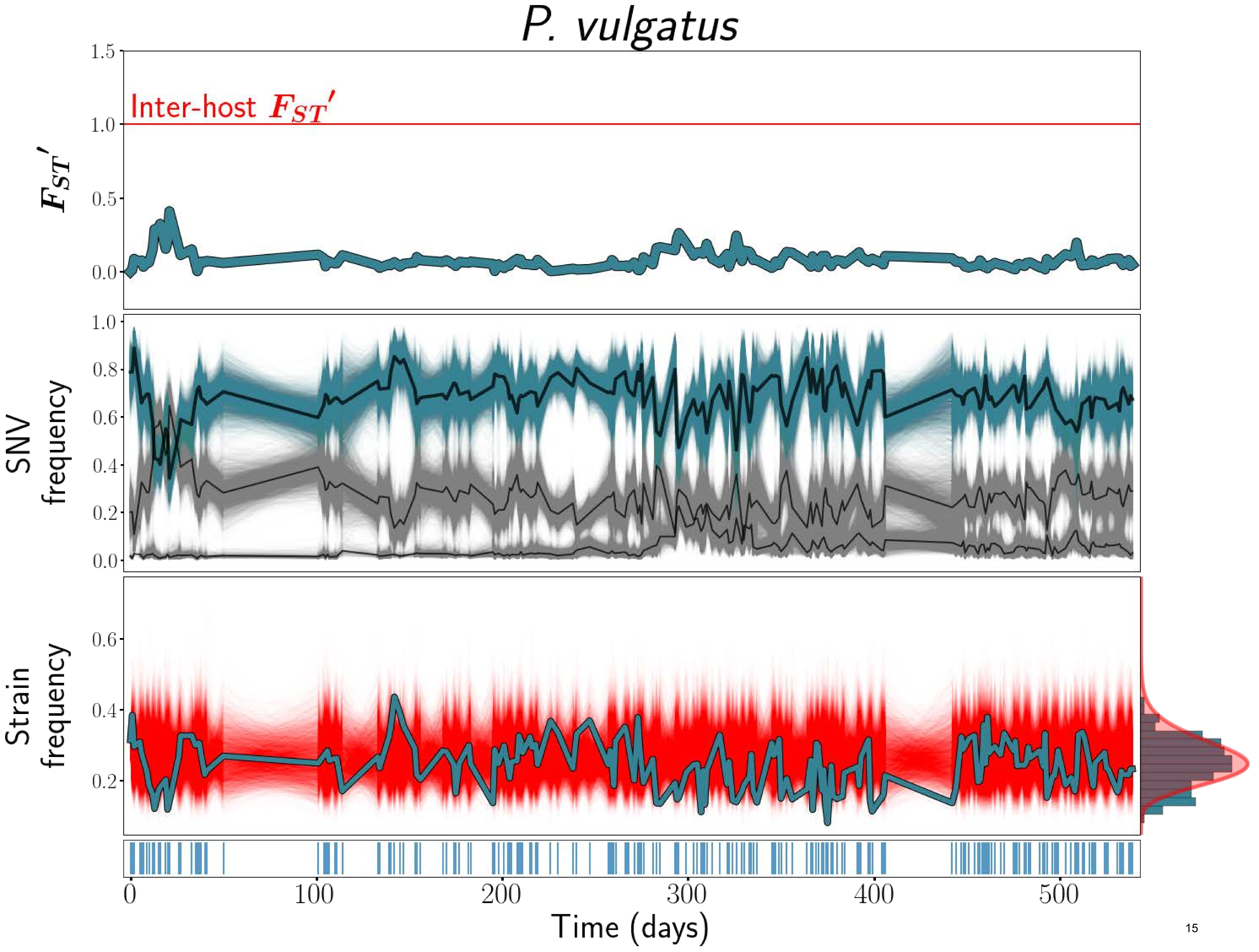

P. vulgatus
Inter-host F_{ST}'
F_{ST}'
SNV frequency
Strain frequency
Time (days)

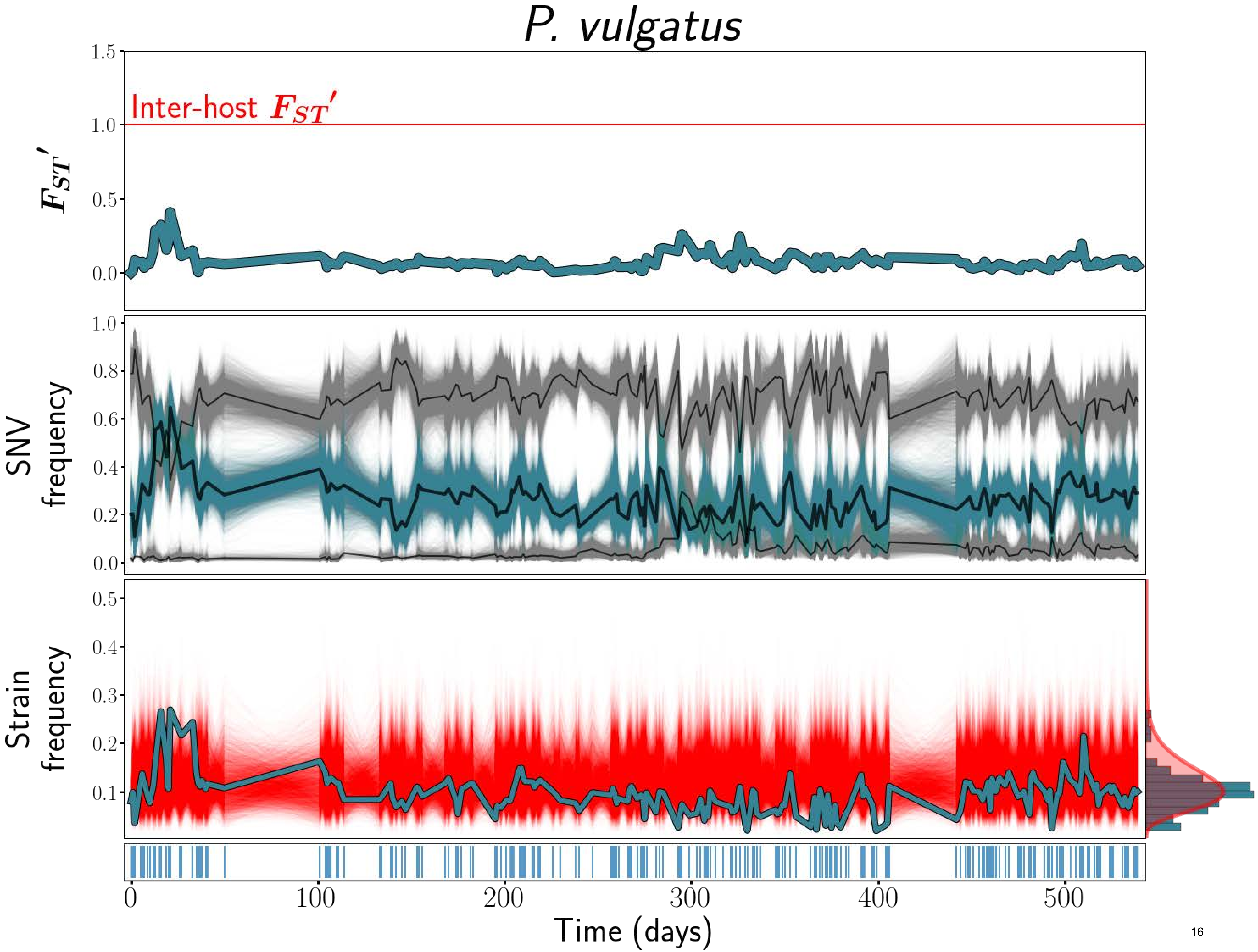

P. vulgatus
Inter-host F_{ST}'
F_{ST}'
SNV frequency
Strain frequency
Time (days)

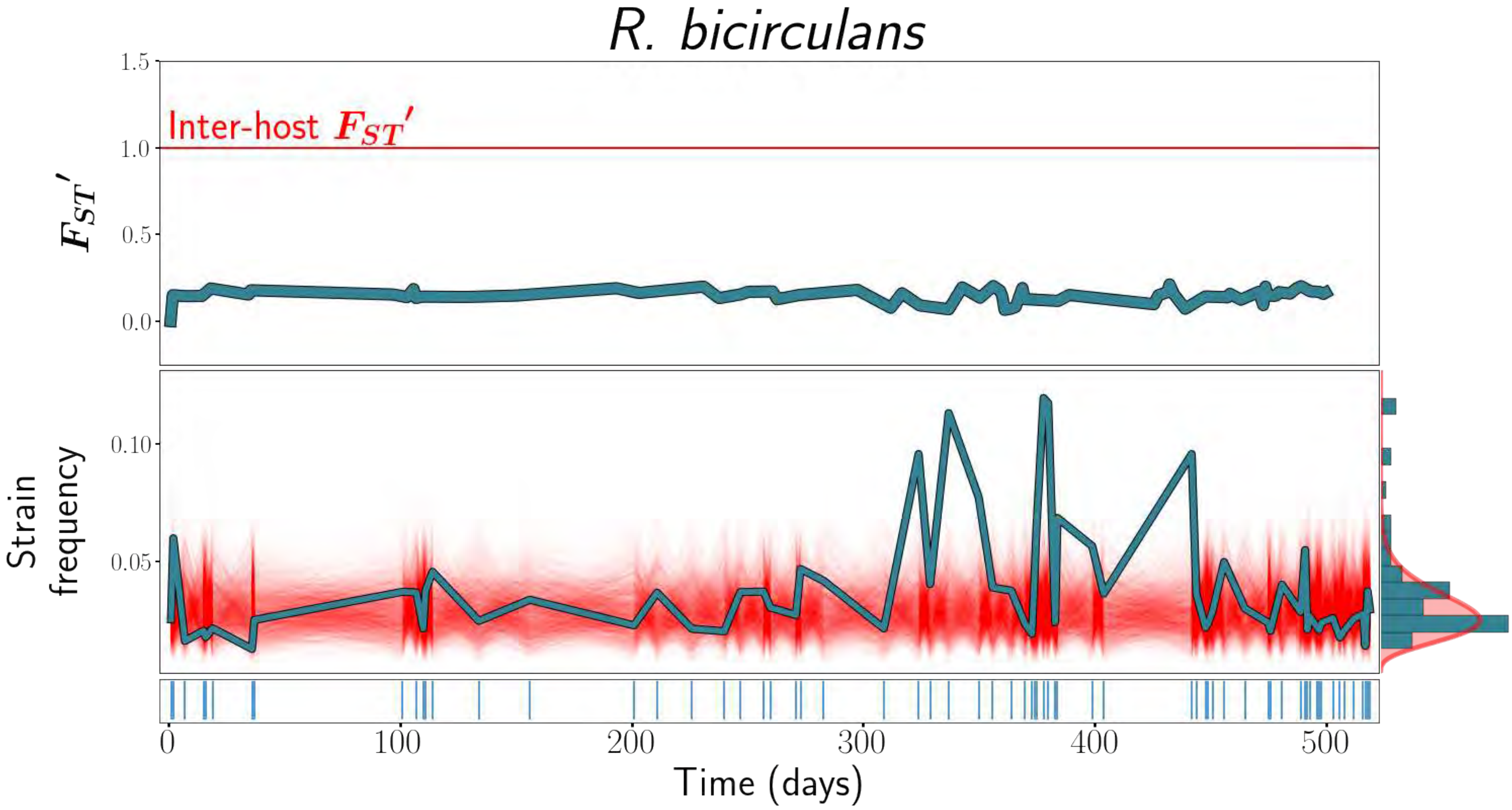
R. bicirculans
Inter-host F_{ST}'
F_{ST}'
1.5
1.0
0.5
0.0
Strain frequency
0.10
0.05
0
100
200
300
400
500
Time (days)

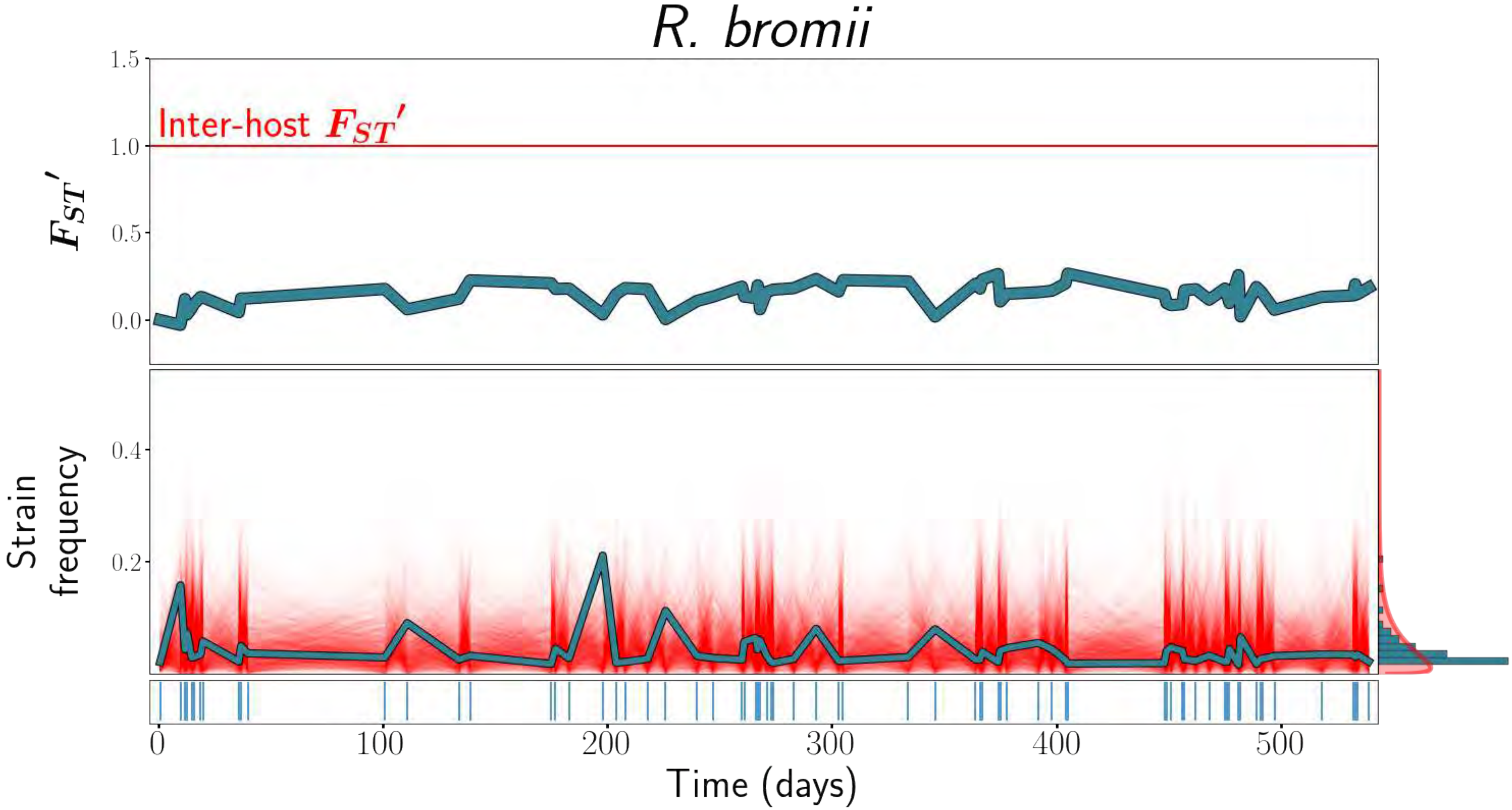
R. bromii
1.5
Inter-host F_{ST}'
1.0
F_{ST}'
0.5
0.0
0.4
Strain frequency
0.2
0
100
200
300
400
500
Time (days)
