# Supplementary material for: Ecological Stability Emerges at the Level of Strains in the Human Gut Microbiome
Source: mBio. 2023 Feb 21;14(2):e02502-22. doi: 10.1128/mbio.02502-22 (PMC10127601; doi:10.1128/mbio.02502-22)
Supplement: TEXT S3 [file mbio.02502-22-s0003.pdf]

S3 Text

F_{ST}', strain frequency, and strain abundance dynamics plots for all species analyzed, for host *ao.* These plots are analogous to Main Text Figure 1. When only a single strain was detected, only *F_{ST}'* and strain abundance dynamics plots, but no strain frequency plot, is included.

Table of contents

Bacteroides fragilis A	*1*
Bacteroides ovatus A	*2*
Bacteroides ovatus B	*3*
Bacteroides uniformis A	*4*
Bacteroides xylanisolvens A	*5*
Bacteroides xylanisolvens B	*6*
Bifidobacterium adolescentis	*7*
Dialister invisus A	*8*
Eubacterium rectale A	*9*
Faecalibacterium prausnitzii (61481) A	*10*
Faecalibacterium prausnitzii (61481) B	*11*
Faecalibacterium prausnitzii (62201) A	*12*
Lachnospira eligens A	*13*
Ruminoccocus bicirculans A	*14*
Parabacteroides distasonis A	*15*

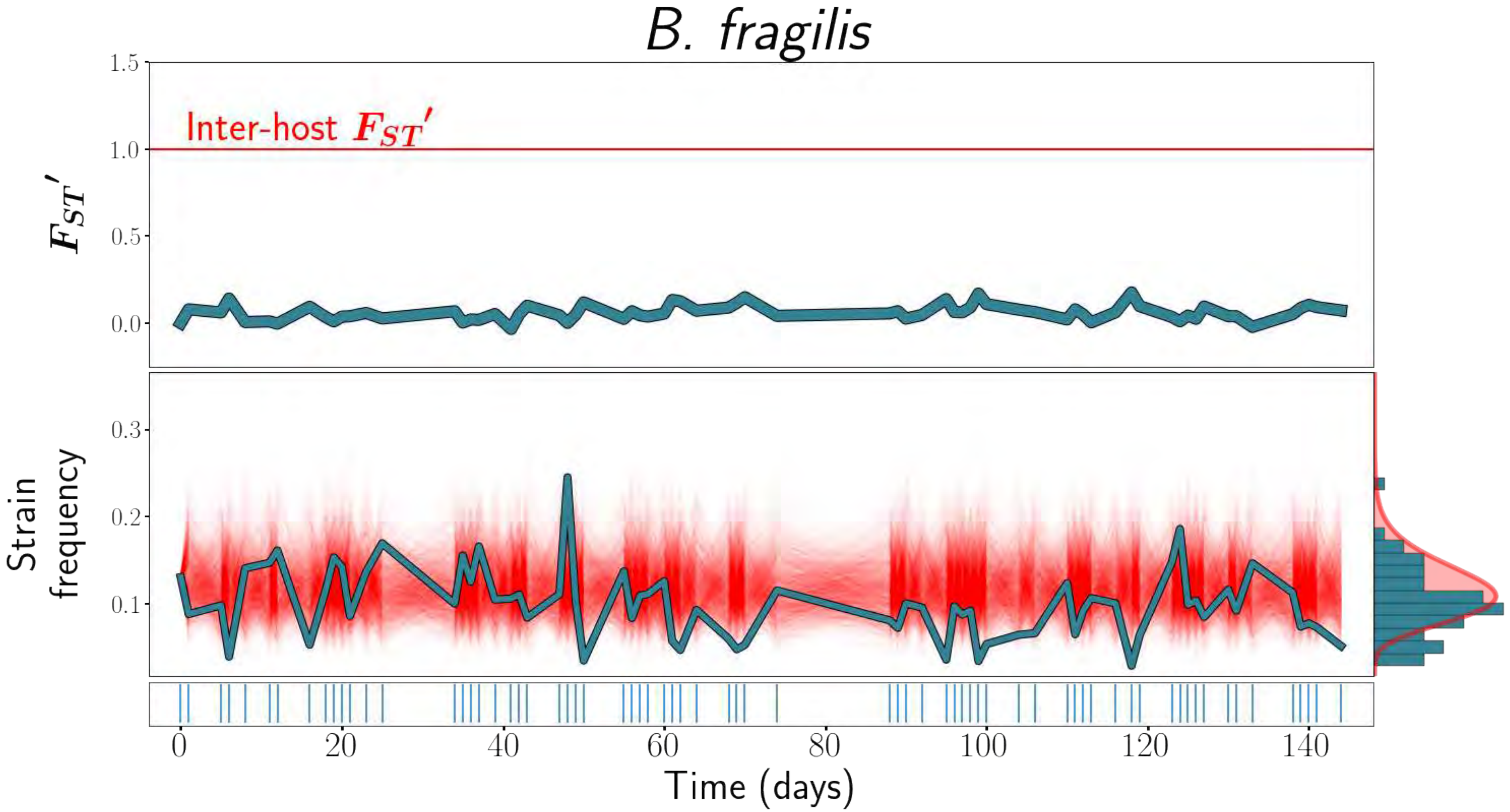
B. fragilis
Inter-host F_{ST}'
F_{ST}'
1.5
1.0
0.5
0.0
Strain frequency
0.3
0.2
0.1
0
20
40
60
80
100
120
140
Time (days)

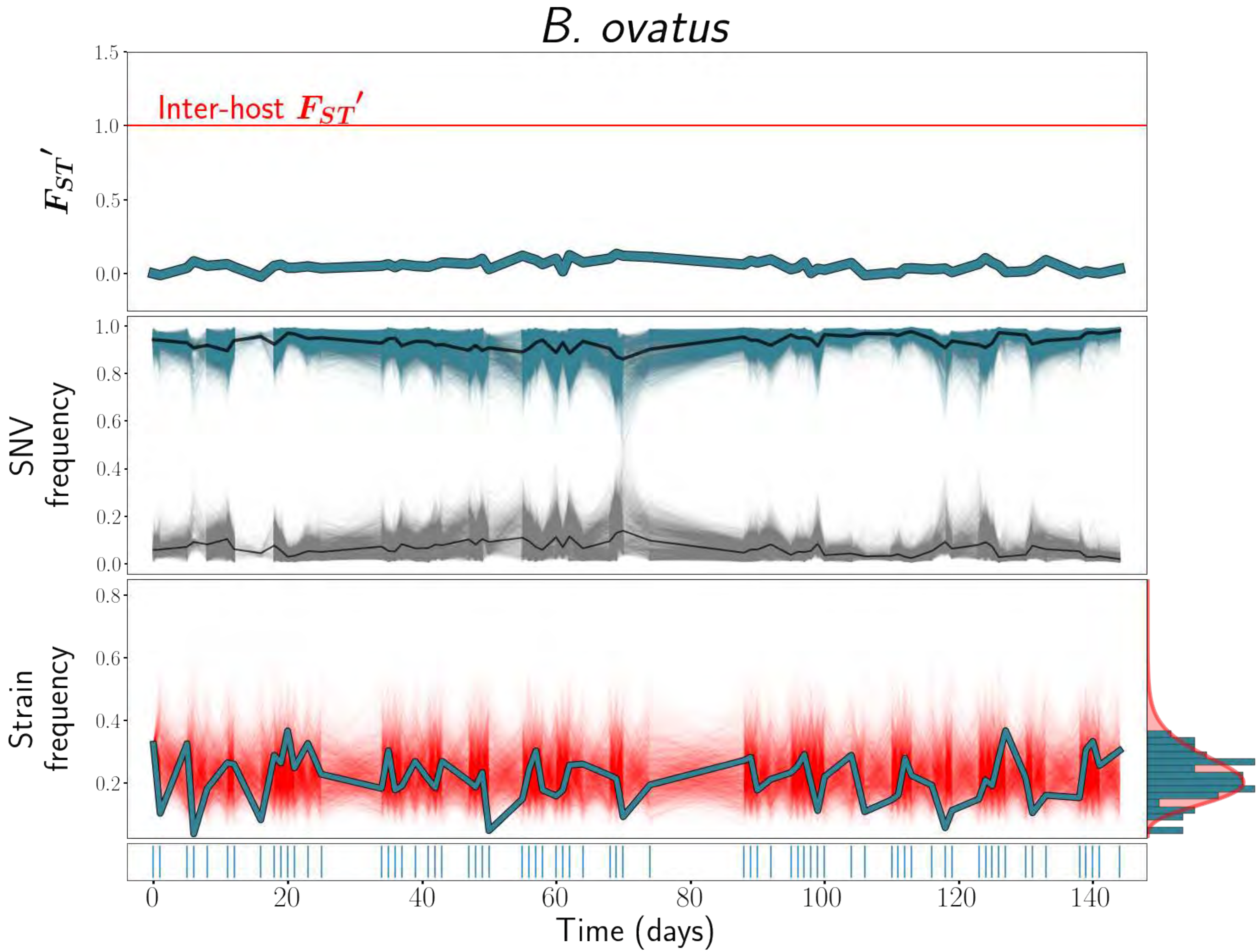

B. ovatus
Inter-host F_{ST}'
F_{ST}'
1.5
1.0
0.5
0.0
SNV frequency
1.0
0.8
0.6
0.4
0.2
0.0
Strain frequency
0.8
0.6
0.4
0.2
0
20
40
60
80
100
120
140
Time (days)

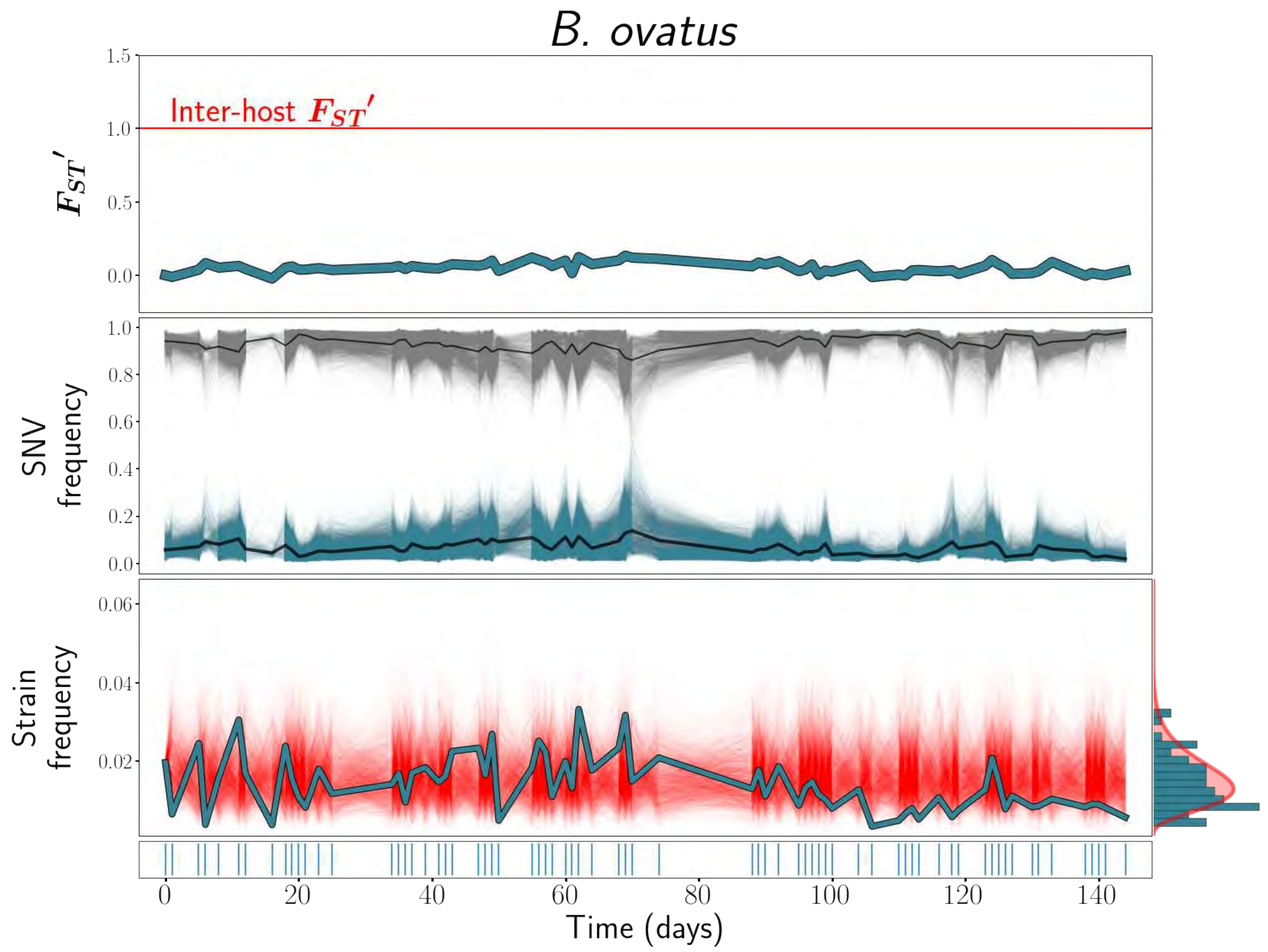

B. ovatus
Inter-host F_{ST}'
F_{ST}'
SNV frequency
Strain frequency
Time (days)

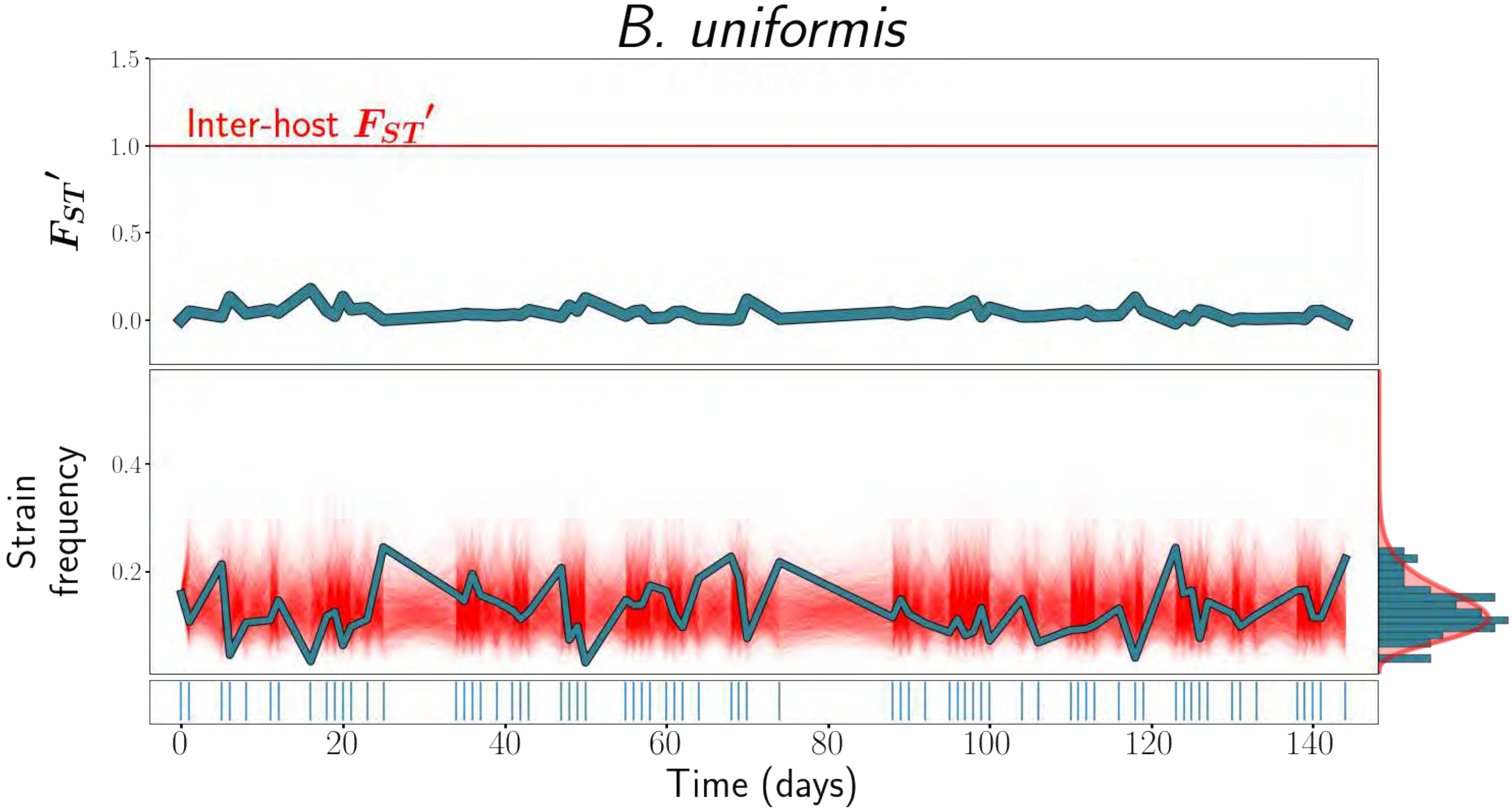

B. uniformis
Inter-host F_{ST}'
F_{ST}'
1.5
1.0
0.5
0.0
Strain frequency
0.4
0.2
0
20
40
60
80
100
120
140
Time (days)

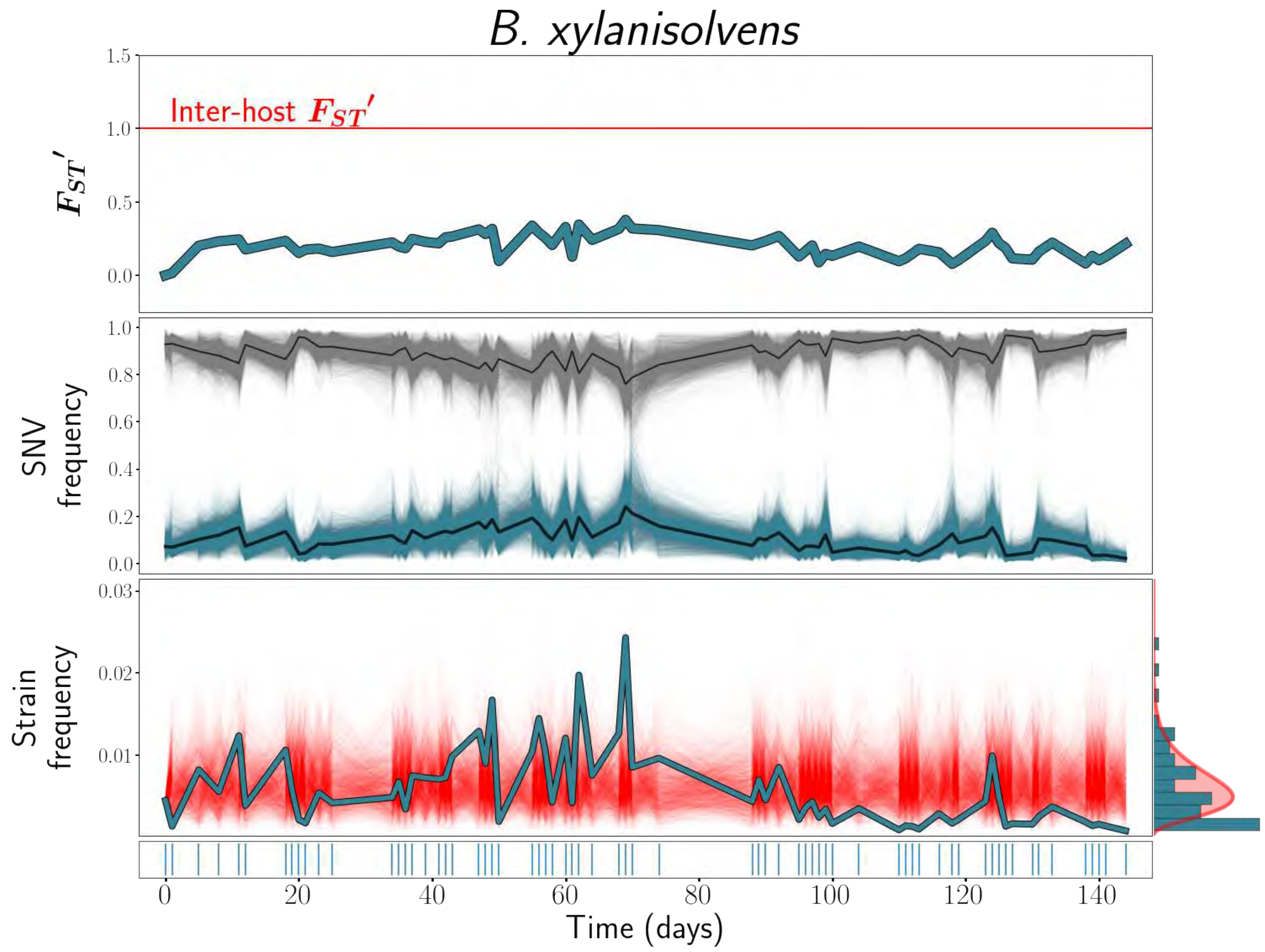
B. xylanisolvens
Inter-host F_{ST}'
F_{ST}'
1.5
1.0
0.5
0.0
SNV frequency
1.0
0.8
0.6
0.4
0.2
0.0
Strain frequency
0.03
0.02
0.01
0
20
40
60
80
100
120
140
Time (days)

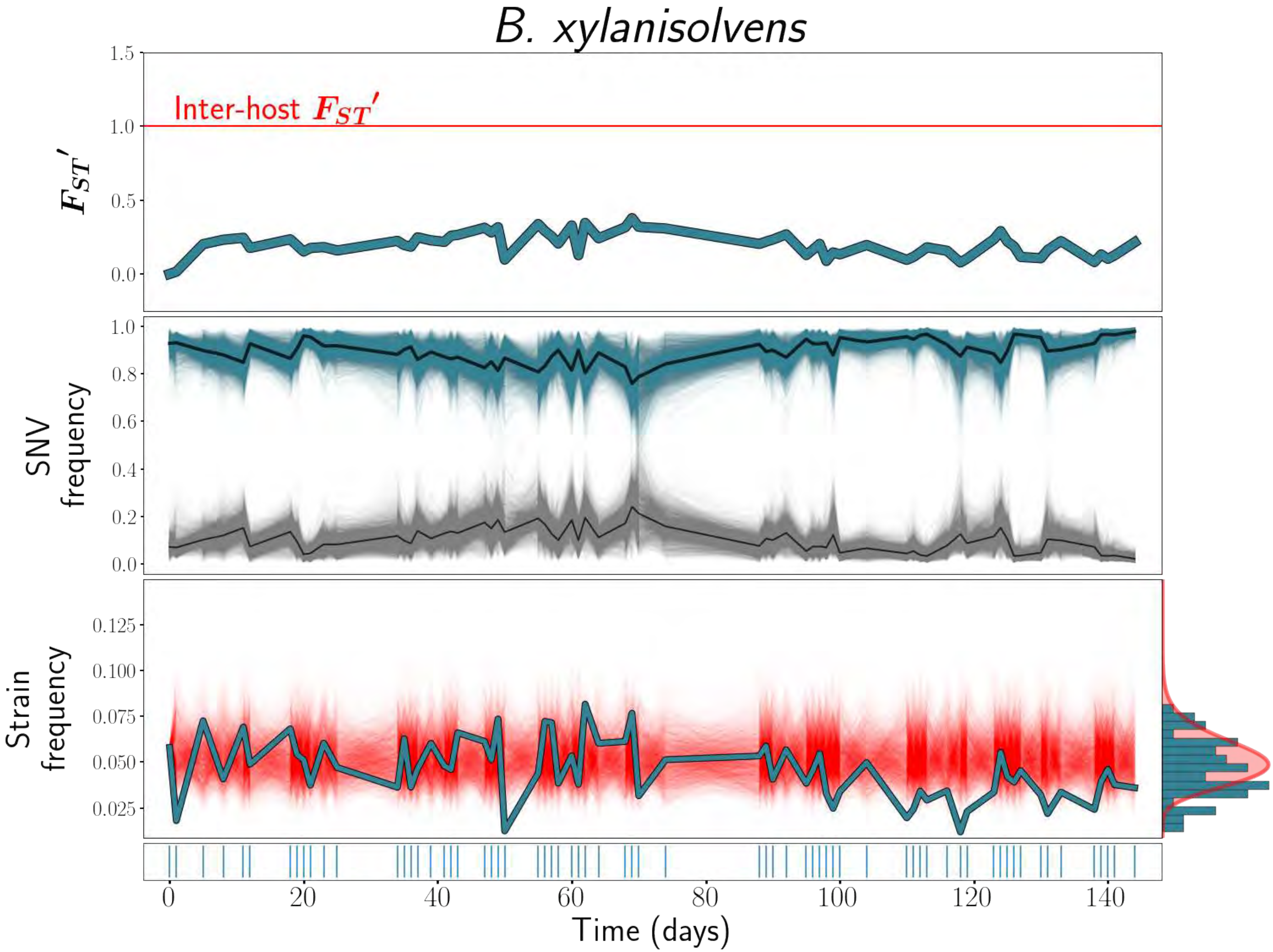

B. xylanisolvens
Inter-host F_{ST}'
F_{ST}'
1.5
1.0
0.5
0.0
SNV frequency
1.0
0.8
0.6
0.4
0.2
0.0
Strain frequency
0.125
0.100
0.075
0.050
0.025
0
20
40
60
80
100
120
140
Time (days)

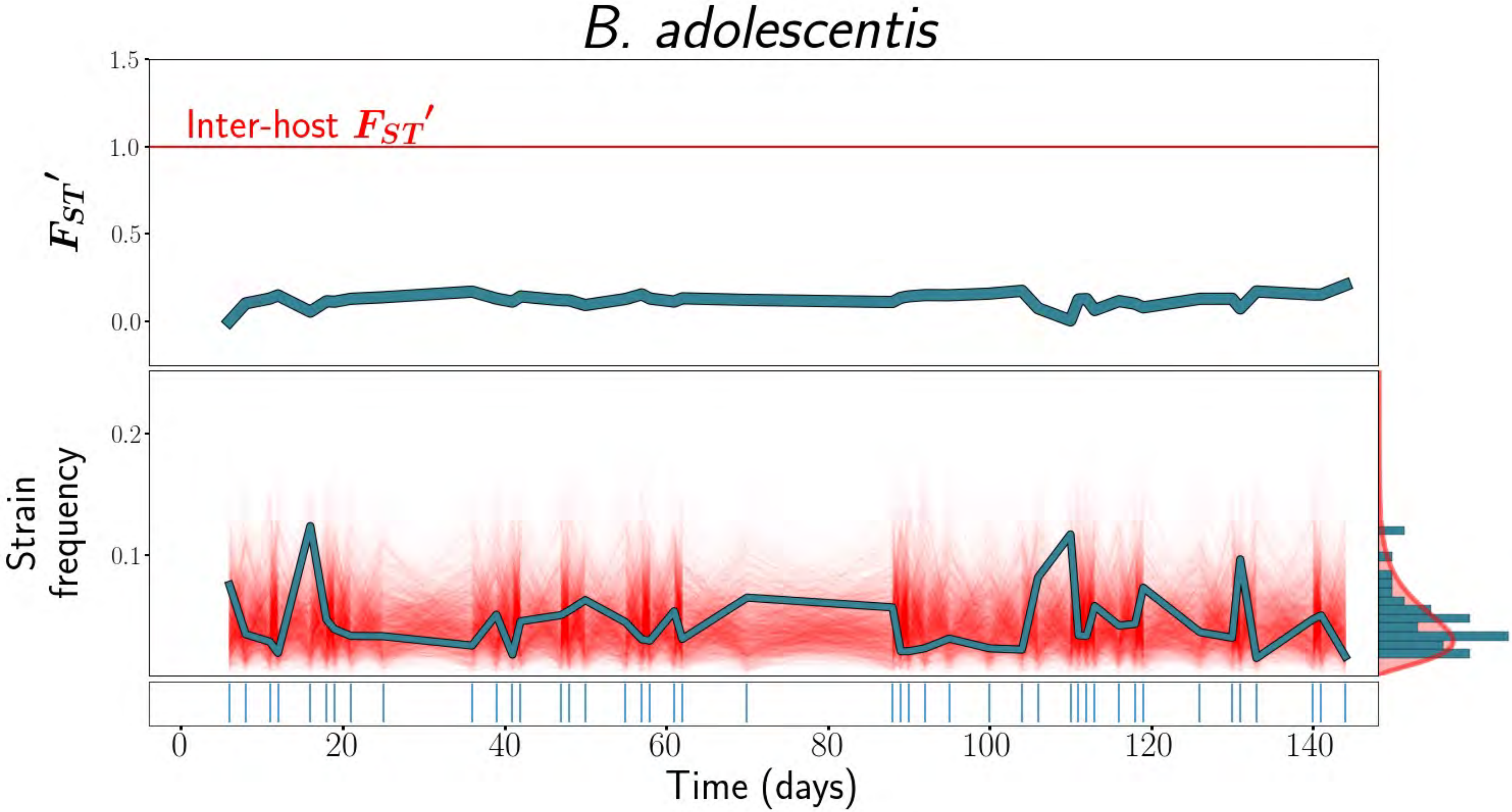
B. adolescentis
Inter-host F_{ST}'
F_{ST}'
Strain frequency
Time (days)
1.5
1.0
0.5
0.0
0.2
0.1
0
20
40
60
80
100
120
140

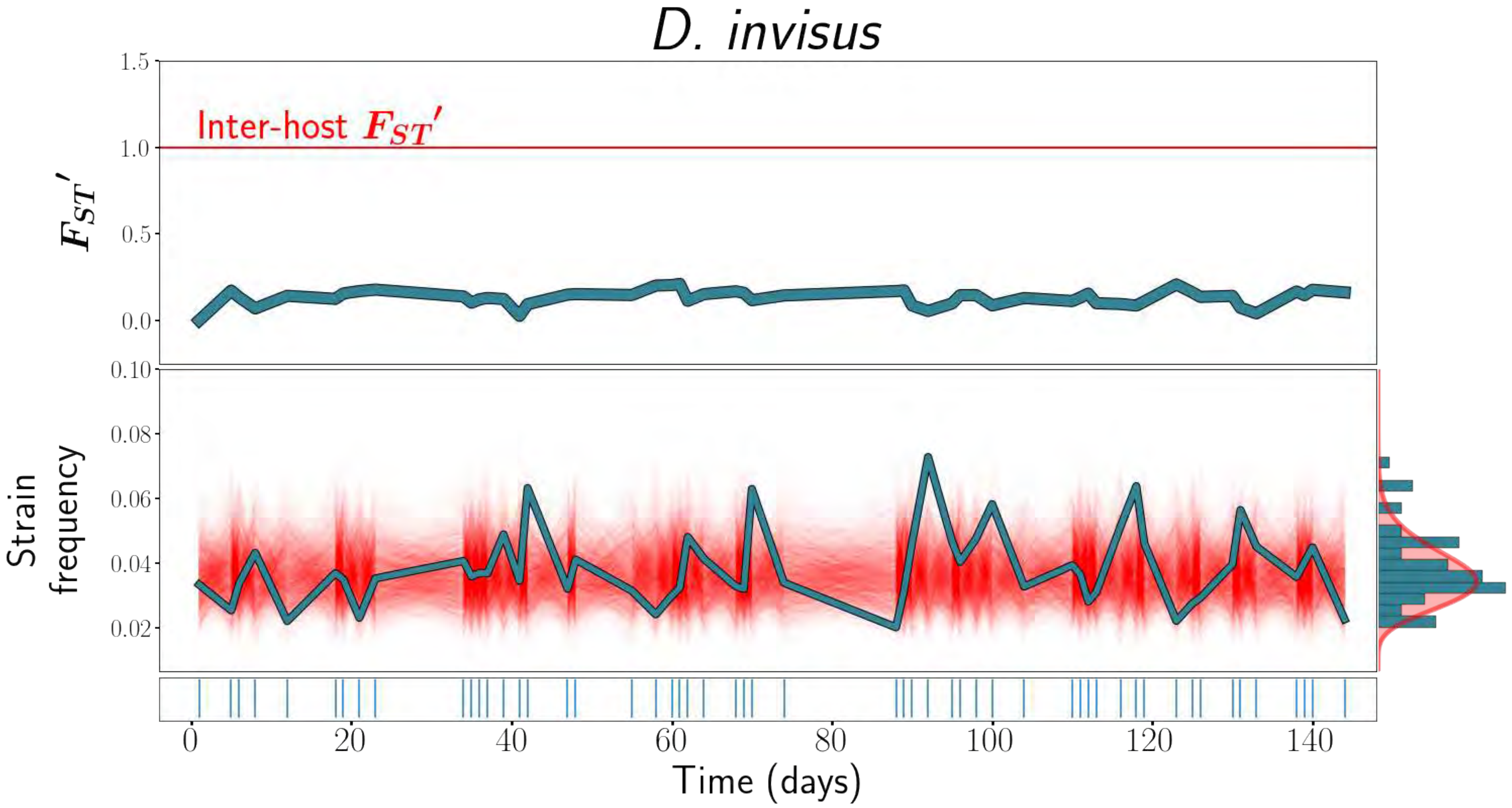
D. invisus
Inter-host F_{ST}'
F_{ST}'
1.5
1.0
0.5
0.0
0.10
0.08
0.06
0.04
0.02
Strain frequency
0
20
40
60
80
100
120
140
Time (days)

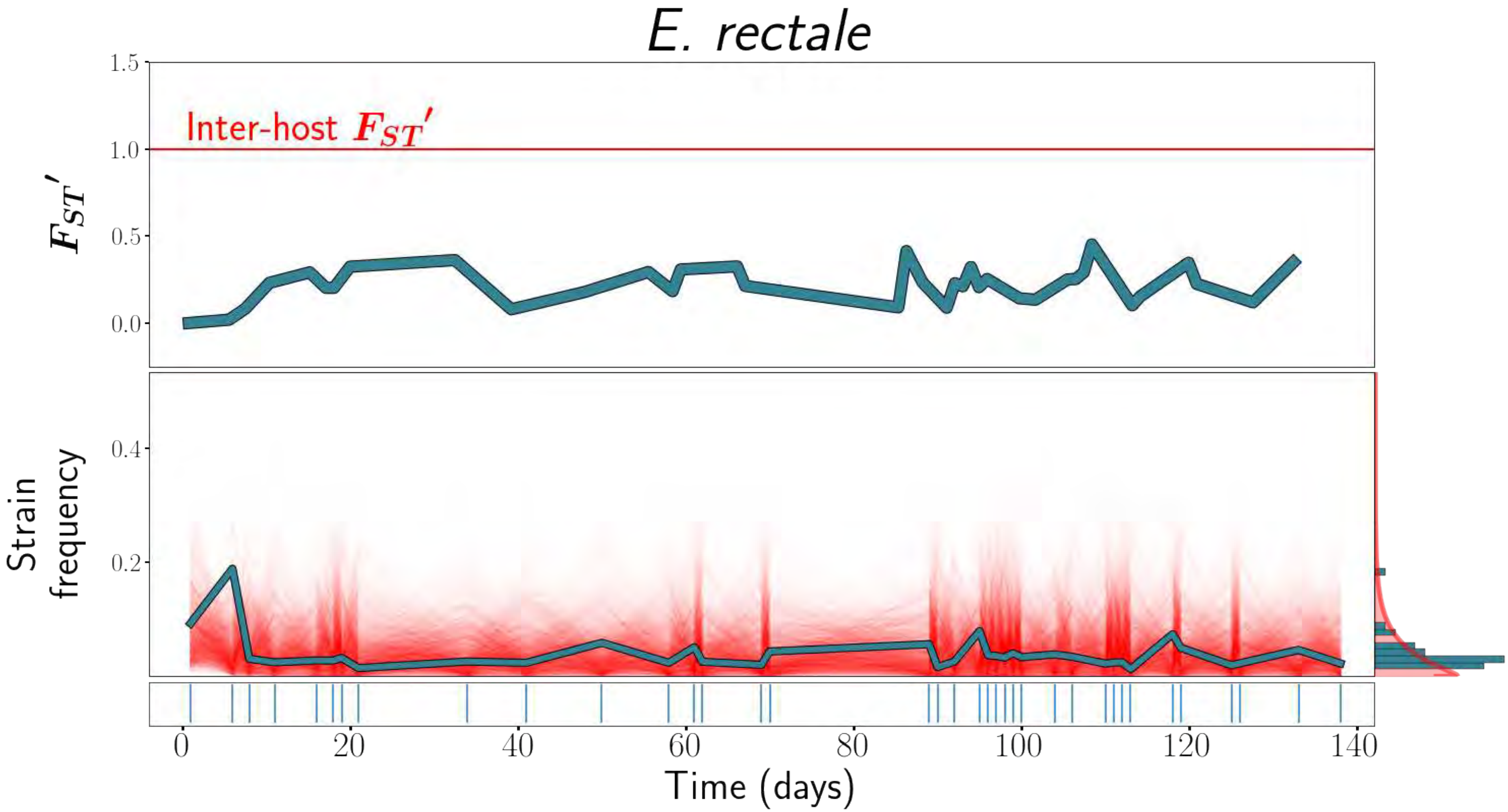
E. rectale
1.5
Inter-host F_{ST}'
1.0
F_{ST}'
0.5
0.0
0.4
Strain frequency
0.2
0
20
40
60
80
100
120
140
Time (days)

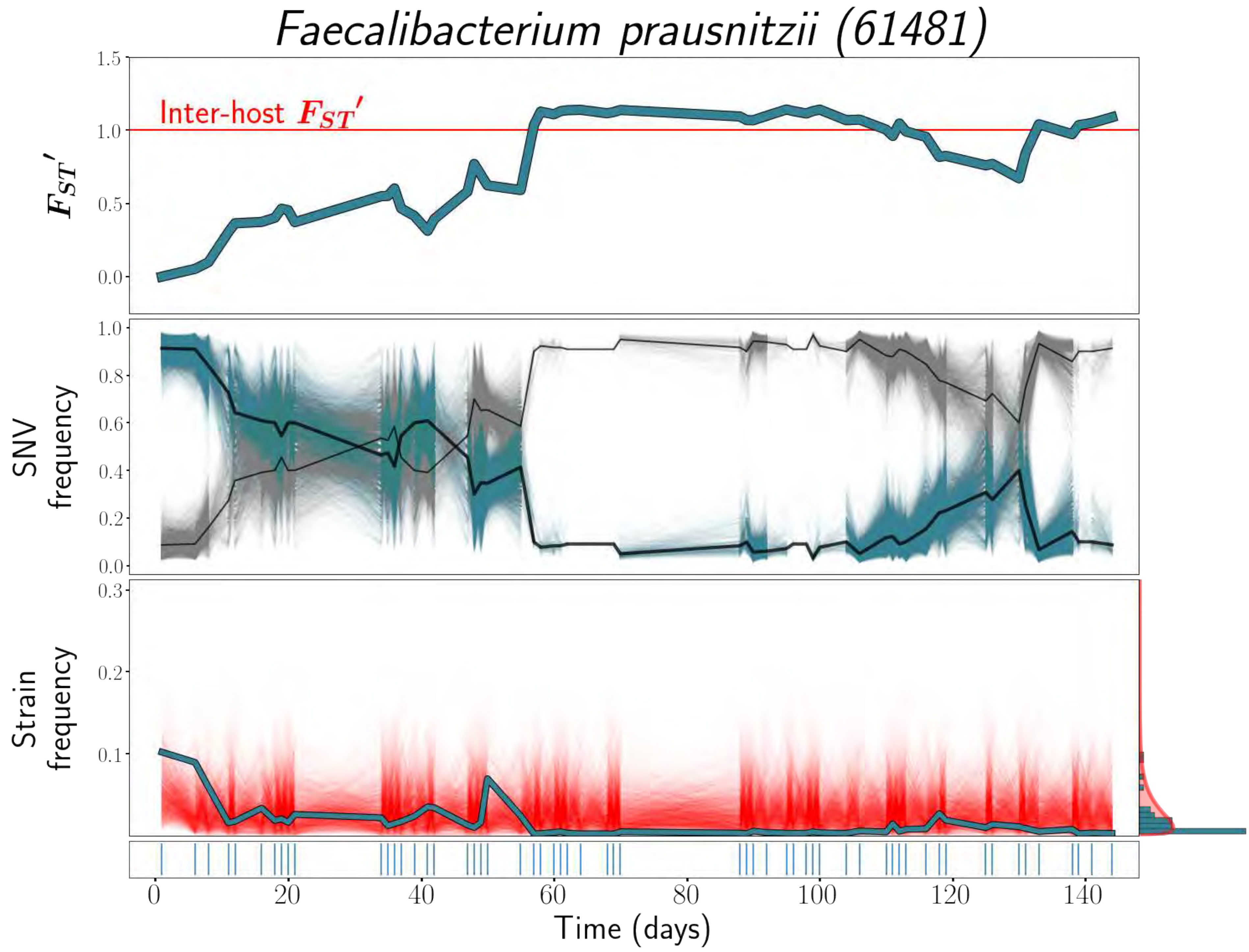

Faecalibacterium prausnitzii (61481)
Inter-host F_{ST}'
F_{ST}'
1.5
1.0
0.5
0.0
SNV frequency
1.0
0.8
0.6
0.4
0.2
0.0
Strain frequency
0.3
0.2
0.1
0
20
40
60
80
100
120
140
Time (days)

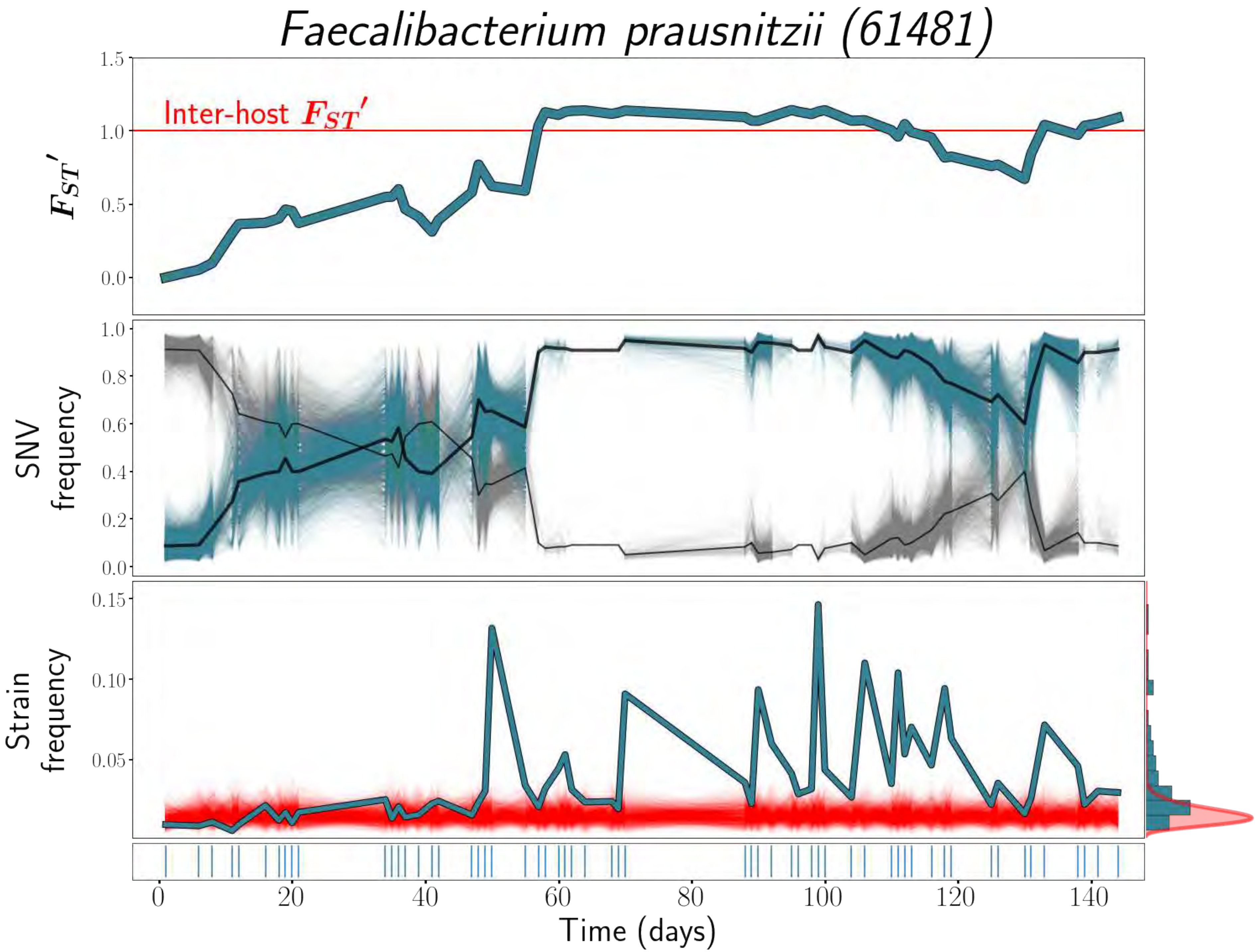
Faecalibacterium prausnitzii (61481)
Inter-host F_{ST}'
F_{ST}'
1.5
1.0
0.5
0.0
SNV frequency
1.0
0.8
0.6
0.4
0.2
0.0
Strain frequency
0.15
0.10
0.05
0
20
40
60
80
100
120
140
Time (days)

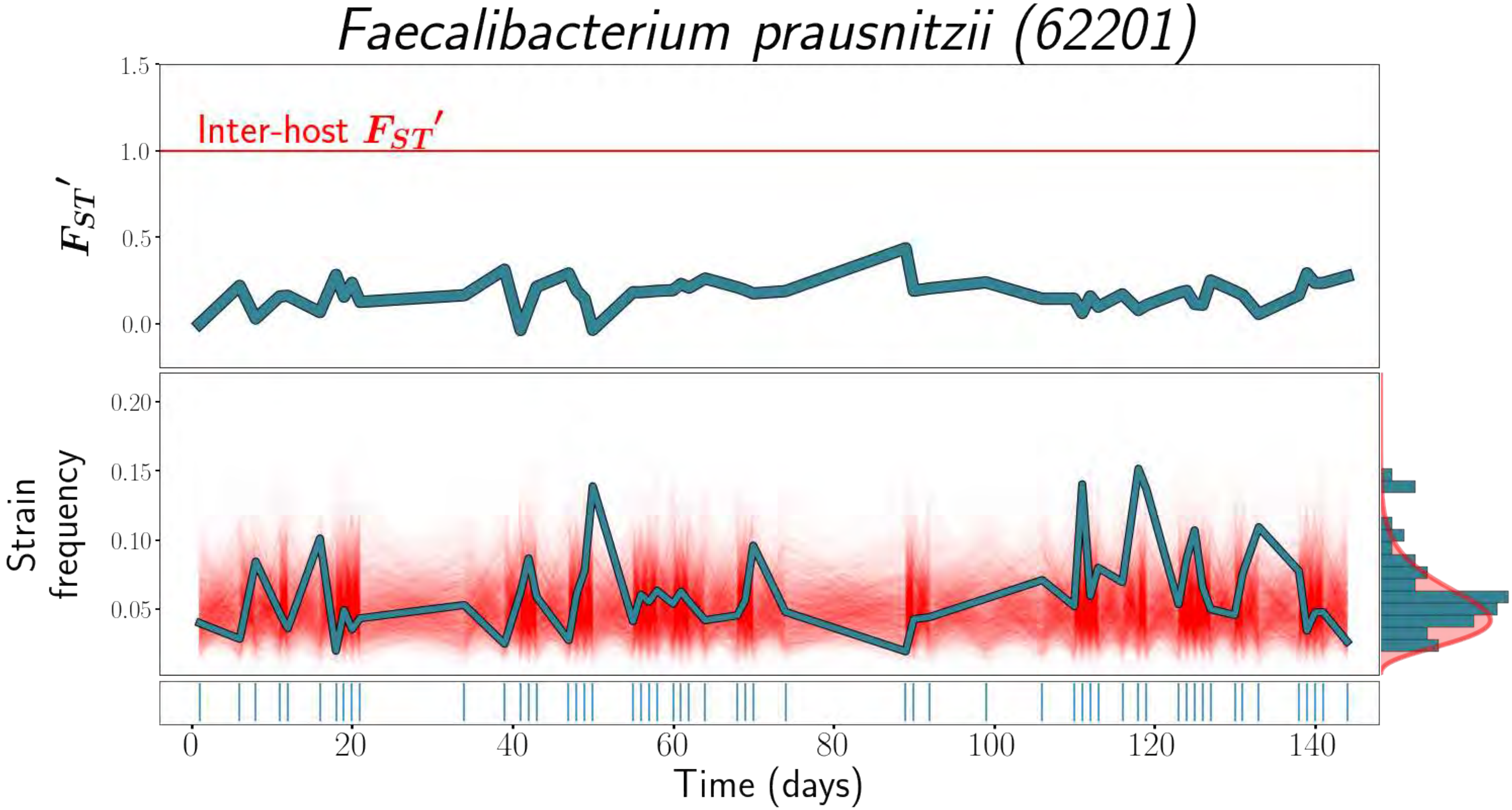

Faecalibacterium prausnitzii (62201)
Inter-host F_{ST}'
F_{ST}'
1.5
1.0
0.5
0.0
Strain frequency
0.20
0.15
0.10
0.05
0
20
40
60
80
100
120
140
Time (days)

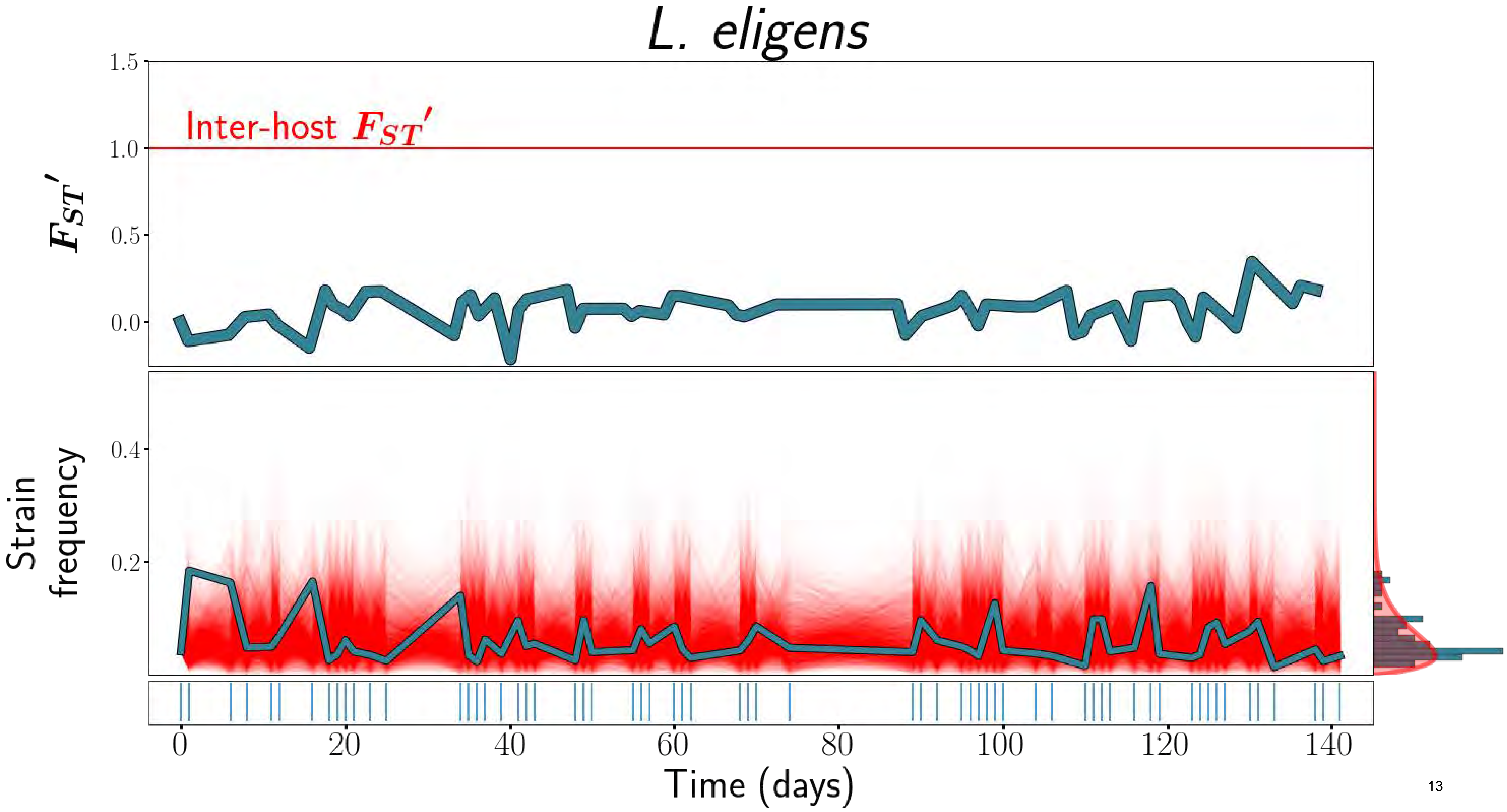
L. eligens
Inter-host F_{ST}'
F_{ST}'
1.5
1.0
0.5
0.0
Strain frequency
0.4
0.2
0
20
40
60
80
100
120
140
Time (days)

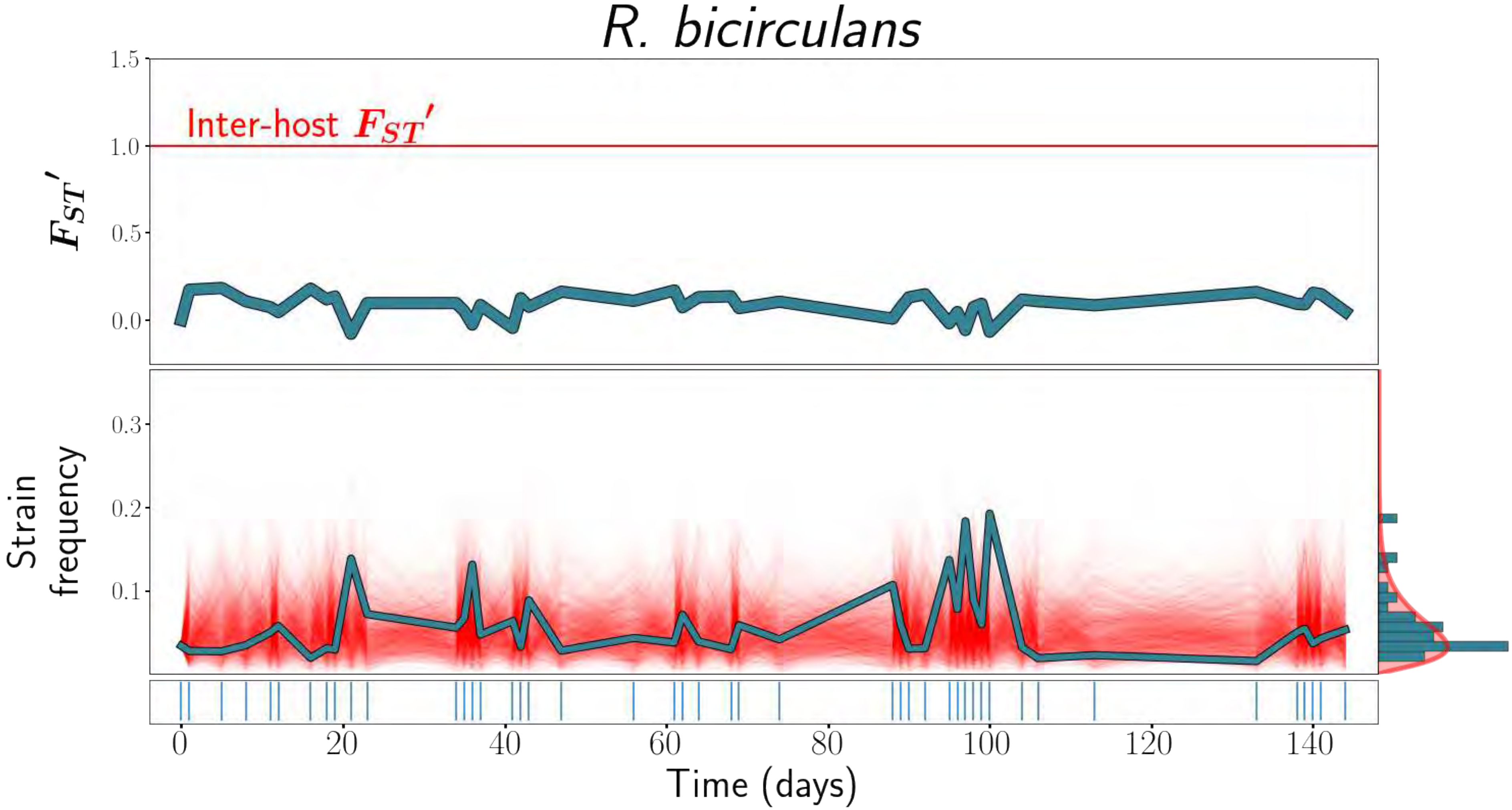
R. bicirculans
Inter-host F_{ST}'
F_{ST}'
1.5
1.0
0.5
0.0
Strain frequency
0.3
0.2
0.1
0
20
40
60
80
100
120
140
Time (days)

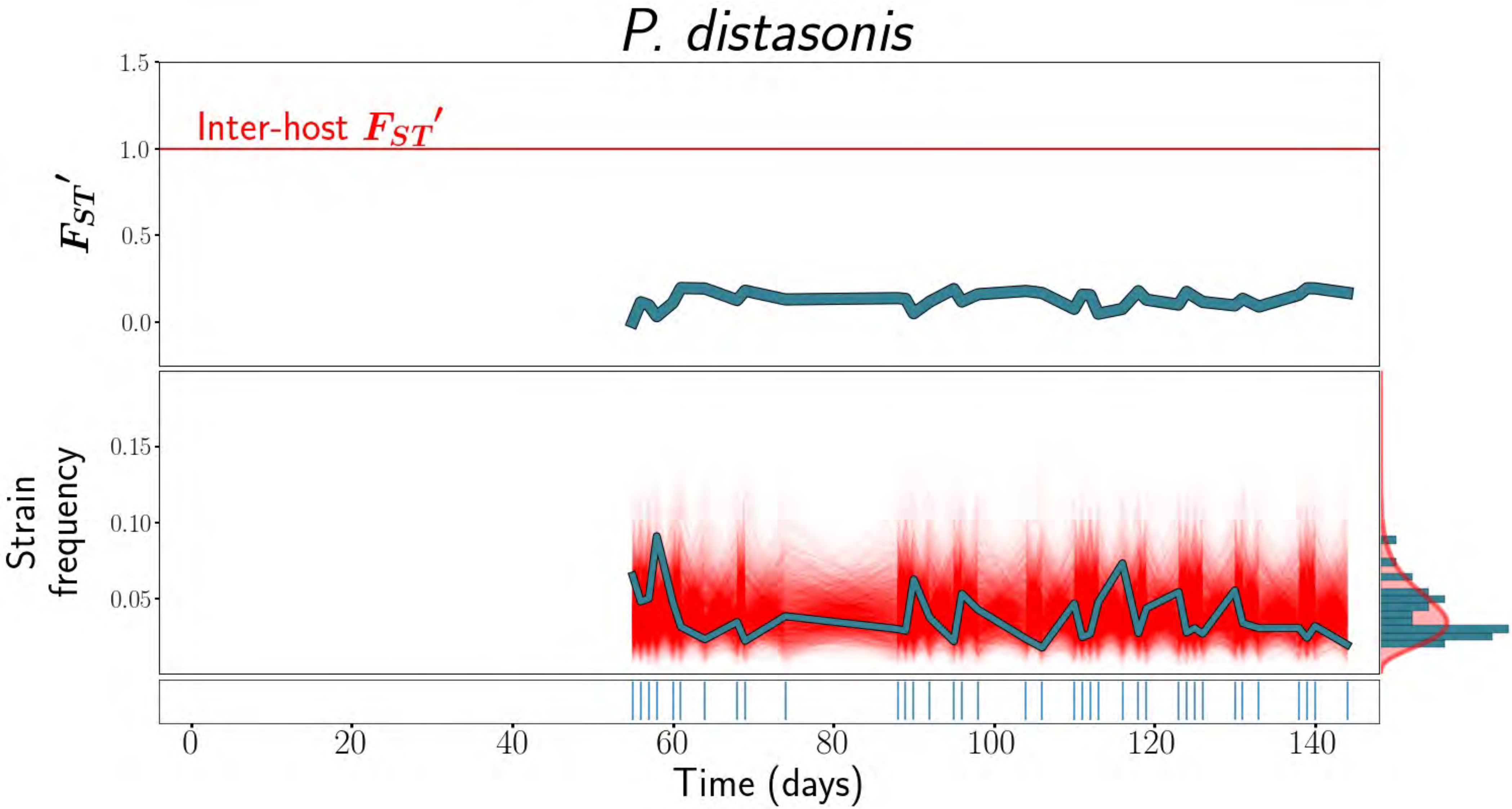
P. distasonis
Inter-host F_{ST}'
F_{ST}'
1.5
1.0
0.5
0.0
Strain frequency
0.15
0.10
0.05
0
20
40
60
80
100
120
140
Time (days)
