# Supplementary material for: Ecological Stability Emerges at the Level of Strains in the Human Gut Microbiome
Source: mBio. 2023 Feb 21;14(2):e02502-22. doi: 10.1128/mbio.02502-22 (PMC10127601; doi:10.1128/mbio.02502-22)
Supplement: TEXT S4 [file mbio.02502-22-s0004.pdf]

S4 Text

F_{ST}', strain frequency, and strain abundance dynamics plots for all species analyzed, for host *an.* These plots are analogous to Main Text Figure 1. When only a single strain was detected, only *F_{ST}'* and strain abundance dynamics plots, but no strain frequency plot, is included.

Table of contents

Alistipes putredinis A 1
Bacteroides clarus A 2
Bacteroides uniformis A 3
Bacteroides uniformis B 4
Bacteroides xylanisolvens A 5
Barnesiella intestinihominis A 6
Eubacterium rectale A 7
Eubacterium rectale B 8
Paraprevotella clara A 9
Phocaeicola massilensis A 10
Phocaeicola vulgatus A 11
Phocaeicola vulgatus B 12
Phocaeicola vulgatus C 13
Ruminoccocus bromii A 14
Sutterella wadsworthensis A 15
Sutterella wadsworthensis B 16

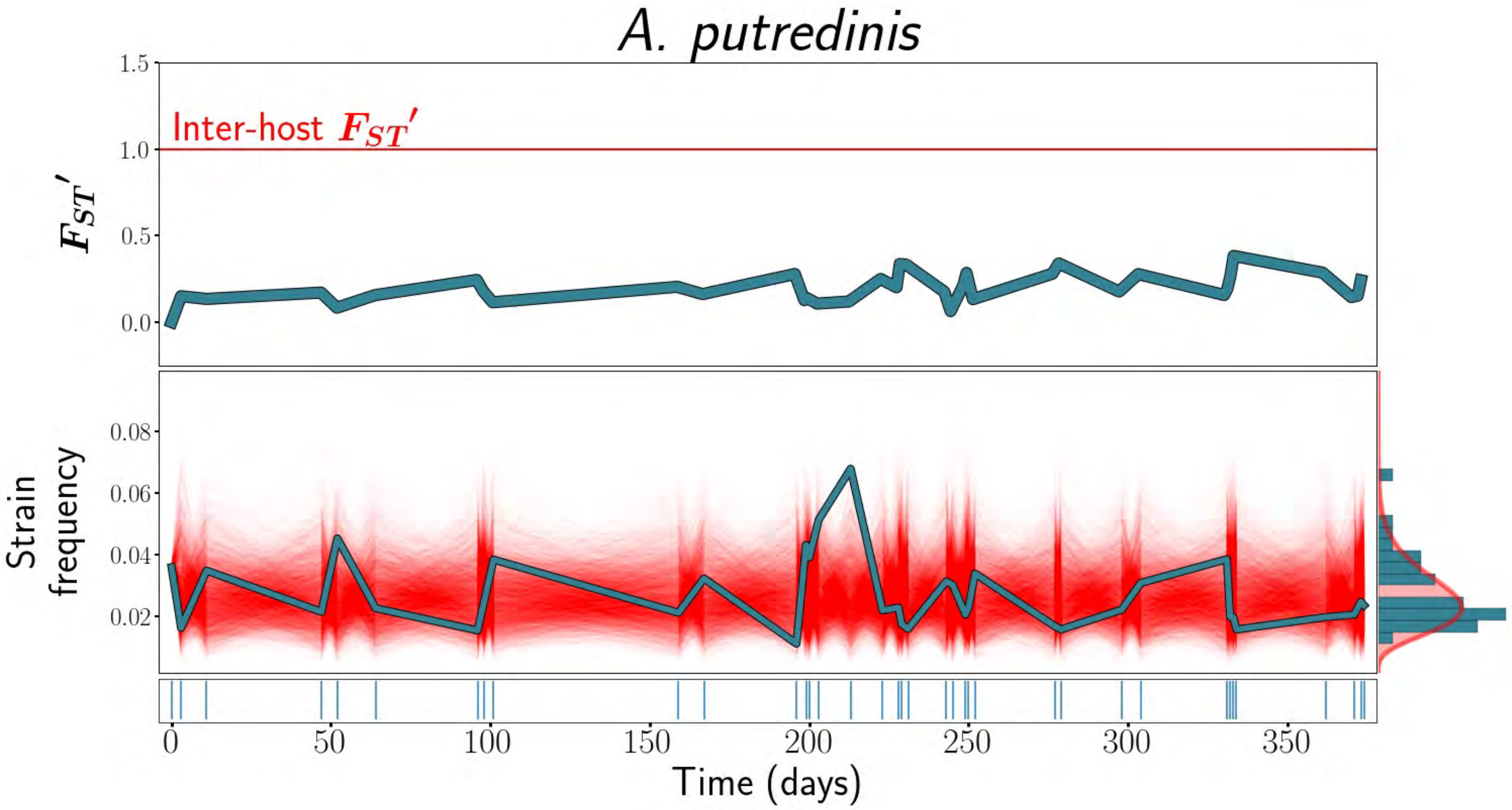

A. putredinis
Inter-host F_{ST}'
F_{ST}'
Strain frequency
Time (days)

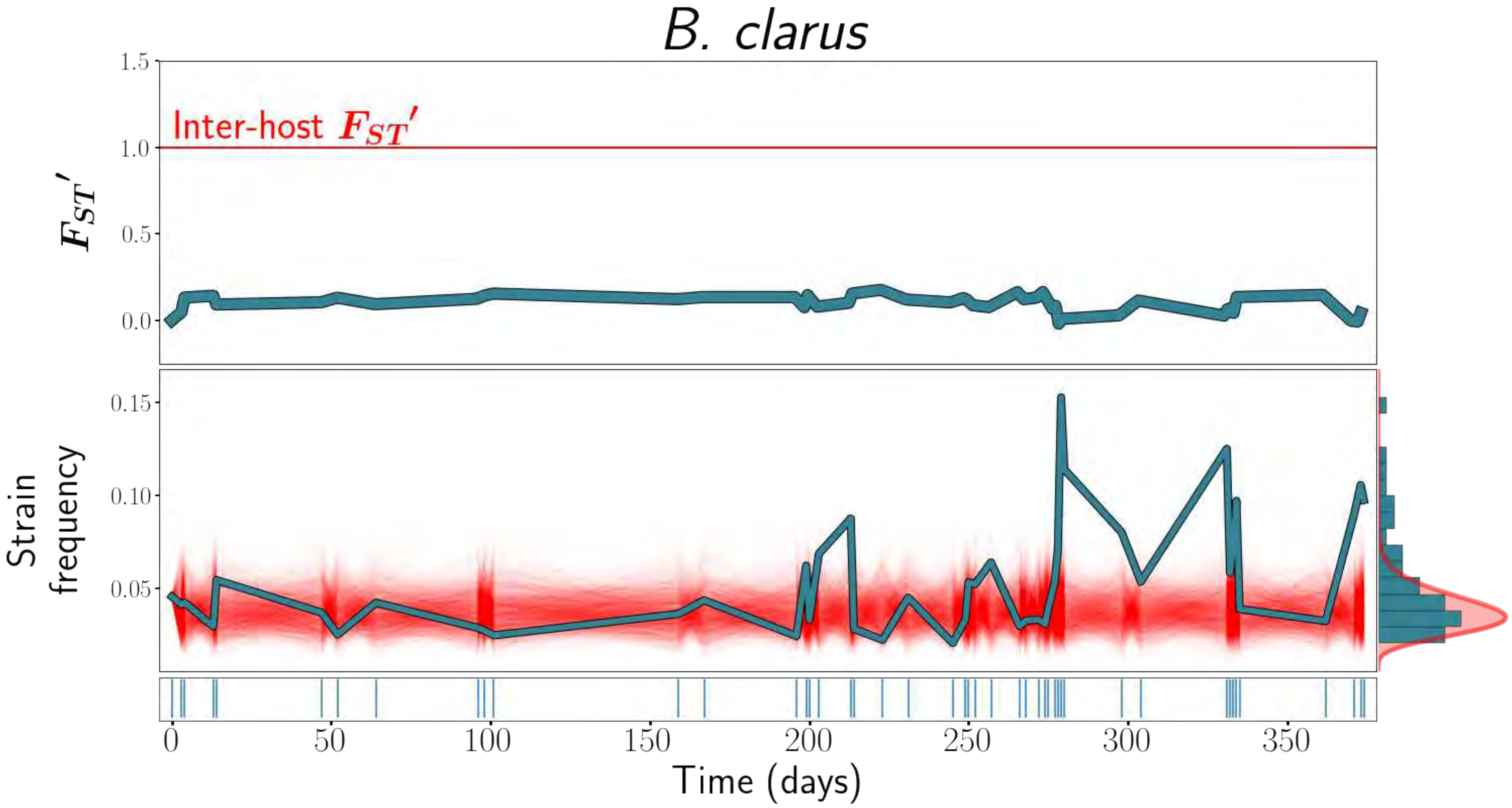
B. clarus
Inter-host F_{ST}'
F_{ST}'
1.5
1.0
0.5
0.0
Strain frequency
0.15
0.10
0.05
0
50
100
150
200
250
300
350
Time (days)

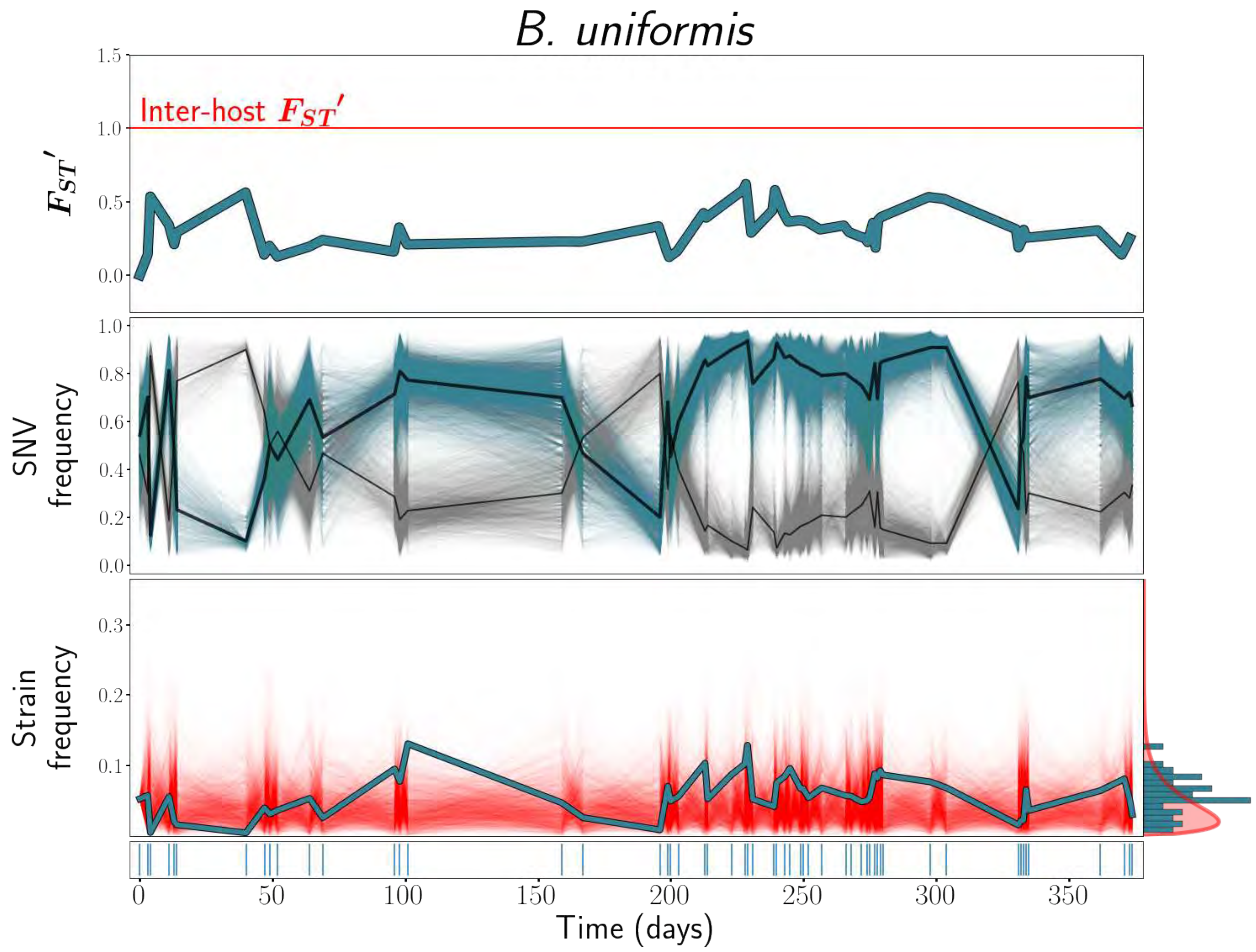
B. uniformis
Inter-host F_{ST}'
F_{ST}'
SNV frequency
Strain frequency
Time (days)
1.5
1.0
0.5
0.0
1.0
0.8
0.6
0.4
0.2
0.0
0.3
0.2
0.1
0
50
100
150
200
250
300
350

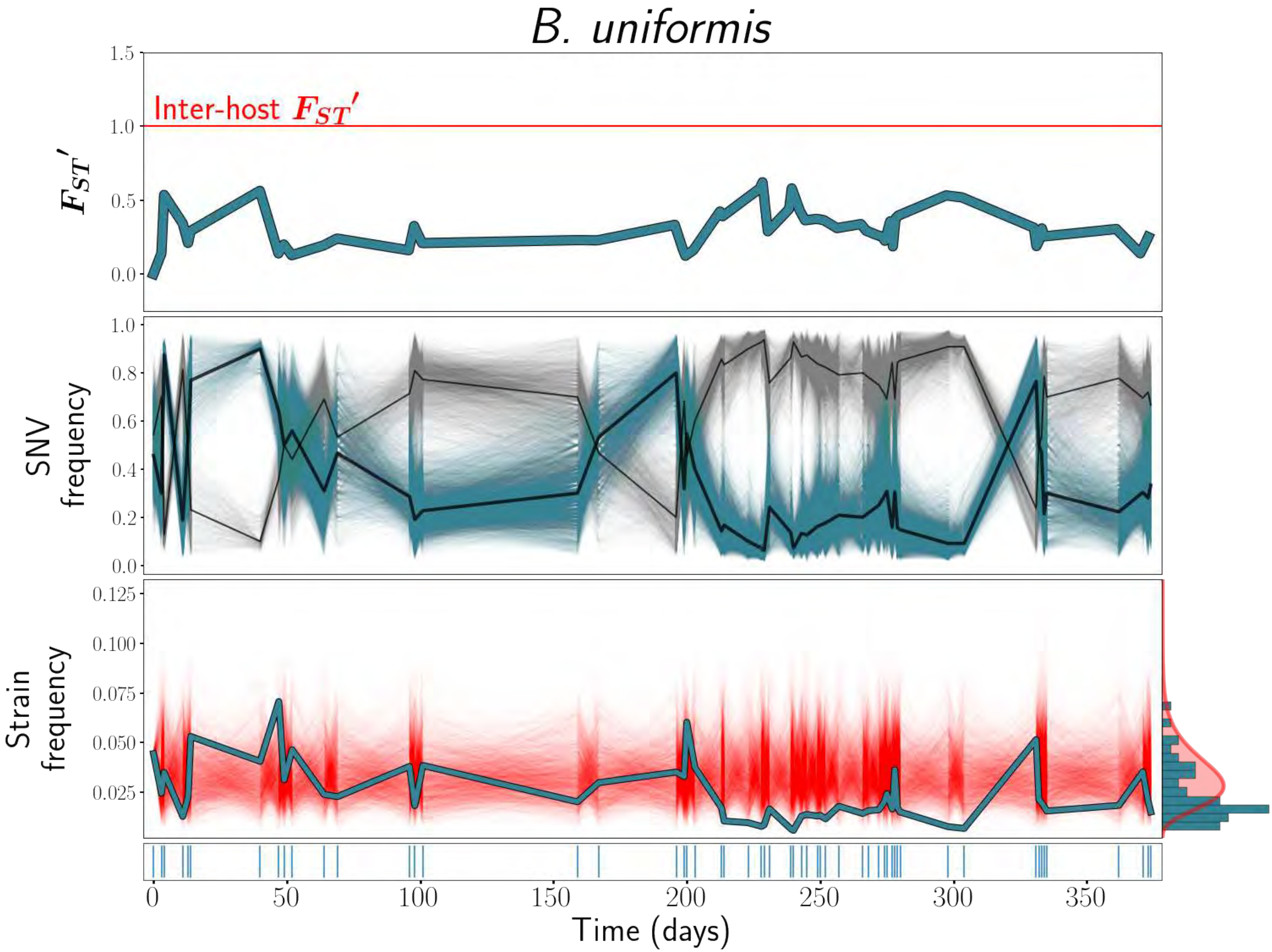

B. uniformis
Inter-host F_{ST}'
F_{ST}'
1.5
1.0
0.5
0.0
SNV frequency
1.0
0.8
0.6
0.4
0.2
0.0
Strain frequency
0.125
0.100
0.075
0.050
0.025
0
50
100
150
200
250
300
350
Time (days)

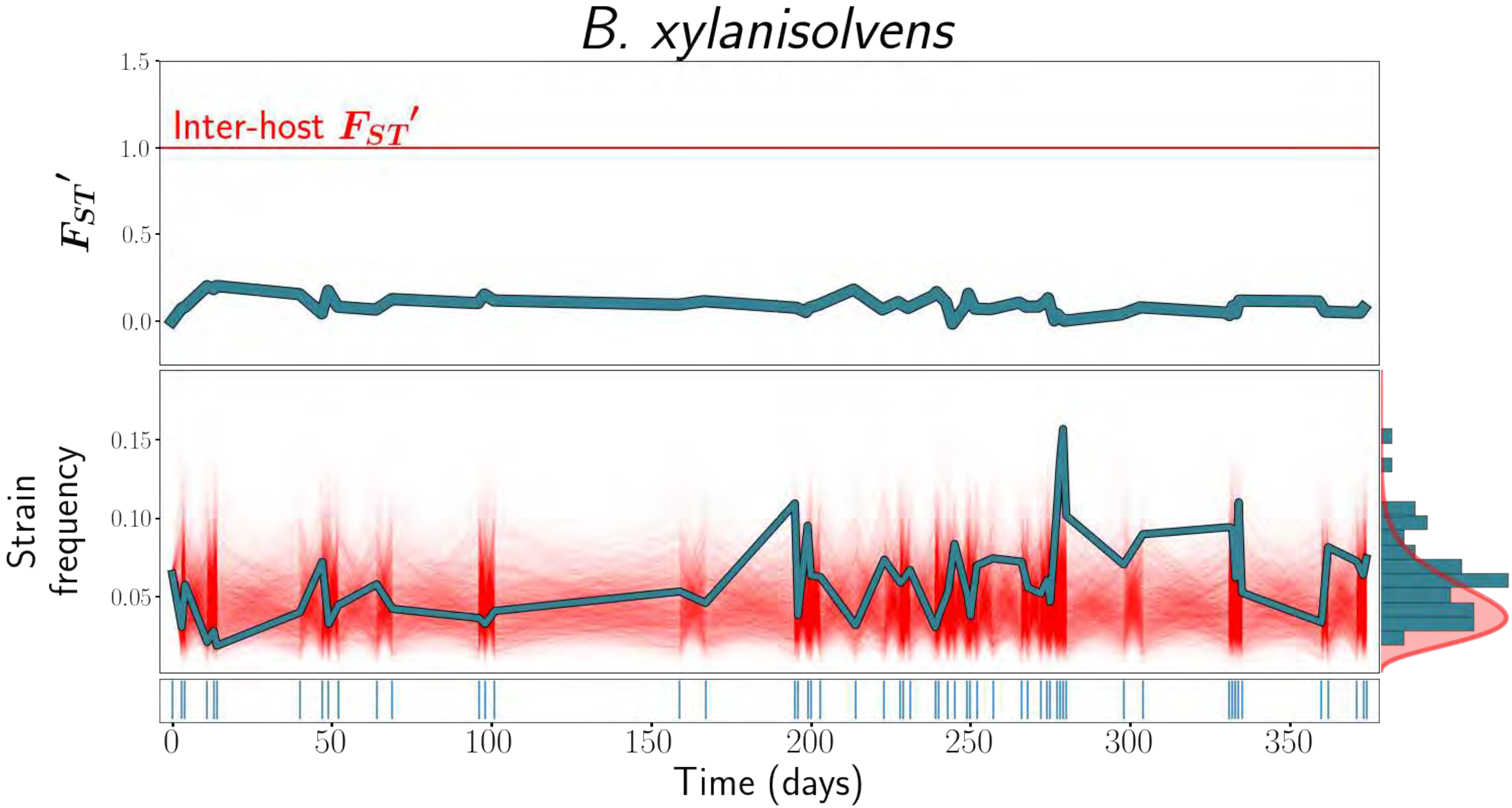

B. xylanisolvens
Inter-host F_{ST}'
F_{ST}'
1.5
1.0
0.5
0.0
Strain frequency
0.15
0.10
0.05
0
50
100
150
200
250
300
350
Time (days)

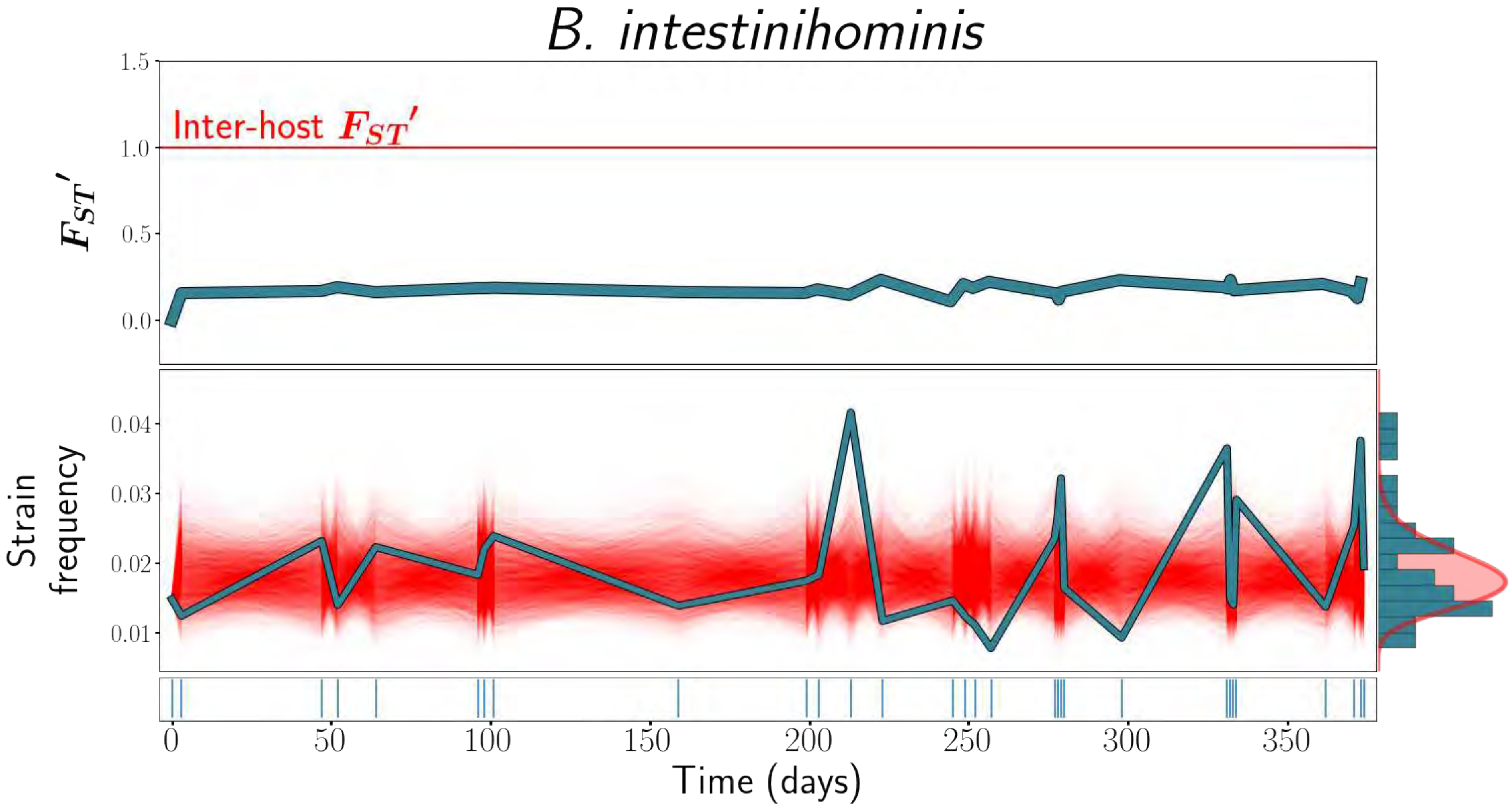

B. intestinihominis
Inter-host F_{ST}'
F_{ST}'
1.5
1.0
0.5
0.0
Strain frequency
0.04
0.03
0.02
0.01
0
50
100
150
200
250
300
350
Time (days)

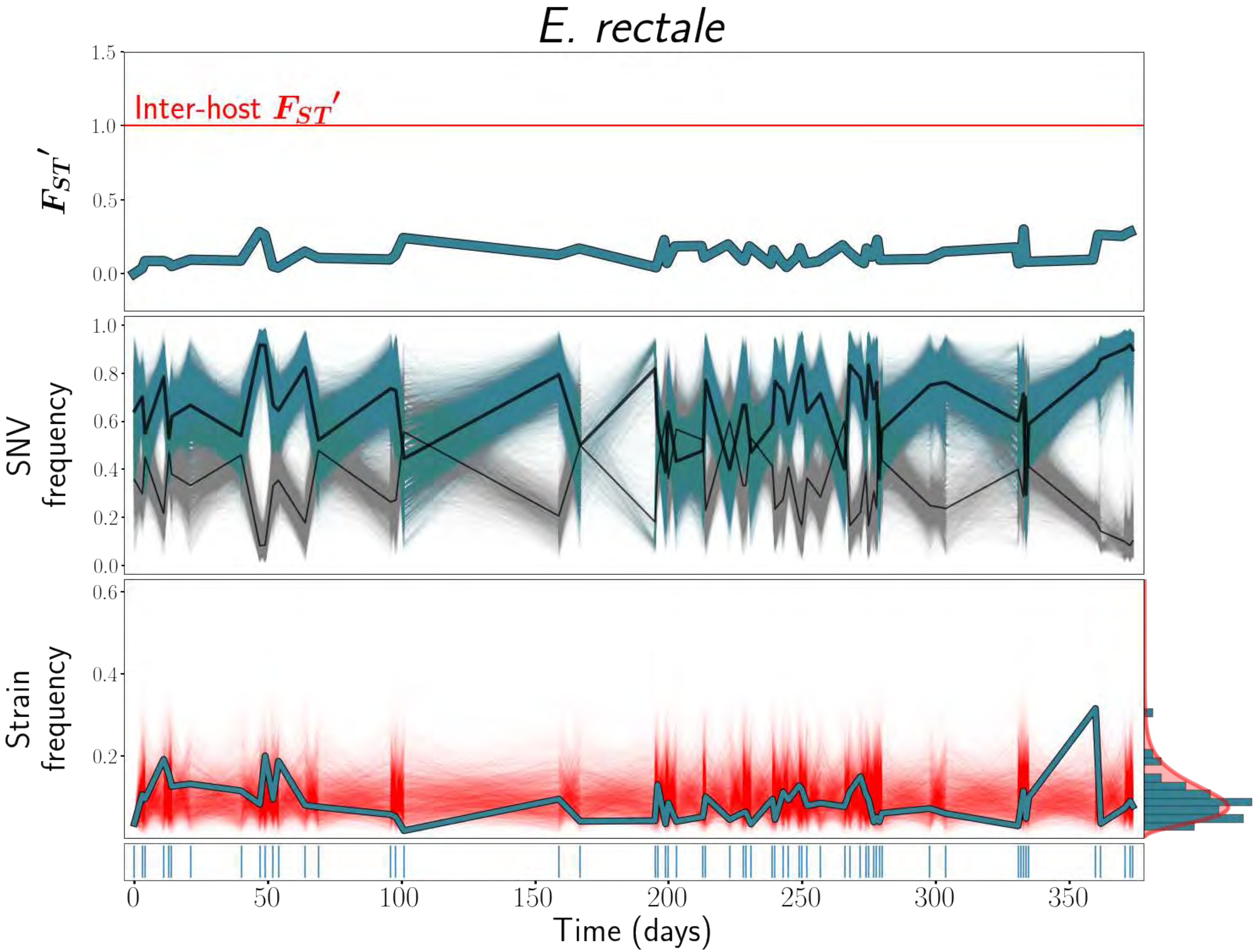
E. rectale
Inter-host F_{ST}'
F_{ST}'
1.5
1.0
0.5
0.0
SNV frequency
1.0
0.8
0.6
0.4
0.2
0.0
Strain frequency
0.6
0.4
0.2
0
50
100
150
200
250
300
350
Time (days)

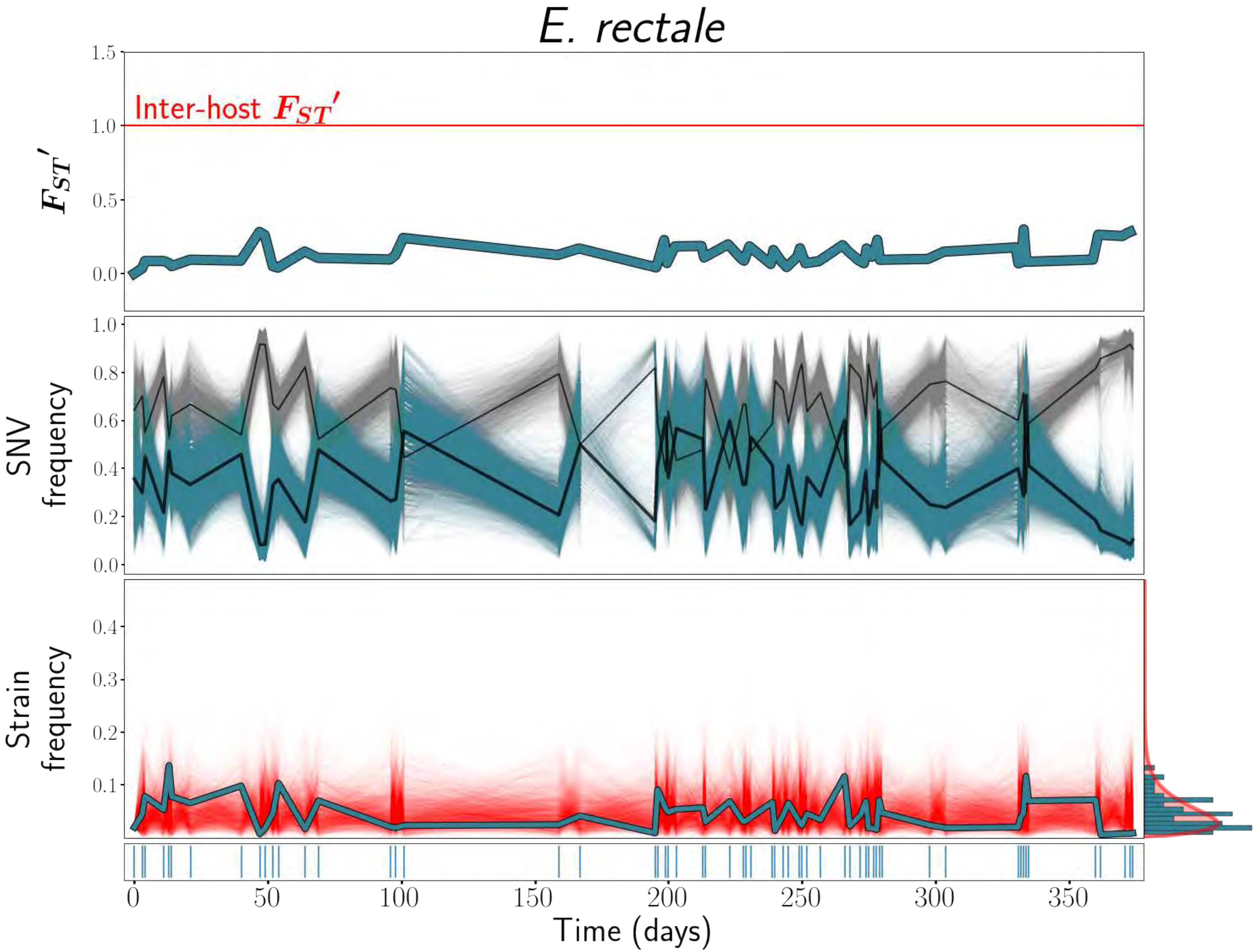
E. rectale
Inter-host F_{ST}'
F_{ST}'
SNV frequency
Strain frequency
Time (days)
1.5
1.0
0.5
0.0
0.8
0.6
0.4
0.2
0.3
0.1
0
50
100
150
200
250
300
350

P. clara

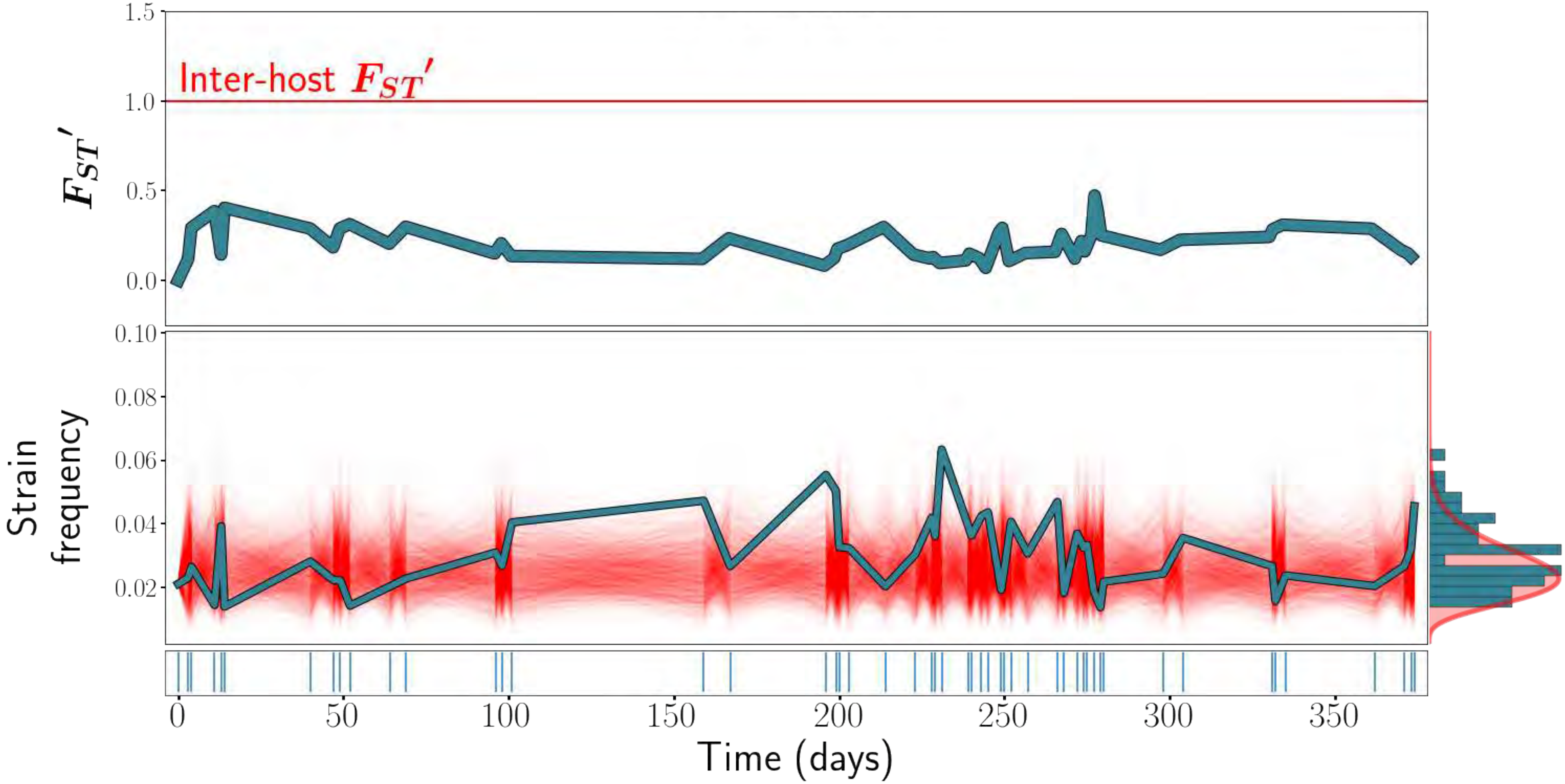

Inter-host F_{ST}'
F_{ST}'
1.5
1.0
0.5
0.0
Strain frequency
0.10
0.08
0.06
0.04
0.02
0
50
100
150
200
250
300
350
Time (days)

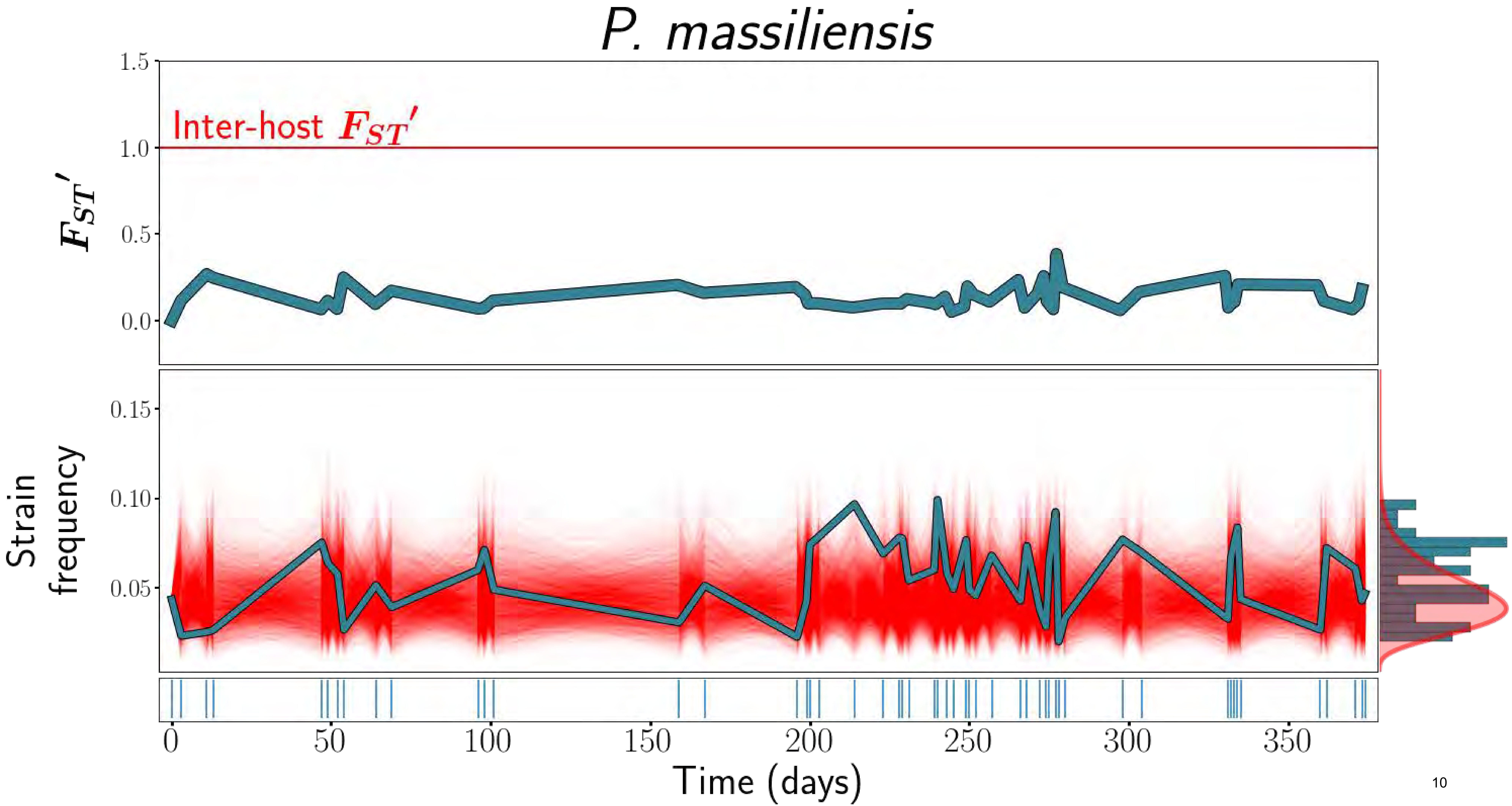
P. massiliensis
Inter-host F_{ST}'
F_{ST}'
1.5
1.0
0.5
0.0
Strain frequency
0.15
0.10
0.05
0
50
100
150
200
250
300
350
Time (days)

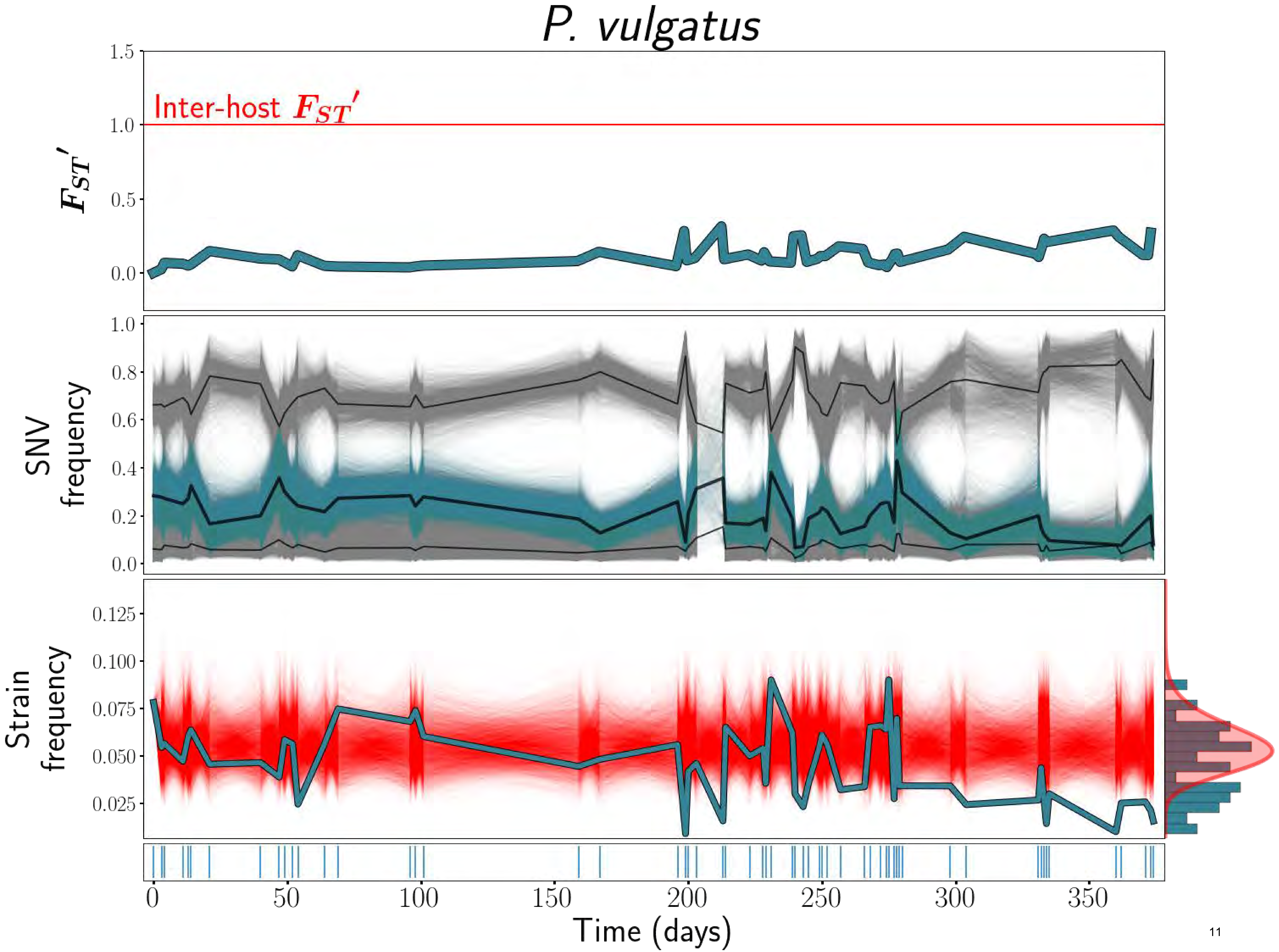
P. vulgatus
Inter-host F_{ST}'
F_{ST}'
SNV frequency
Strain frequency
Time (days)
1.5
1.0
0.5
0.0
0.8
0.6
0.4
0.2
0.125
0.100
0.075
0.050
0.025
0
50
100
150
200
250
300
350

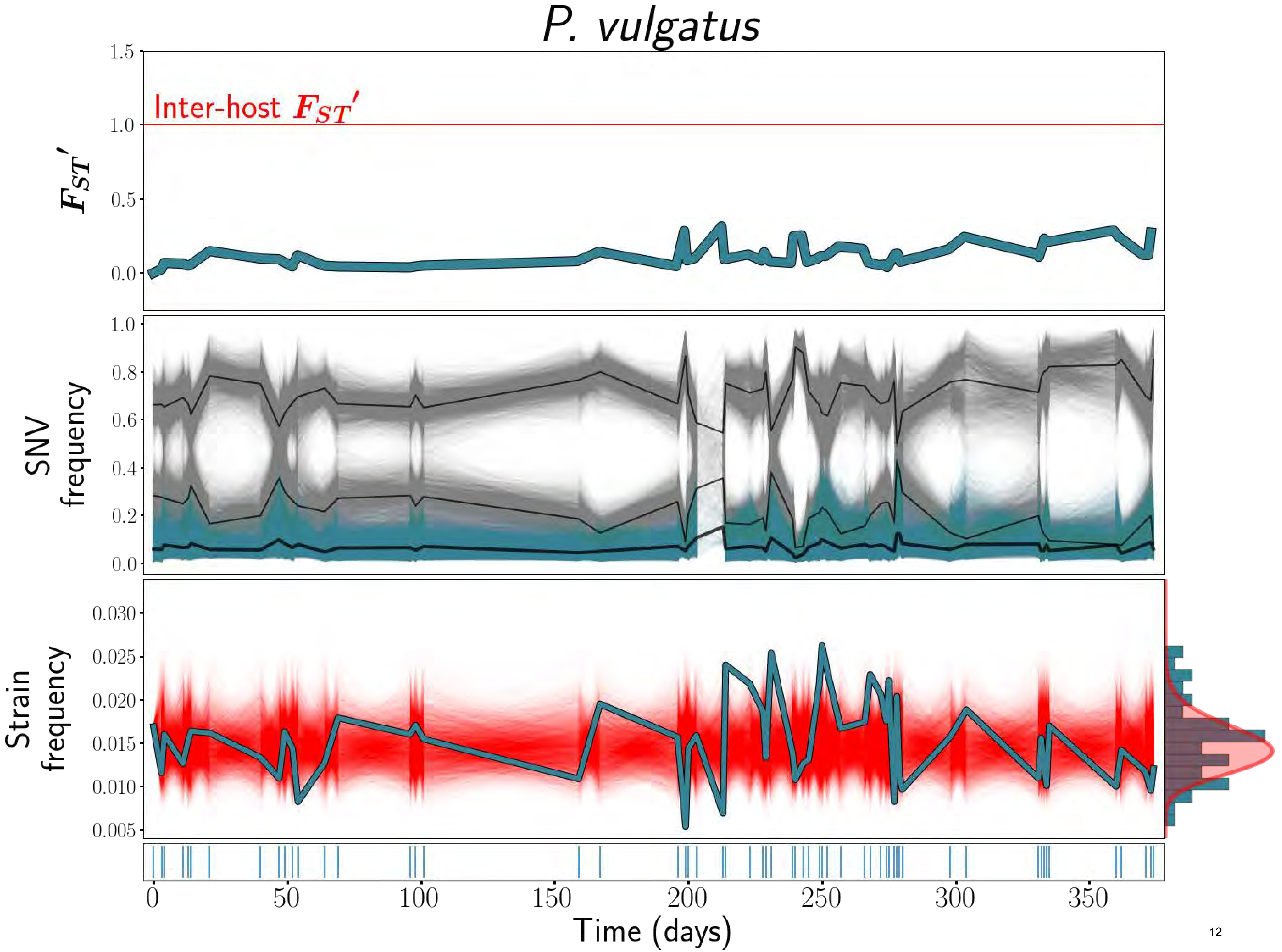
P. vulgatus
Inter-host F_{ST}'
F_{ST}'
SNV frequency
Strain frequency
Time (days)
1.5
1.0
0.5
0.0
0.8
0.6
0.4
0.2
0.030
0.025
0.020
0.015
0.010
0.005
0
50
100
150
200
250
300
350

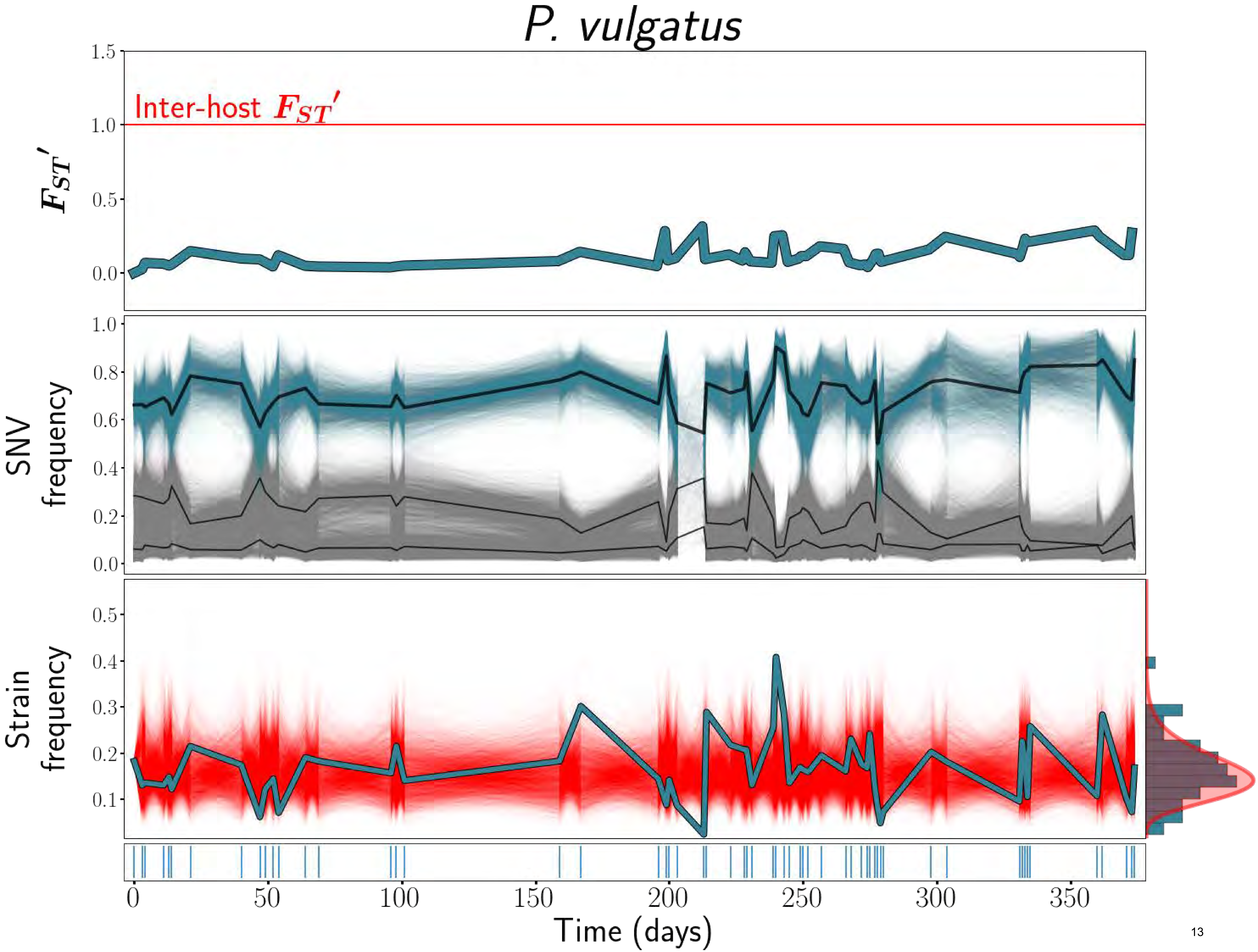

P. vulgatus
Inter-host F_{ST}'
F_{ST}'
SNV frequency
Strain frequency
Time (days)

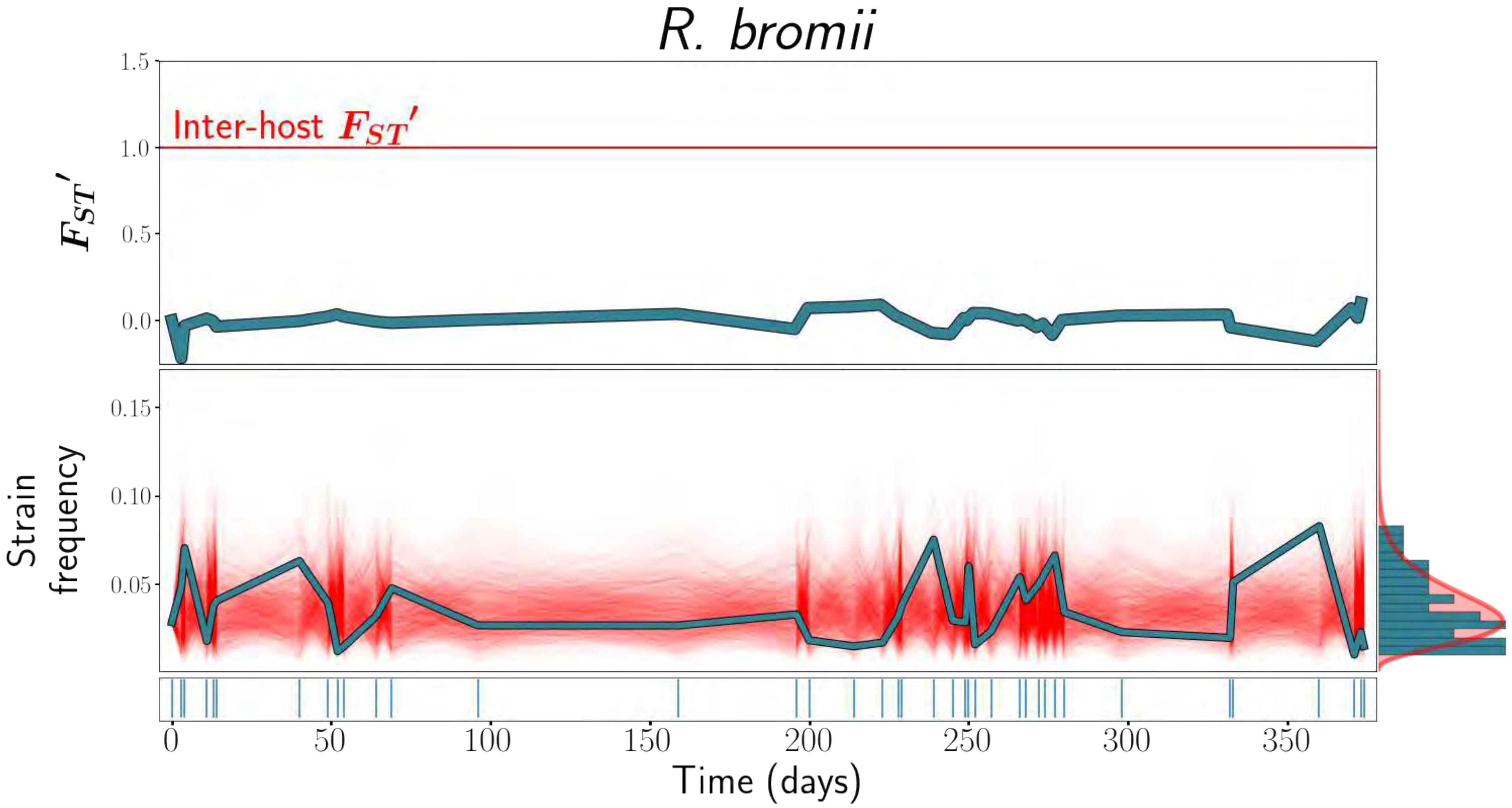

R. bromii
Inter-host F_{ST}'
F_{ST}'
1.5
1.0
0.5
0.0
Strain frequency
0.15
0.10
0.05
0
50
100
150
200
250
300
350
Time (days)

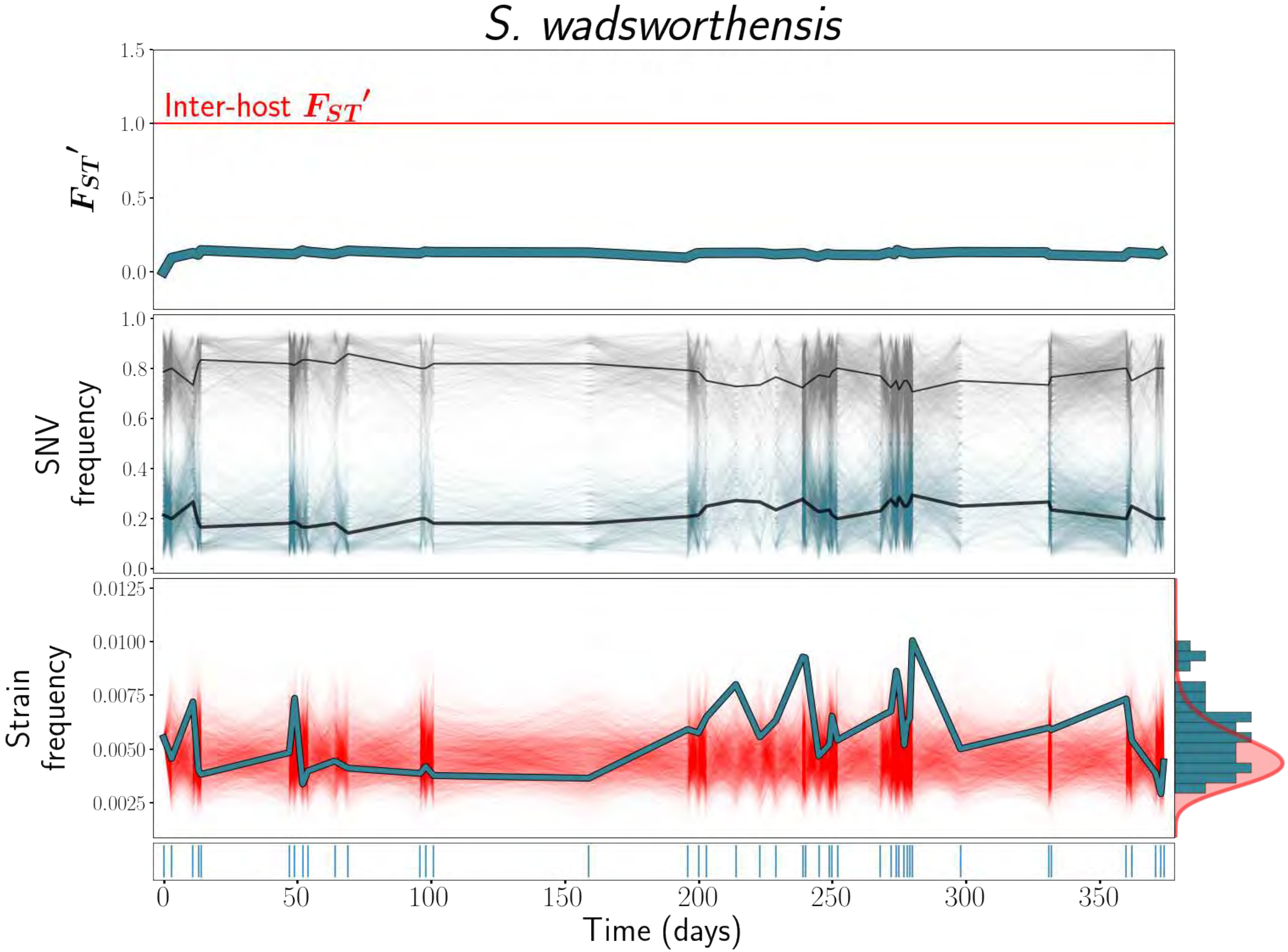

S. wadsworthensis
Inter-host F_{ST}'
F_{ST}'
SNV frequency
Strain frequency
Time (days)

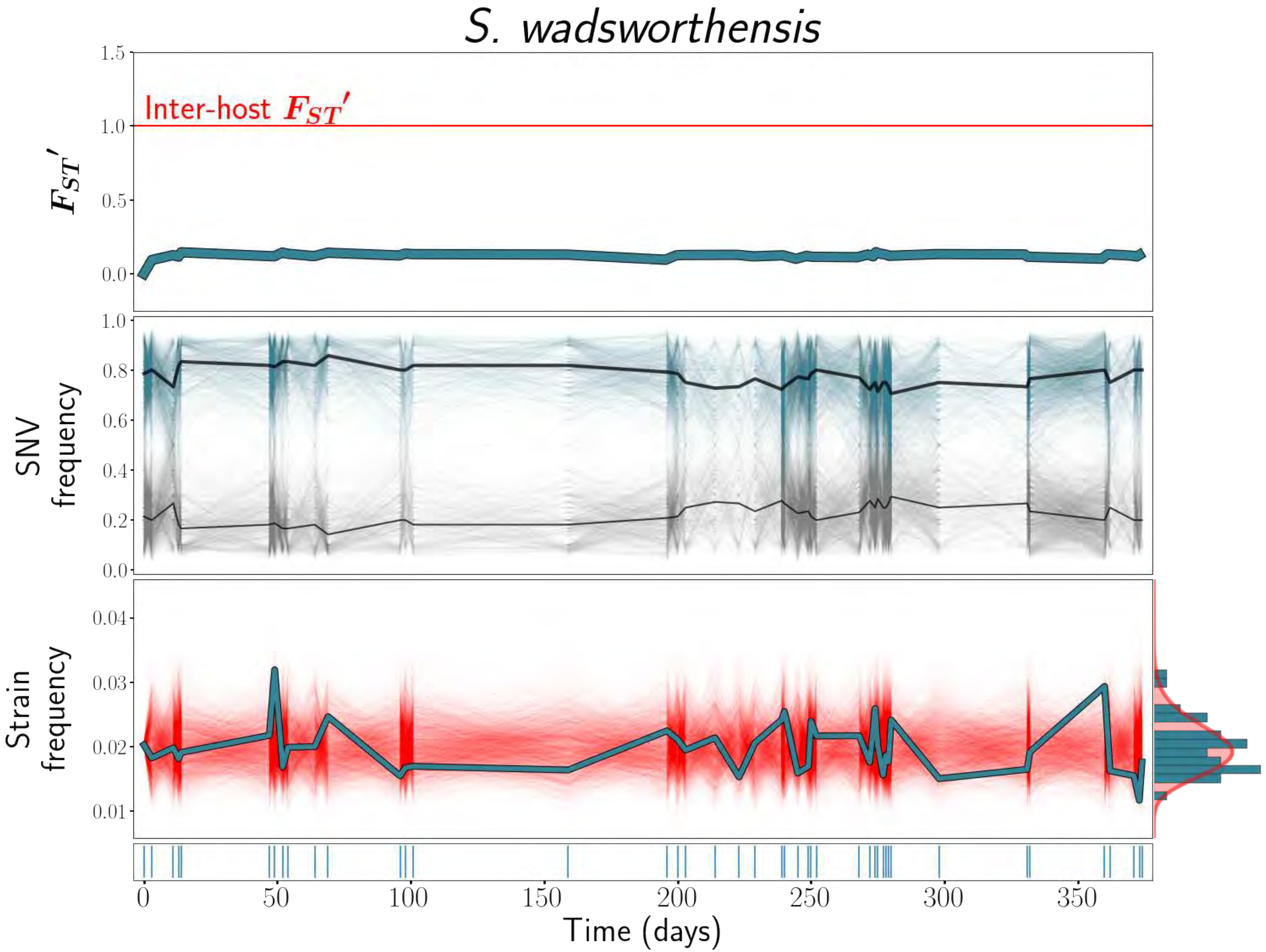
S. wadsworthensis
Inter-host F_{ST}'
F_{ST}'
SNV frequency
Strain frequency
Time (days)
1.5
1.0
0.5
0.0
0.8
0.6
0.4
0.2
0.04
0.03
0.02
0.01
0
50
100
150
200
250
300
350
