# Supplementary material for: Ecological Stability Emerges at the Level of Strains in the Human Gut Microbiome
Source: mBio. 2023 Feb 21;14(2):e02502-22. doi: 10.1128/mbio.02502-22 (PMC10127601; doi:10.1128/mbio.02502-22)
Supplement: TEXT S5 [file mbio.02502-22-s0005.pdf]

S5 Text

F_{ST}', strain frequency, and strain abundance dynamics plots for all species analyzed, for host *ae.* These plots are analogous to Main Text Figure 1. When only a single strain was detected, only *F_{ST}'* and strain abundance dynamics plots, but no strain frequency plot, is included.

Table of contents

Alistipes onderdonkii A 1
Alistipes onderdonkii B 2
Alistipes putredinis A 3
Bacteroides cellulosilyticus A 4
Bacteroides ovatus A 5
Bacteroides ovatus B 6
Bacteroides thetaiotaomicron A 7
Bacteroides uniformis A 8
Eubacterium rectale A 9
Eubacterium rectale B 10
Phocaiecola massiliensis A 11
Phocaiecola massiliensis B 12
Phocaiecola vulgatus A 13

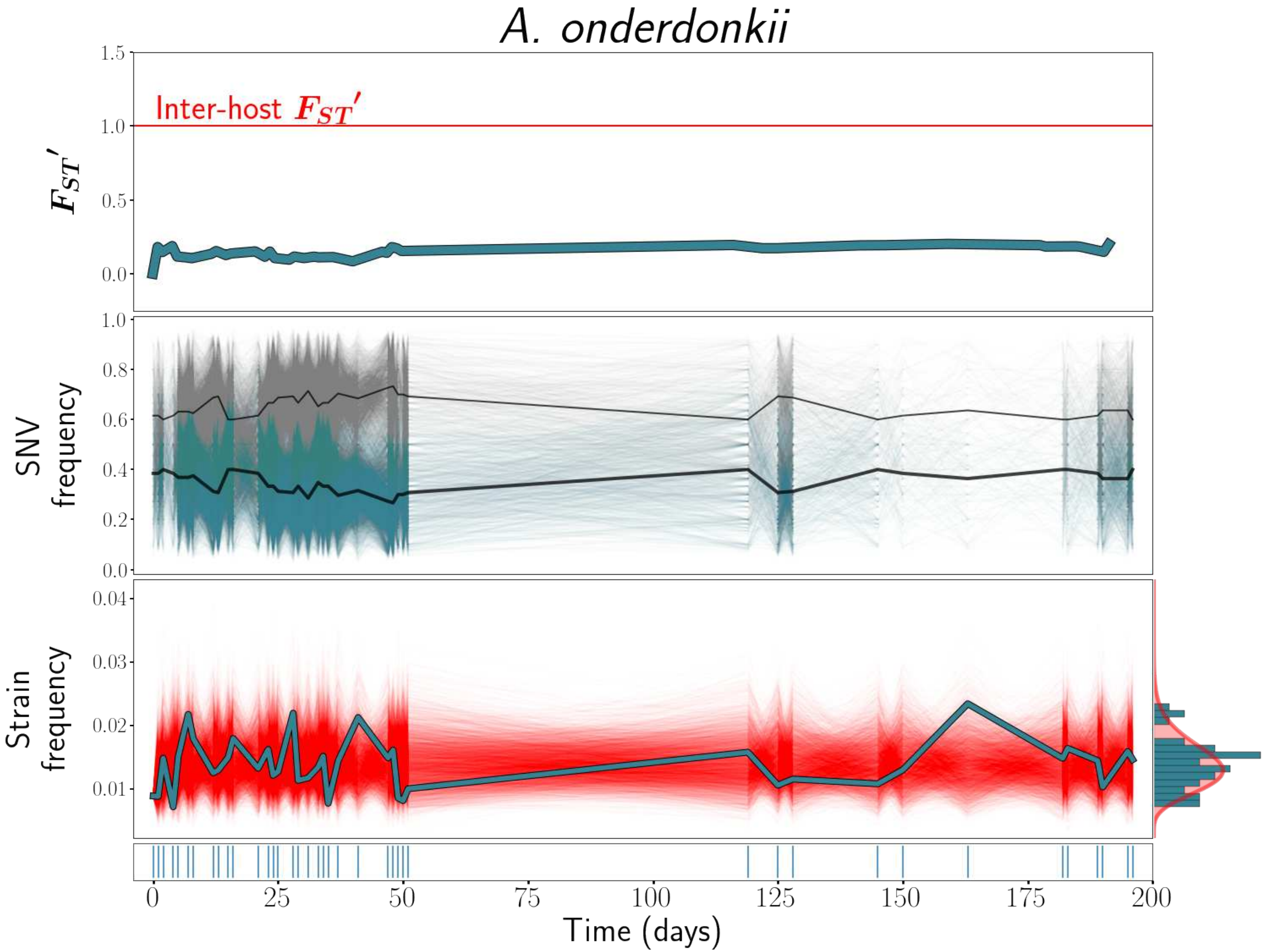
A. onderdonkii
Inter-host F_{ST}'
F_{ST}'
1.5
1.0
0.5
0.0
SNV frequency
1.0
0.8
0.6
0.4
0.2
0.0
Strain frequency
0.04
0.03
0.02
0.01
0
25
50
75
100
125
150
175
200
Time (days)

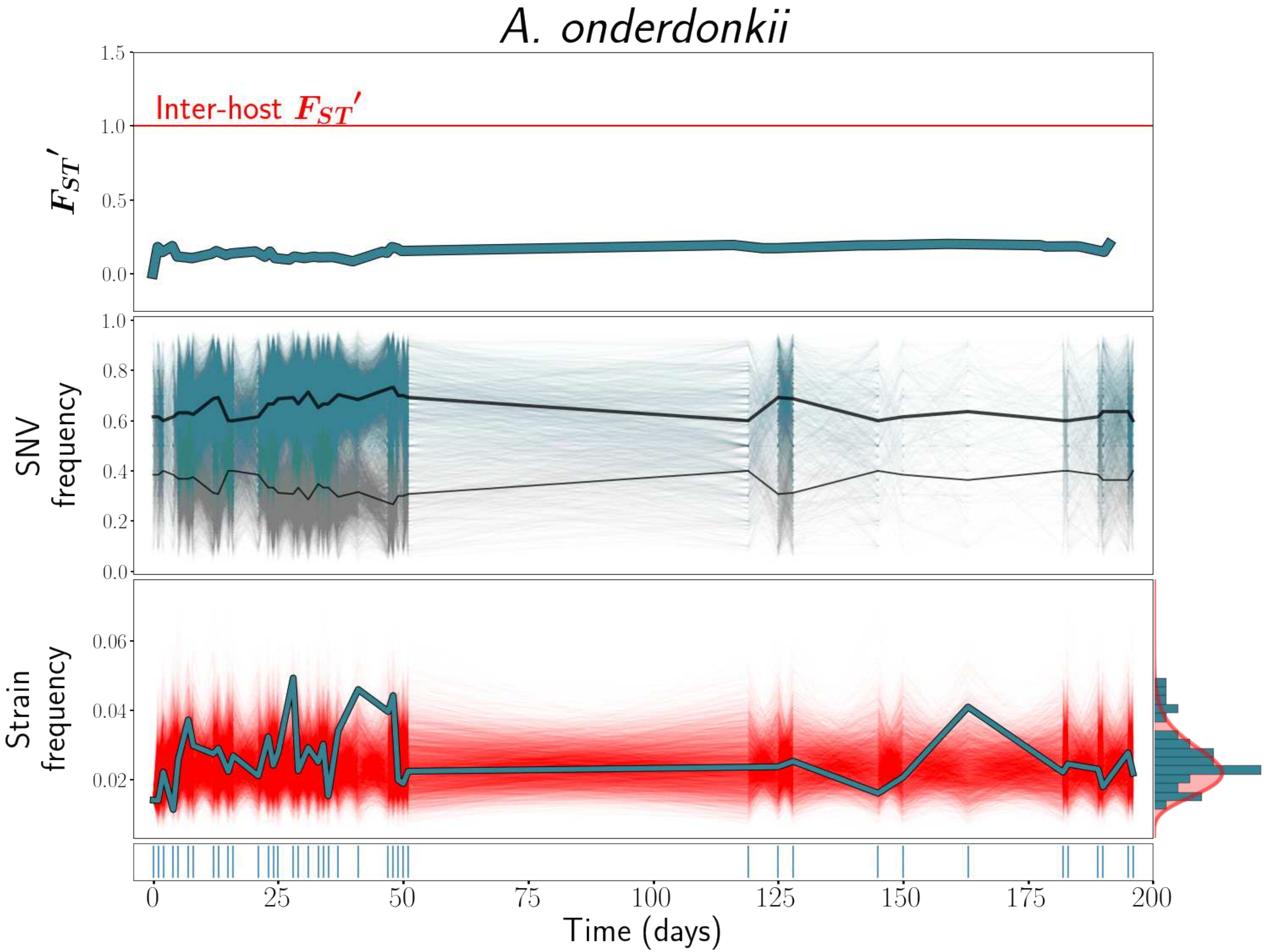
A. onderdonkii
Inter-host F_{ST}'
F_{ST}'
SNV frequency
Strain frequency
Time (days)
1.5
1.0
0.5
0.0
0.8
0.6
0.4
0.2
0.06
0.04
0.02
0
25
50
75
100
125
150
175
200

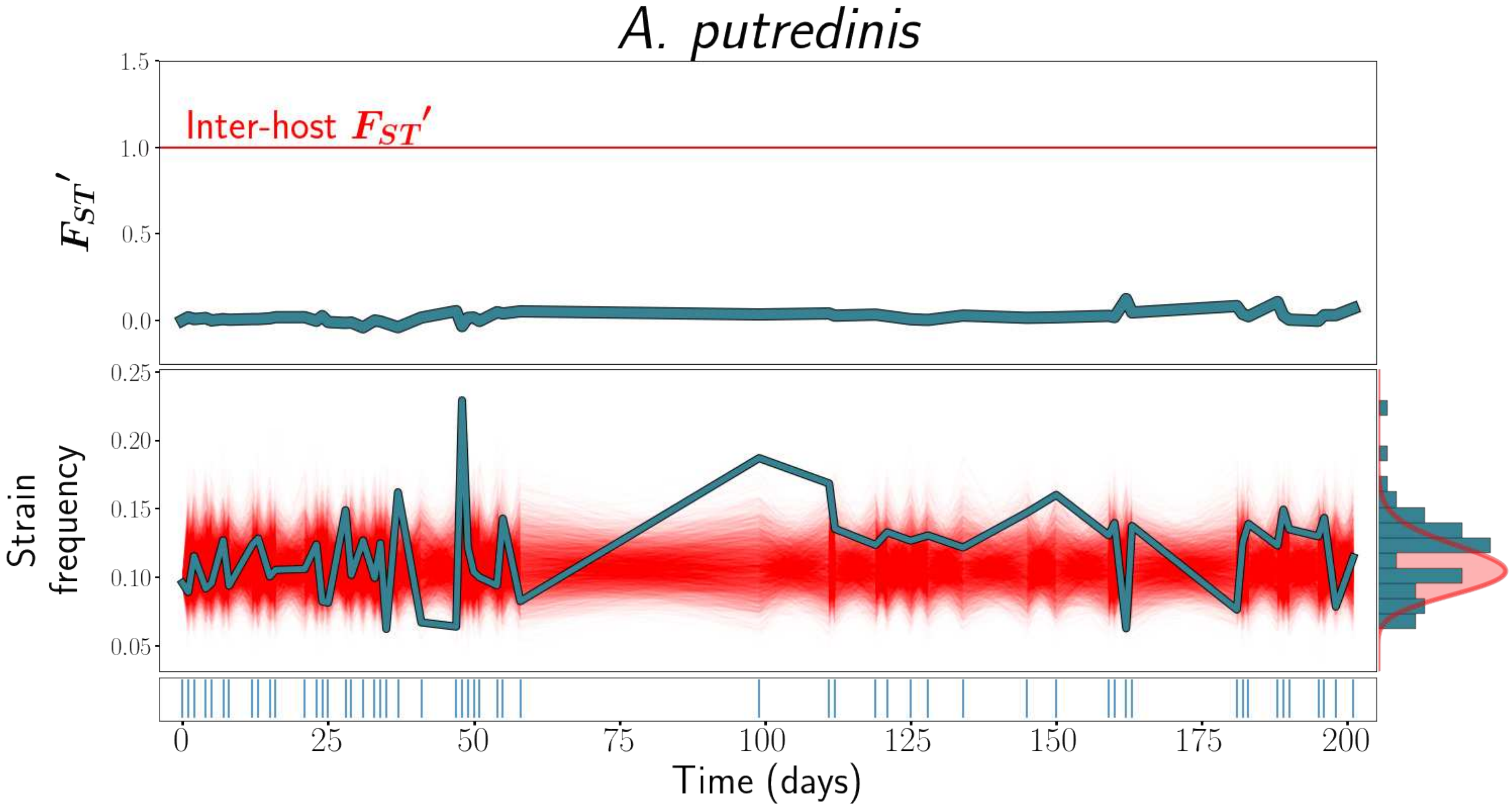

A. putredinis
Inter-host F_{ST}'
F_{ST}'
1.5
1.0
0.5
0.0
Strain frequency
0.25
0.20
0.15
0.10
0.05
0
25
50
75
100
125
150
175
200
Time (days)

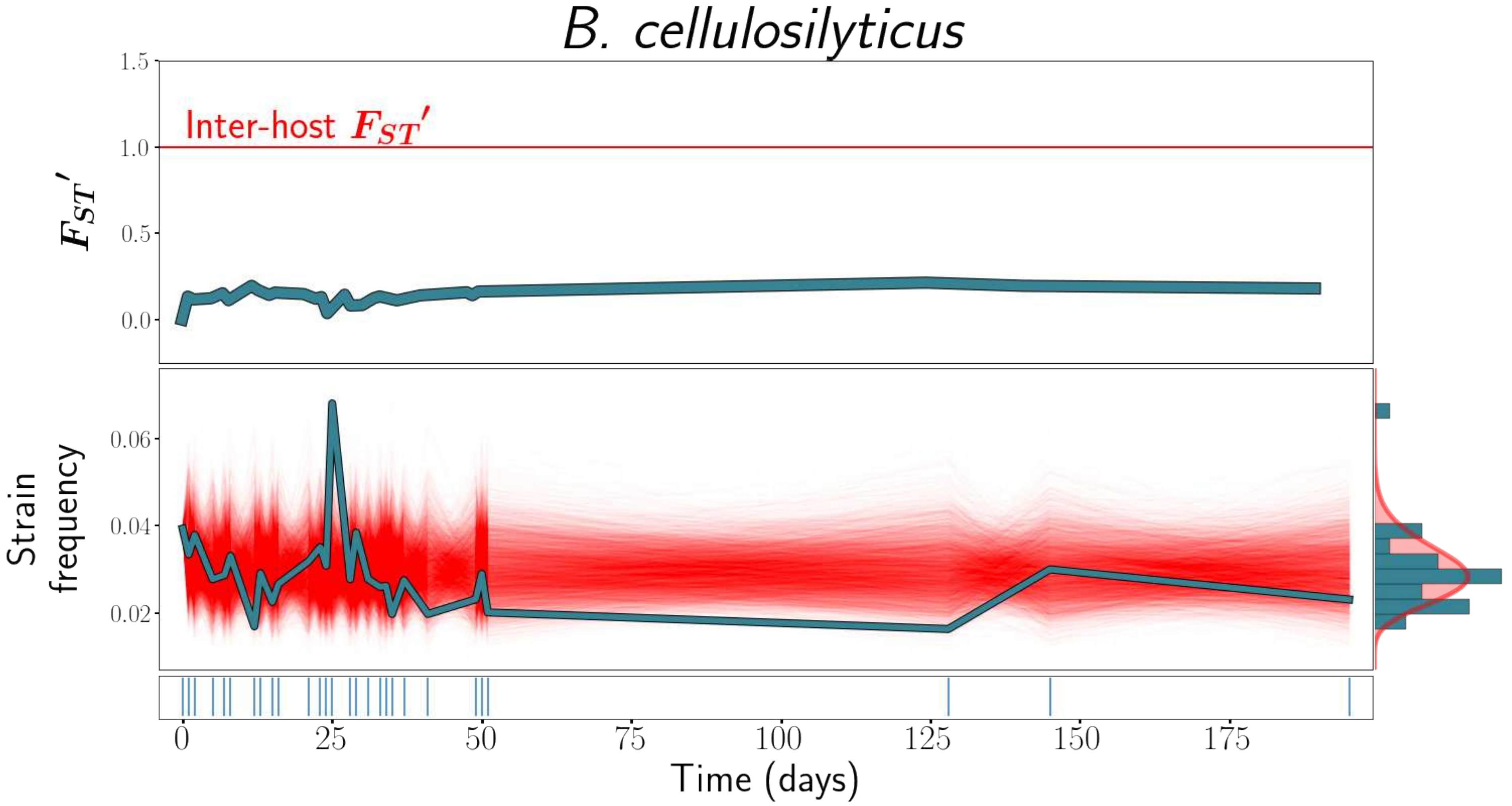

B. cellulosilyticus
Inter-host F_{ST}'
F_{ST}'
1.5
1.0
0.5
0.0
Strain frequency
0.06
0.04
0.02
0
25
50
75
100
125
150
175
Time (days)

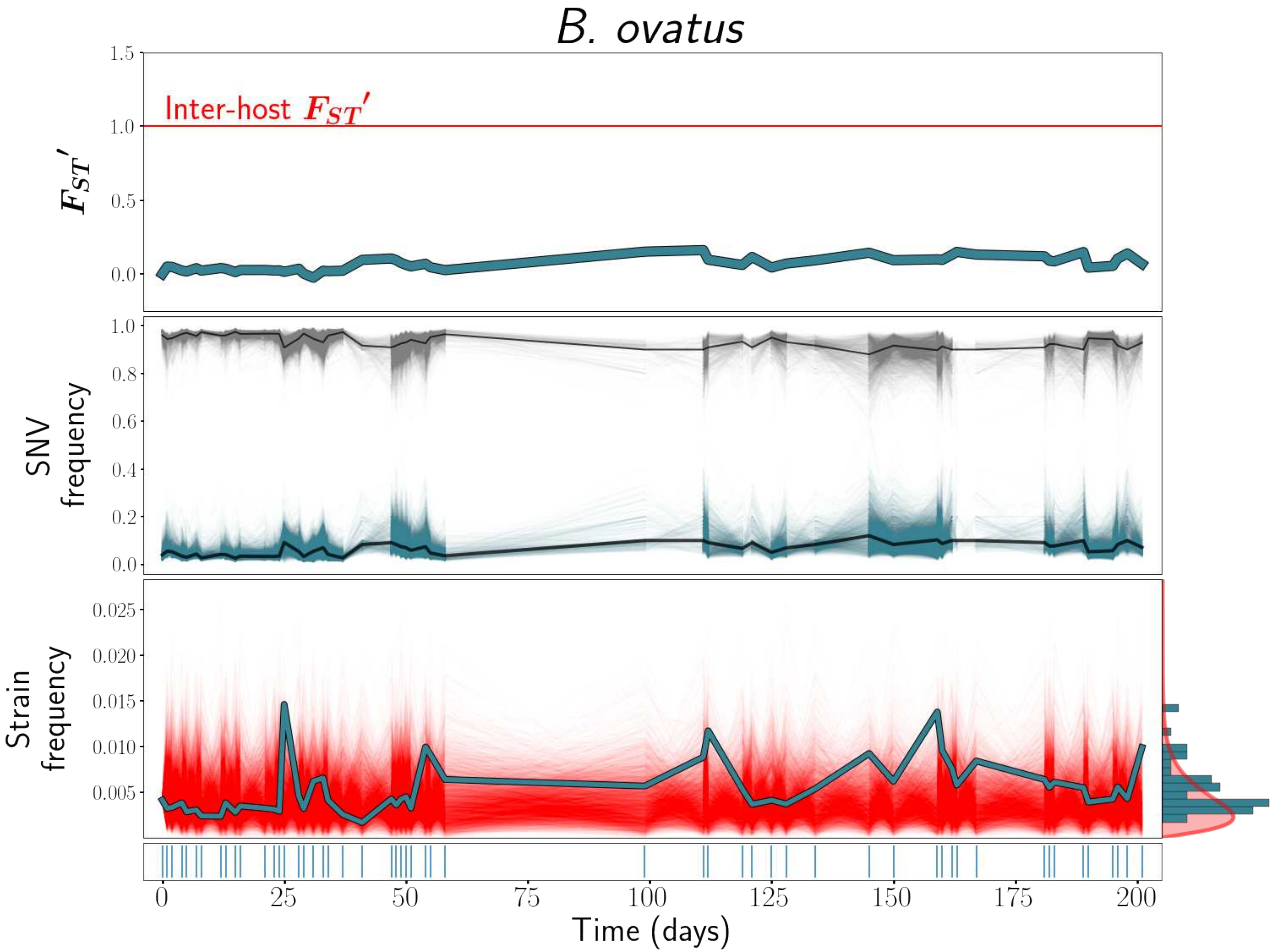

B. ovatus
Inter-host F_{ST}'
F_{ST}'
1.5
1.0
0.5
0.0
SNV frequency
1.0
0.8
0.6
0.4
0.2
0.0
Strain frequency
0.025
0.020
0.015
0.010
0.005
0
25
50
75
100
125
150
175
200
Time (days)

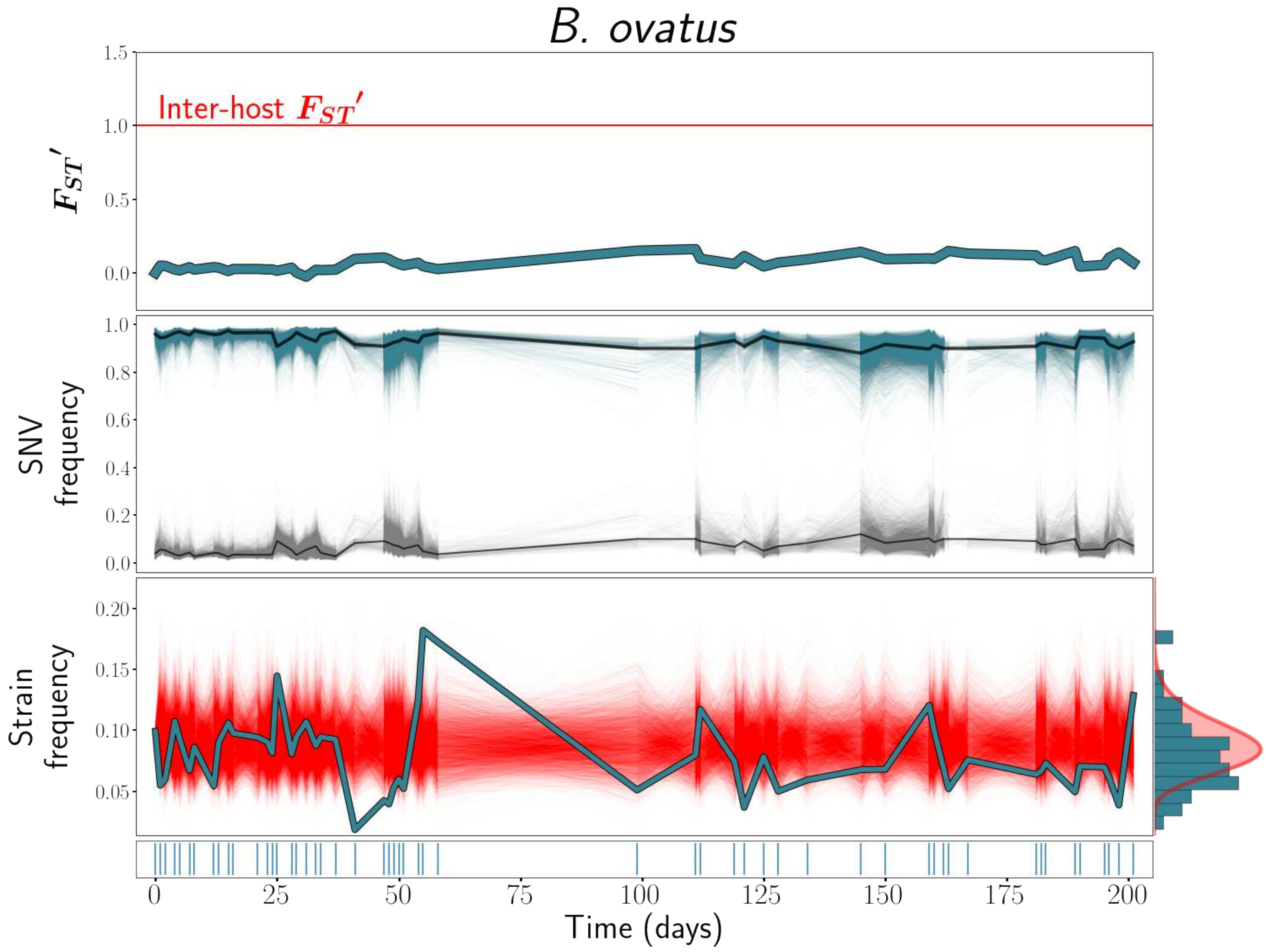
B. ovatus
Inter-host F_{ST}'
F_{ST}'
SNV frequency
Strain frequency
Time (days)

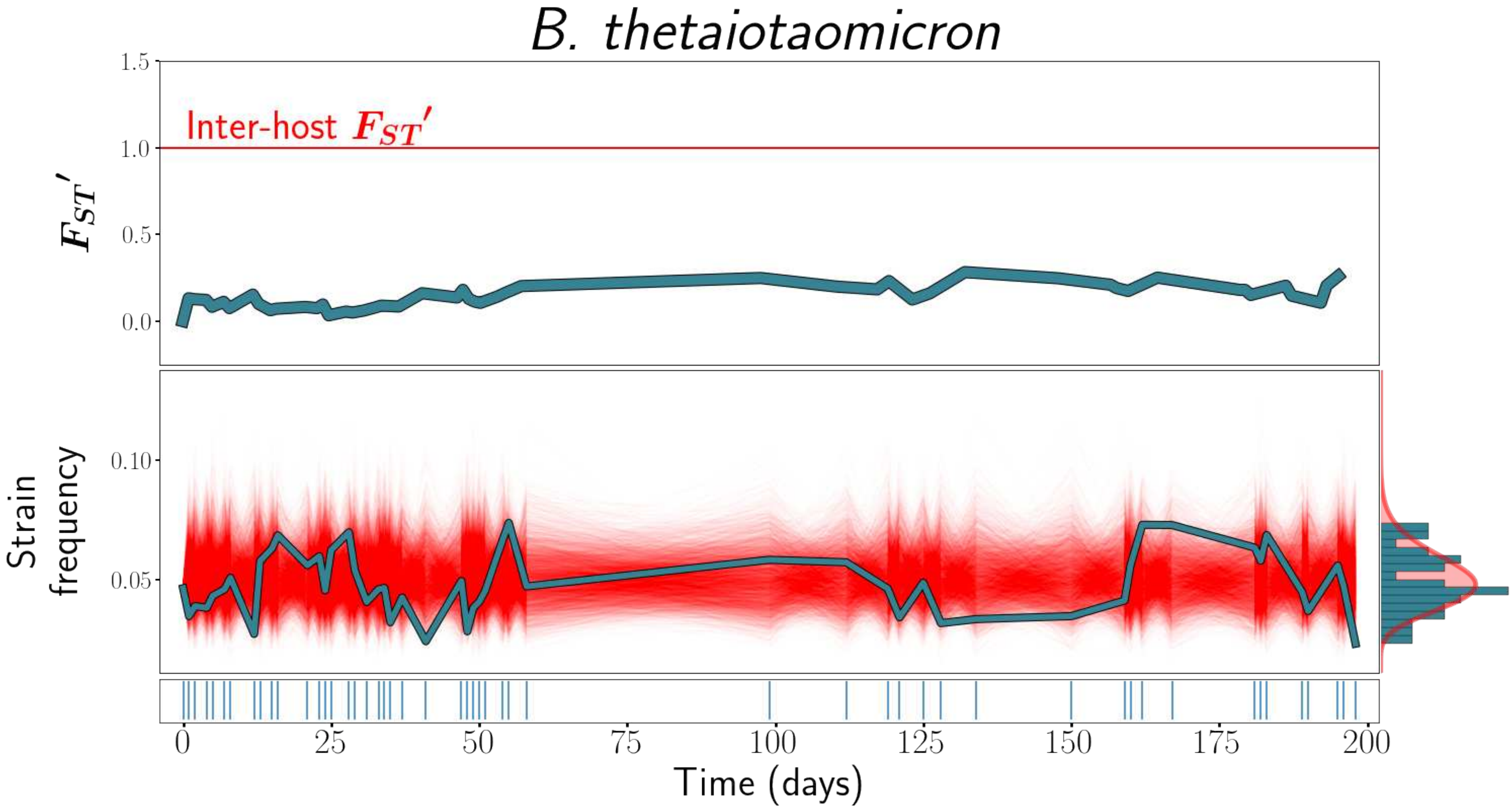

B. thetaiotaomicron
Inter-host F_{ST}'
F_{ST}'
1.5
1.0
0.5
0.0
Strain frequency
0.10
0.05
0
25
50
75
100
125
150
175
200
Time (days)

B. uniformis

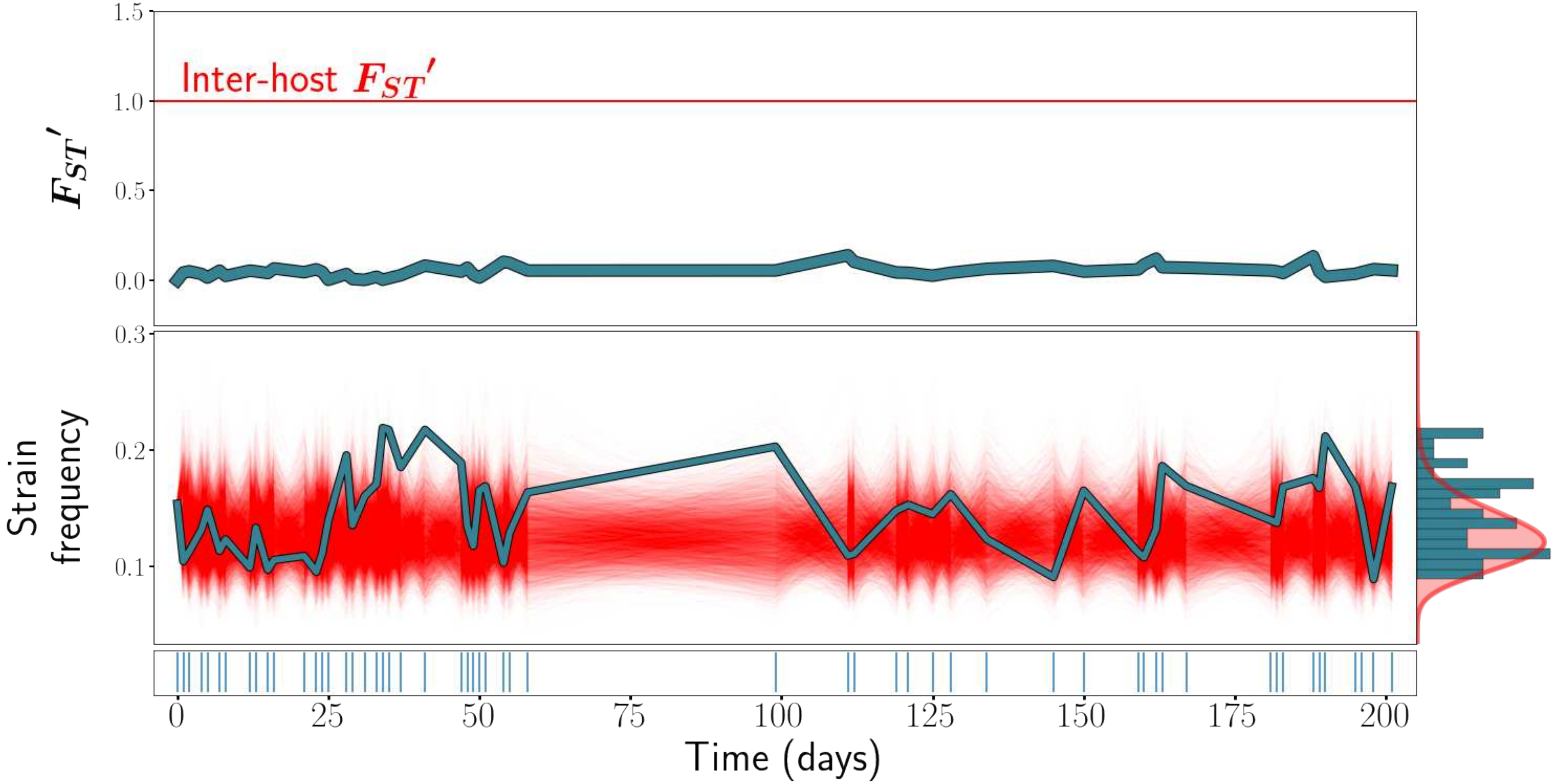

Inter-host F_{ST}'
F_{ST}'
1.5
1.0
0.5
0.0
0.3
0.2
0.1
Strain frequency
0
25
50
75
100
125
150
175
200
Time (days)

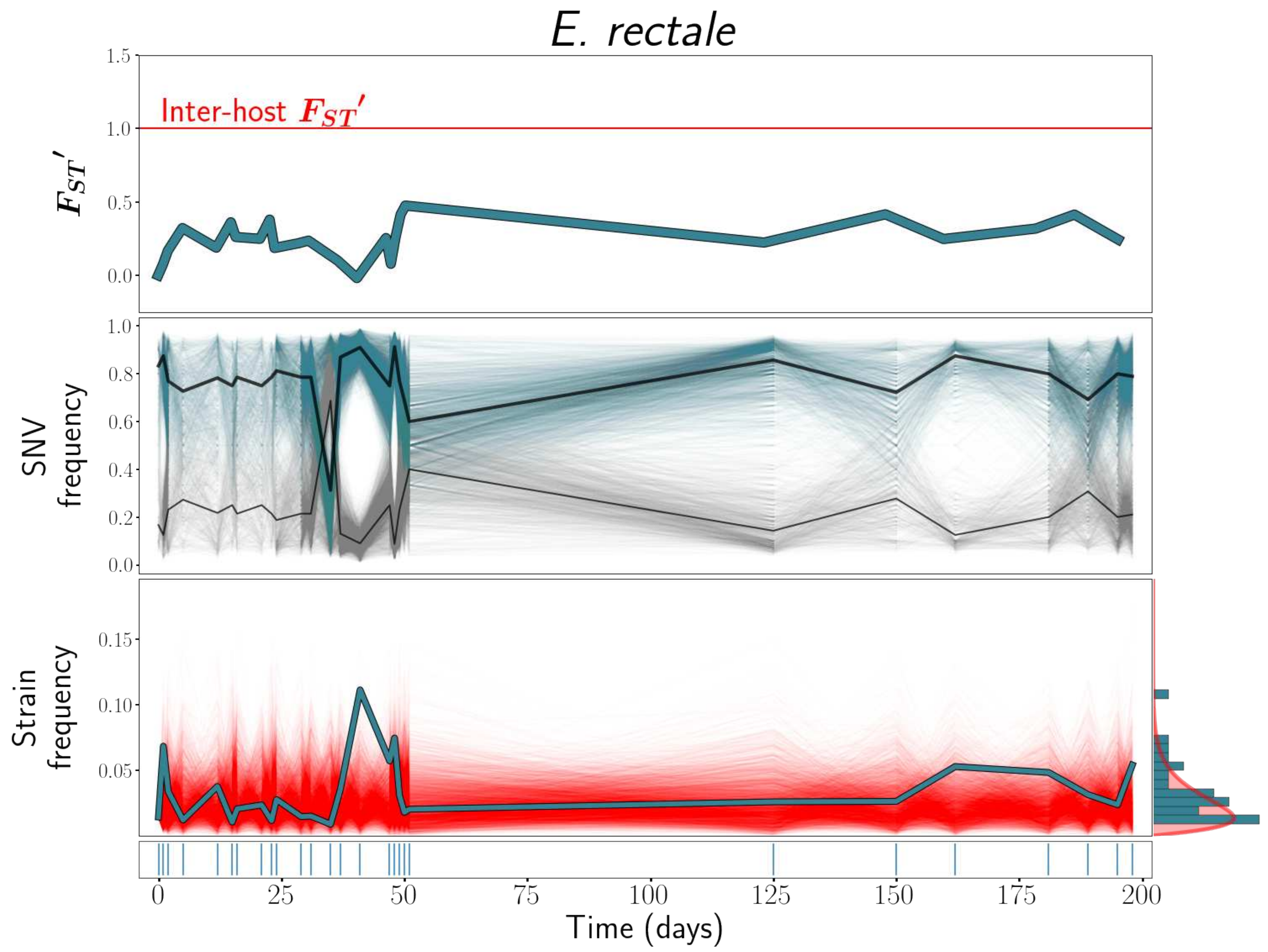
E. rectale
Inter-host F_{ST}'
F_{ST}'
1.5
1.0
0.5
0.0
SNV frequency
1.0
0.8
0.6
0.4
0.2
0.0
Strain frequency
0.15
0.10
0.05
0
25
50
75
100
125
150
175
200
Time (days)

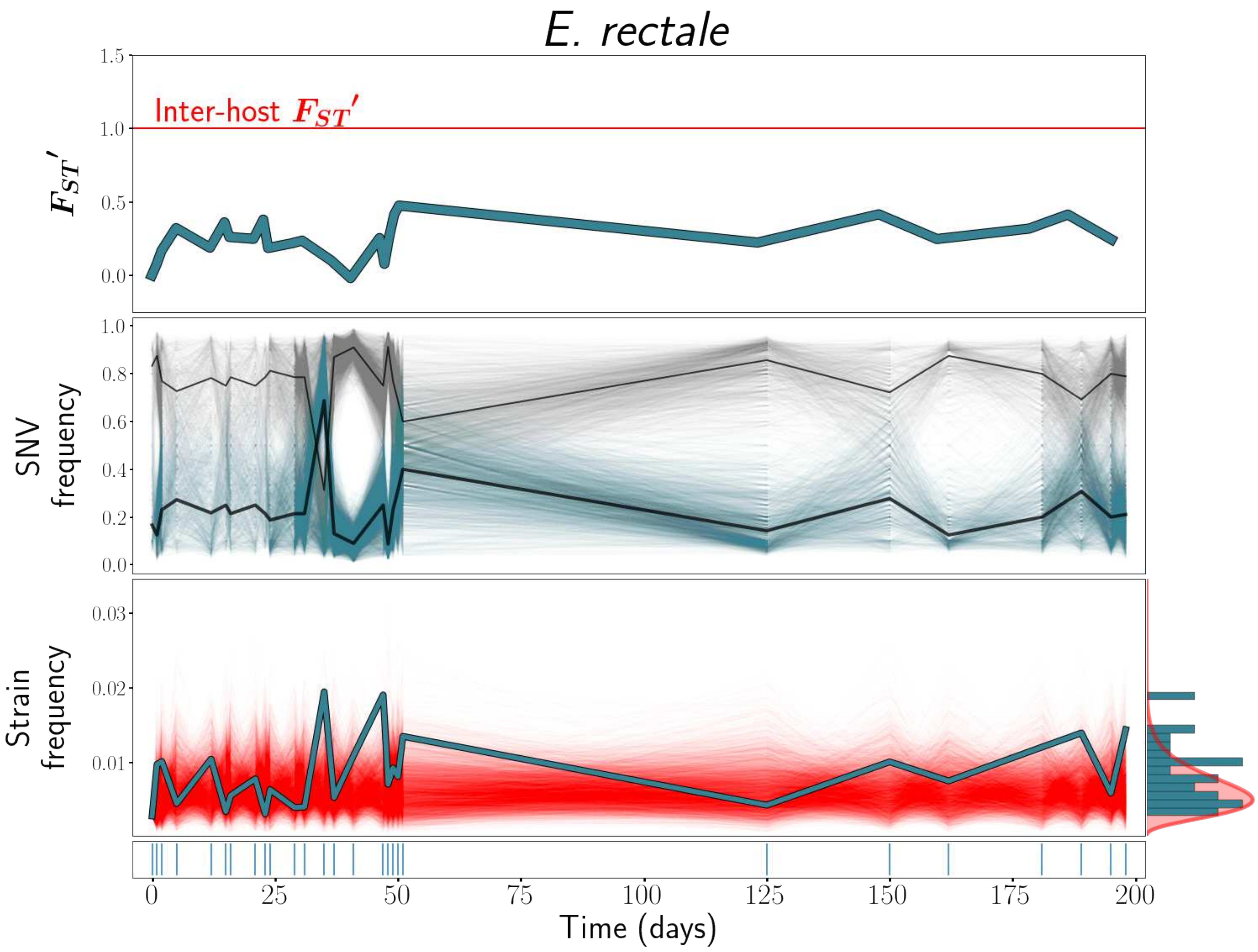
E. rectale
Inter-host F_{ST}'
F_{ST}'
SNV frequency
Strain frequency
Time (days)

P. massiliensis

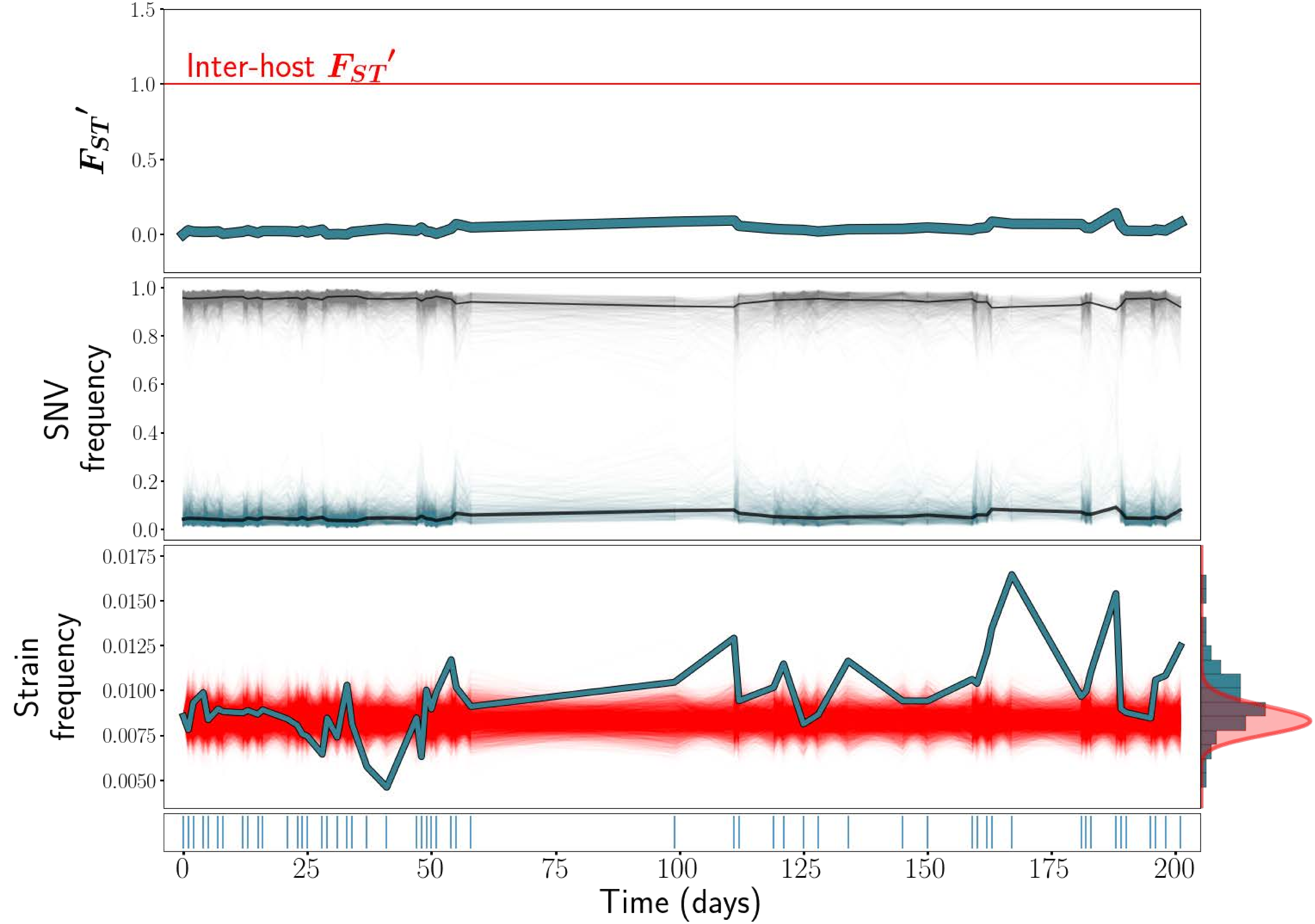

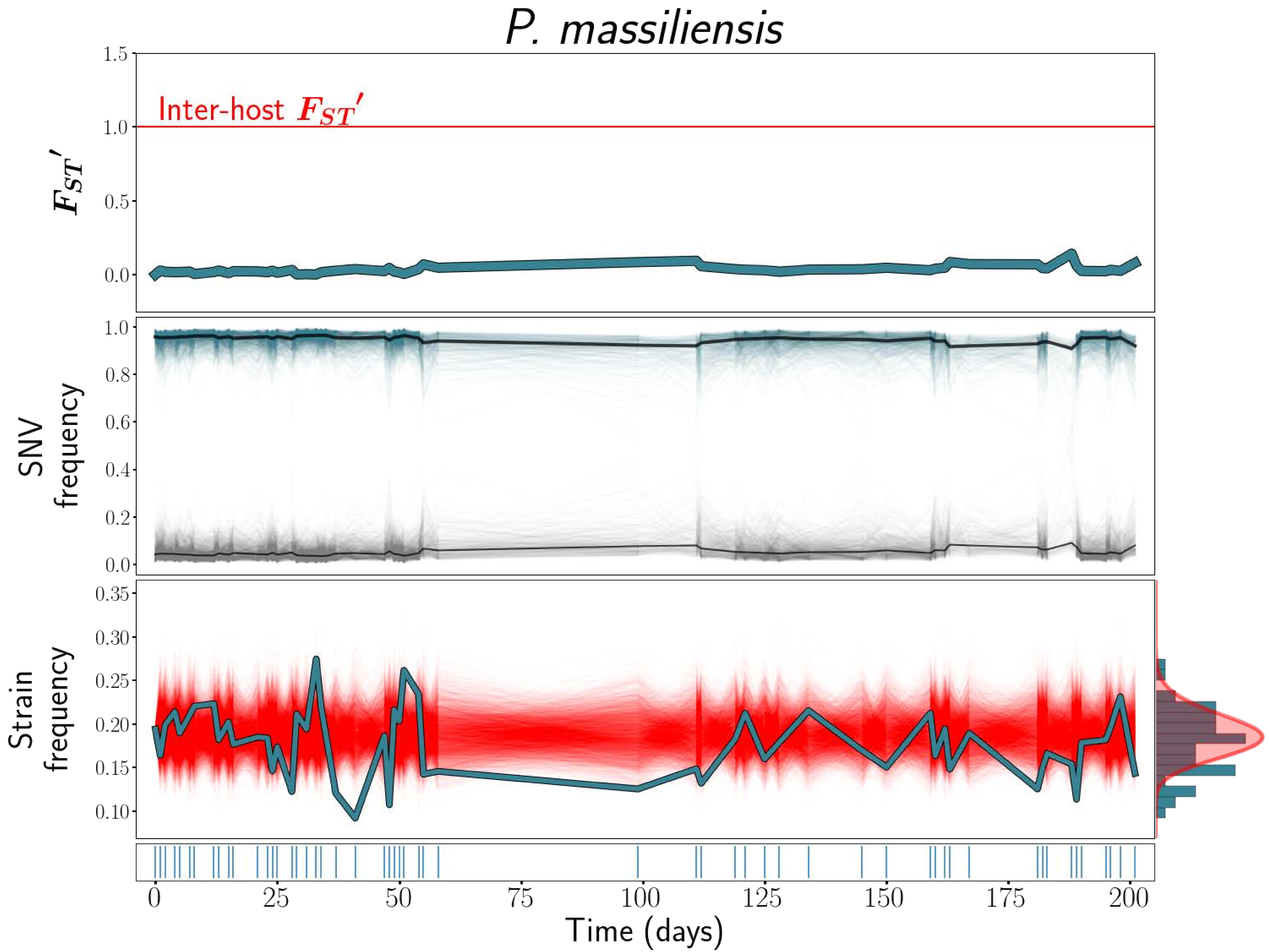
P. massiliensis
Inter-host F_{ST}'
F_{ST}'
1.5
1.0
0.5
0.0
SNV frequency
1.0
0.8
0.6
0.4
0.2
0.0
Strain frequency
0.35
0.30
0.25
0.20
0.15
0.10
0
25
50
75
100
125
150
175
200
Time (days)

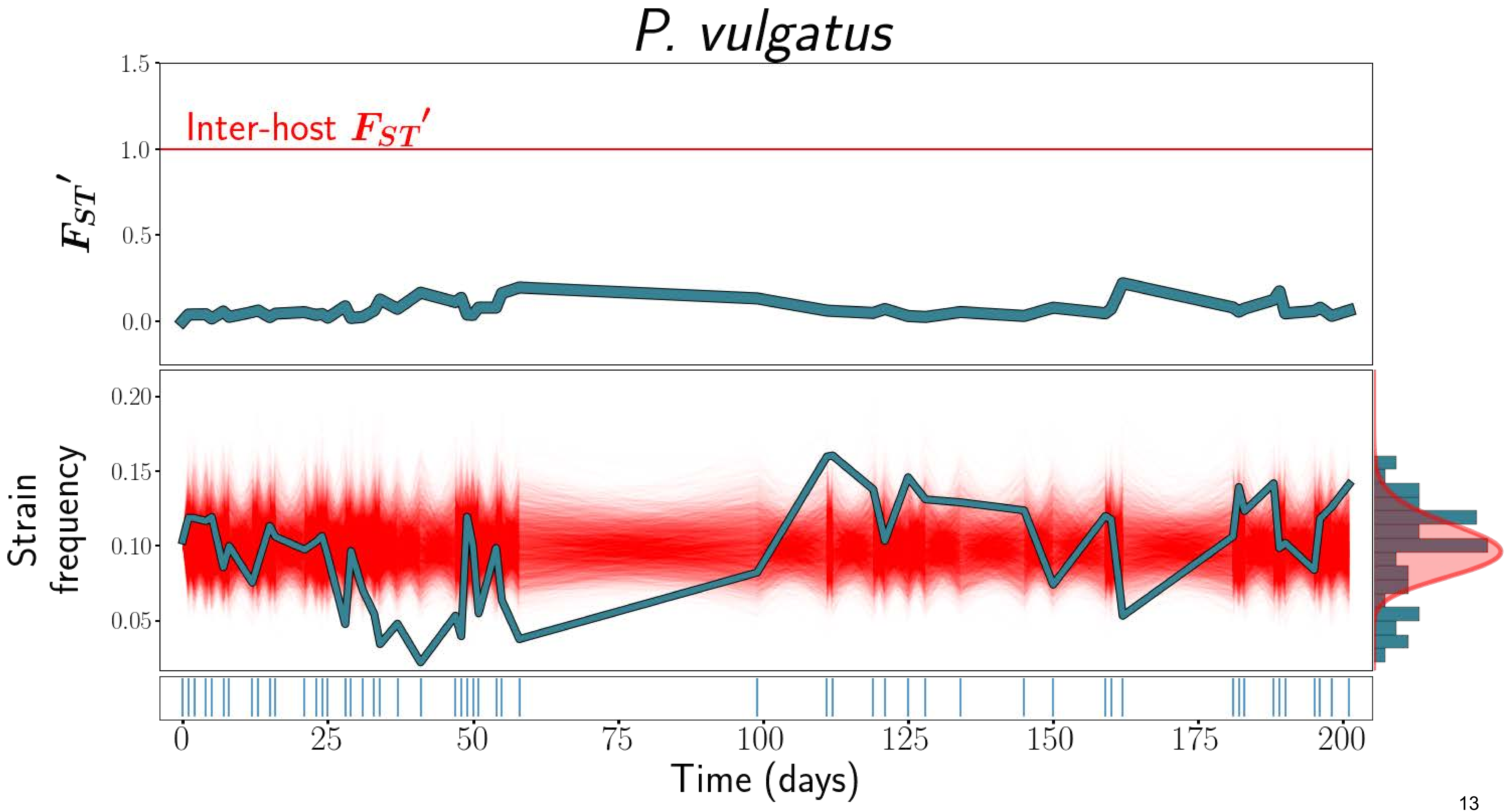

P. vulgatus
Inter-host F_{ST}'
F_{ST}'
1.5
1.0
0.5
0.0
Strain frequency
0.20
0.15
0.10
0.05
0
25
50
75
100
125
150
175
200
Time (days)
